# Cyclin D-CDK4 Disulfide Bond Attenuates Pulmonary Vascular Cell Proliferation

**DOI:** 10.1161/CIRCRESAHA.122.321836

**Published:** 2023-11-13

**Authors:** Hannah Knight, Giancarlo Abis, Manpreet Kaur, Hannah L.H. Green, Susanne Krasemann, Kristin Hartmann, Steven Lynham, James Clark, Lan Zhao, Clemens Ruppert, Astrid Weiss, Ralph T. Schermuly, Philip Eaton, Olena Rudyk

**Affiliations:** School of Cardiovascular and Metabolic Medicine and Sciences, British Heart Foundation Centre of Research Excellence (H.K., M.K., H.L.H.G., J.C., O.R.), King’s College London, United Kingdom.; Proteomics Core Facility, Centre of Excellence for Mass Spectrometry (S.L.), King’s College London, United Kingdom.; Division of Biosciences, Institute of Structural and Molecular Biology, University College London, United Kingdom (G.A.).; Institute of Neuropathology, University Medical Centre Hamburg-Eppendorf, Germany (S.K., K.H.).; National Heart and Lung Institute, Faculty of Medicine, Imperial College London, United Kingdom (L.Z.).; Universities of Giessen and Marburg Lung Center Giessen Biobank, Justus-Liebig-University Giessen, Germany (C.R.).; Department of Internal Medicine, Justus-Liebig-University Giessen, Giessen, Member of the German Center for Lung Research (DZL), Germany (A.W., R.T.S.).; William Harvey Research Institute, Barts and The London School of Medicine and Dentistry, Queen Mary University of London, United Kingdom (P.E.).

**Keywords:** cell cycle, cell proliferation, hypertension, pulmonary, myocytes, smooth muscle, oxidation-reduction

## Abstract

**BACKGROUND:**

Pulmonary hypertension (PH) is a chronic vascular disease characterized, among other abnormalities, by hyperproliferative smooth muscle cells and a perturbed cellular redox and metabolic balance. Oxidants induce cell cycle arrest to halt proliferation; however, little is known about the redox-regulated effector proteins that mediate these processes. Here, we report a novel kinase-inhibitory disulfide bond in cyclin D-CDK4 (cyclin-dependent kinase 4) and investigate its role in cell proliferation and PH.

**METHODS:**

Oxidative modifications of cyclin D-CDK4 were detected in human pulmonary arterial smooth muscle cells and human pulmonary arterial endothelial cells. Site-directed mutagenesis, tandem mass-spectrometry, cell-based experiments, in vitro kinase activity assays, in silico structural modeling, and a novel redox-dead constitutive knock-in mouse were utilized to investigate the nature and definitively establish the importance of CDK4 cysteine modification in pulmonary vascular cell proliferation. Furthermore, the cyclin D-CDK4 oxidation was assessed in vivo in the pulmonary arteries and isolated human pulmonary arterial smooth muscle cells of patients with pulmonary arterial hypertension and in 3 preclinical models of PH.

**RESULTS:**

Cyclin D-CDK4 forms a reversible oxidant-induced heterodimeric disulfide dimer between C7/8 and C135, respectively, in cells in vitro and in pulmonary arteries in vivo to inhibit cyclin D-CDK4 kinase activity, decrease Rb (retinoblastoma) protein phosphorylation, and induce cell cycle arrest. Mutation of CDK4 C135 causes a kinase-impaired phenotype, which decreases cell proliferation rate and alleviates disease phenotype in an experimental mouse PH model, suggesting this cysteine is indispensable for cyclin D-CDK4 kinase activity. Pulmonary arteries and human pulmonary arterial smooth muscle cells from patients with pulmonary arterial hypertension display a decreased level of CDK4 disulfide, consistent with CDK4 being hyperactive in human pulmonary arterial hypertension. Furthermore, auranofin treatment, which induces the cyclin D-CDK4 disulfide, attenuates disease severity in experimental PH models by mitigating pulmonary vascular remodeling.

**CONCLUSIONS:**

A novel disulfide bond in cyclin D-CDK4 acts as a rapid switch to inhibit kinase activity and halt cell proliferation. This oxidative modification forms at a critical cysteine residue, which is unique to CDK4, offering the potential for the design of a selective covalent inhibitor predicted to be beneficial in PH.

**Novelty and Significance**

**What Is Known?**

- Cyclin D-CDK4/6 is hyperactive in human pulmonary artery hypertension (PAH), which contributes to the hyperproliferative phenotype of vascular cells.
- Altered redox environments are reported in PAH due to upregulated NOX enzymes, downregulated superoxide dismutase enzymes and metabolic changes.
- Oxidants induce cell cycle arrest by regulating cell signaling pathways, however many of the effector proteins have not been identified.

**What New Information Does This Article Contribute?**

- Cyclin D-CDK4 is a redox-regulated protein complex that forms an intermolecular disulfide bond between CDK4 C135 and cyclin D C7/8 to inhibit kinase activity toward its substrate, Rb.
- CDK4 C135 is an allosteric site that is indispensable for kinase activity, meaning perturbation of this thiol, through oxidation or mutation, impairs kinase activity to attenuate cell proliferation and reduce pulmonary vascular disease progression.
- Abundance of the cyclin D-CDK4 disulfide is decreased in the pulmonary arteries and human pulmonary artery smooth muscle cells of PAH patients, while potentiation of the disulfide is therapeutically beneficial in 3 models of experimental PAH.

Hyperproliferation of vascular cells, due to cell cycle reprogramming, drives vascular remodeling in PAH. Importantly, the cell cycle can be regulated by the redox environment of cells through oxidative modifications of effector proteins. Here, we describe a novel intermolecular disulfide bond that forms between CDK4 C135 and cyclin D C7/8 to inhibit kinase activity toward its key substrate, Rb. CDK4 C135 acts as a critical cysteine residue that allosterically regulates catalytic activity, meaning perturbation of this residue attenuates the proliferation of cells. Previous reports suggest that cyclin D-CDK4 is hyperactive in PAH patients, which we show may be driven, at least partially, by a decrease in formation of the cyclin D-CDK4 disulfide bond. Potentiation of disulfide accumulation, through the repurposing of drugs which inhibit redox cycling, such as auranofin, provides therapeutic benefits in experimental PAH, potentially offering a novel therapeutic approach for this disease. Additionally, future development of a selective, covalent kinase inhibitor of CDK4 C135, may represent a unique drug class to induce cell cycle arrest.

**In This Issue, see p 963**

**Meet the First Author, see p 964**

Vascular remodeling is a prominent structural hallmark of pulmonary hypertension (PH) that involves changes in all layers of the vessel wall. Combined with chronic vasoconstriction, remodeling causes a sustained increase in pulmonary vascular resistance and pressure, eventually leading to heart failure and death.^1–3^ Endothelial cells, smooth muscle cells, and fibroblasts isolated from the lungs of patients with PH, display an increased proliferative potential, dedifferentiated phenotype, and altered metabolism.^4–8^ Indeed, vascular cells from PH patients resemble similarities to cancer cells, including adapted cellular energetics and resistance to apoptosis, as well as hyperproliferation.^4,9^ Despite smooth muscle cell proliferation being among the most significant in PH pathology, there remains a lack of detailed understanding of underlying signaling pathways, resulting in a limited number of antiproliferative treatment options. The prognosis for PH remains poor due to an unmet clinical need for disease-modifying treatments that target the underlying hyperproliferative phenotype.^10,11^

Group 1 pulmonary arterial hypertension (PAH) and group 3 PH are associated with an altered redox environment due to increased expression of NOX (NADPH oxidase) enzymes and uncoupling of the electron transport chain, among other causes.^12–15^ Accordingly, some evidence points toward a causative role of reactive oxygen species (ROS) in vascular remodeling^12–14^ through posttranslational oxidative modifications of proteins that contain reactive cysteine thiols.^16,17^ NEDD9 (neural precursor cell expressed, developmentally downregulated 9) oxidation impairs binding to SMAD3 (small mothers against decapentaplegic homolog 3) causing maladaptive pulmonary vascular fibrosis.^18^ Additionally, oxidative stress has been shown to inhibit the endothelin-B receptor through a redox-switch to decrease nitric oxide (NO) synthesis and promote PAH.^19^ Mammalian cells are highly compartmentalized to optimize redox signaling and cellular functions under certain redox steady-states, and the exact effect of ROS in PH is likely to depend on the source of production, microenvironment and the cellular compartment the protein resides.^20^ One example is the EGFR (epidermal growth factor receptor), a membrane protein known to be activated by redox modification of a critical active site cysteine.^21^ Ligand-induced activation of EGFR induces H_2_O_2_ production by nearby NOX complexes, further enhancing tyrosine kinase activity through cysteine sulfenation.^21^ Although the direct involvement of this EGFR cysteine sulfonation in PH has not yet been demonstrated, an alternative redox-regulated ligand-independent mechanism of EGFR activation, through H_2_O_2_-induced tyrosine dimerization, has been reported in experimental PH and patients with Group 1 PAH.^22^

While it was previously thought that increased expression of the H_2_O_2_-producing enzyme, NOX4, was causative in the pathogenesis of PH,^13^ studies in NOX4^−/−^ mice revealed no protection from disease.^23^ Numerous recent reports suggest that ROS may contribute to adaptive mechanisms that attenuate disease severity.^16,23,24^ SOD (superoxide dismutase), which catalyzes the dismutation of superoxide to produce H_2_O_2_, is downregulated in PAH patients and SOD expression was shown to be protective in preclinical models of PH.^25–27^ These findings support a protective role of H_2_O_2_^25–27^ through reversible oxidative modifications, including the formation of a disulfide bond between 2 cysteine thiols, which can serve as a redox-switch to regulate protein function and rapidly fine-tune cellular signaling.^17,28,29^ This is exemplified by a disulfide bond in the BMPR2 ligand, BMP9 (bone morphogenic protein 9), which increases protein stability and signaling to maintain endothelial homeostasis.^30^ In addition, our previous work shows that the formation of a kinase-activating intermolecular disulfide bond in PKGIα (protein kinase G Iα) promotes vasodilation^31^ and attenuates endothelial-to-mesenchymal transition to provide protection against the development of severe PH.^16,24^ Furthermore, we showed an adaptive intermolecular bond in PKARIα (protein kinase A regulatory subunit RIα) regulates angiogenesis^32^ and promotes systemic vasodilation,^33^ although follow-up work is required to address the role of this kinase in PH.

Further importance of redox fine-tuning in PH can be demonstrated in SERCA (sarco/endoplasmic reticulum calcium [Ca^2+^] ATPase), which maintains Ca^2+^ homeostasis by transporting it from the cytoplasm to sarcoplasmic reticulum. Nitric oxide–dependent vascular smooth muscle relaxation is mediated by reversible S-glutathionylation of SERCA, which activates the pump and decreases intracellular Ca^2+^ concentration.^34^ However, irreversible SERCA oxidation to sulfonic acid prevents protein activation by S-glutathionylation, which impairs nitric oxide–induced vasodilation.^34^ It was recently reported that chronic hypoxia increases irreversible SERCA oxidation, and heterozygous redox-dead SERCA2 C674S KI mice that cannot undergo SERCA S-glutathionylation, develop age-dependent pulmonary vascular remodeling due to increased pulmonary vascular cell proliferation.^35^

Recent reports suggest that cell cycle dysregulation is causative in the development of PH.^4,36,37^ Increased secretion of growth factors such as PDGF (platelet-derived growth factor), together with upregulation of PDGF receptor-β expression,^1^ results in sustained mitogenic signaling through the P13K (phosphoinositide 3-kinase)/Akt (protein kinase B)/mTOR (mammalian target of rapamycin) pathway, and MAPK (mitogen-activated protein kinase) pathway. Additionally, dysregulation of transforming growth factor-β/SMAD-dependent signaling, as well as increased expression and activity of cyclin-dependent kinases (CDKs), ultimately converge to promote progression of the cell cycle and facilitate proliferation of pulmonary vascular cells.^1,9,36–41^ Notably, the food and drug administration–approved CDK4/6 inhibitor, palbociclib, was recently reported to reverse the progression of PH in 2 preclinical models through a decrease in pulmonary arterial muscularization.^36^ CDK4 is a serine/threonine kinase that forms an active heterodimeric complex with cyclin D to regulate the G1 phase of the cell cycle.^42^ Phosphorylation of Rb (retinoblastoma) protein by cyclin D-CDK4 releases the transcription factor, E2F, to increase the expression of S-phase cell cycle proteins.^43^ Classically, cyclin D-CDK4 activity is regulated by mitogenic stimulation of cyclin D expression,^44,45^ as well as phosphorylation of CDK4 T172 by CDK-activating kinase, and binding of proteins including inhibitors of CDK4 (INK4) and CIP/KIP inhibitors.^42,43,46^

To date, little is known regarding the redox-regulated effector proteins that respond to ROS fluctuations to control the cell cycle, and what physiological or pathophysiological role they may have. Here, we describe a novel intermolecular disulfide bond in cyclin D-CDK4 that inhibits kinase activity to regulate proliferation of pulmonary vascular cells. The abundance of the CDK4 disulfide is decreased in pulmonary arteries and pulmonary arterial smooth muscle cells from patients with idiopathic PAH; while therapeutic benefits are provided by the pharmacological inhibition of redox cycling using a thioredoxin reductase inhibitor, which increases disulfide accumulation in experimental models of PH. We provide evidence for the cyclin D-CDK4 disulfide bond serving as a protective mechanism that slows proliferation of pulmonary vascular smooth muscle cells to attenuate pathological remodeling, which ultimately offers CDK4 oxidation as a potential therapeutic target for PH.

## METHODS

### Data Availability

For additional methods and supplemental figures please see the Supplemental Material. Affymetrix data are deposited at Gene Expression Omnibus (https://www.ncbi.nlm.nih.gov/geo/query/acc.cgi) with accession number GSE244830.

### Cell Culture and Treatments

Primary human pulmonary arterial smooth muscle cells (HPASMCs) were purchased from ScienCell Research Laboratories (No. 3110). HPASMCs were cultured in DMEM (DMEM, Thermo Fisher Scientific No. 11965092), supplemented with 10% fetal bovine serum (FBS, ScienCell No. 0500) and 1% penicillin/streptomycin (100 U/mL) at 37 °C and 5% CO_2_. This medium will be referred to as DMEM growth medium in experiments. All experiments were performed with cells between passages 5 and 8. Primary pulmonary vascular smooth muscle cells were isolated from pulmonary arteries of explanted lungs obtained from 6 patients with idiopathic PAH undergoing lung transplantation (mean age±SD, 43.2±16.3 years; 3 male and 3 female patients), while control cells were obtained from six donors (mean age±SD, 47.8±11.5 years; 3 men and 3 women). Cells were obtained in frame of the European IPF registry (eurIPFreg) and provided by the UGMLC Giessen Biobank, member of the DZL Platform Biobanking. Informed consent was obtained in written form from each subject. The study protocol was approved by the Ethics Committee of the Justus-Liebig-University School of Medicine (No. 111/08 and 58/15). PAH and control HPASMCs were cultured in smooth muscle cell growth medium-2 (PromoCell, No. C-39262). All experiments were performed in DMEM growth medium with cells of passage 2 to 4. Primary human pulmonary arterial endothelial cells were purchased from PromoCell (No. C-12241) and were cultured in Endothelial Cell Growth Medium-2 (PromoCell, No. C-22211). WT and CDK4 KO human near-haploid cell line (HAP1) cells were purchased from Horizon Discovery (No. HZGHC000044c011, Cambridge, United Kingdom). The CDK4 KO was commercially obtained by introducing a 5 base pair deletion in exon 2 using CRISPR/cas9. HAP1 cells were cultured in Iscove’s Modified Dulbecco’s Medium (IMDM No. 12440053) supplemented with 10% FBS (PAN Biotech No. P40-39500) and 1% penicillin/streptomycin. MCF7 breast epithelial cells and HeLa cells were cultured in DMEM growth medium supplemented with 10% FBS (PAN Biotech No. P40-39500) and 1% penicillin/streptomycin.

Cells were treated with H_2_O_2_ (Merck No. H1009) at 70% to 80% confluency for 15 minutes using a 10 mmol/L H_2_O_2_ stock, which was prepared in ultrapure H_2_O immediately before experimentation. HPASMCs were exposed to UV irradiation, using a 365 nm UVA lamp for 10 or 30 minutes. HPASMCs were treated with auranofin (Enzo Life Sciences, No. BML-EI206) using a 1 mmol/L stock in 20% DMSO/H_2_O.

In all experiments, cells were lysed using nonreducing sample buffer containing 100 mmol/L maleimide. Samples were analyzed by immunoblotting under nonreducing or reducing (supplemented with 5% β-mercaptoethanol) conditions. Antibodies were purchased from Cell Signaling Technology for CDK4 (No. 12790), cyclin D1 (No. 2922), cyclin D3 (No. 2936), Rb (No. 9309), pRb S780 (No. 8180), pRb S795 (No. 9301), pRb S807/811 (No. 8516), vinculin (No. 13901), GAPDH (No. 2118), FLAG-tag (No. 2368), HA-tag (No. 3724), anti-mouse HRP-linked secondary (No. 7076), anti-rabbit HRP-linked secondary (No. 7074). In mouse tissues or human tissues, CDK4 (H-22) primary antibody from Santa Cruz (No. sc-601) was used. Representative immunoblots are representative of the results that are the most similar to mean values.

### Human Idiopathic PAH Samples

Human pulmonary artery samples were obtained from 9 patients with idiopathic PAH (mean age±SD, 38±5.2 years; 4 male and 5 female patients). Cardiac measurements were obtained by right heart catheterization. Mean pulmonary arterial pressure of these patients was 52.1±5.6 mm Hg; mean systolic arterial pressure, 78±9.2 mm Hg; and 6-minute walking distance, 369±25 m. Eight nonutilized donor pulmonary arteries served as controls (mean age±SD, 50.0±6.1 years; 3 men and 5 women). All pulmonary artery samples were collected in frame of the European IPF registry (eurIPFreg) and provided by the Universities of Giessen and Marburg Lung Center Giessen Biobank, member of the DZL Platform Biobanking. Informed consent was obtained in written form from each subject. The study protocol was approved by the Ethics Committee of the Justus-Liebig-University School of Medicine (No. 111/08 and 58/15). All PAH diagnoses were made according to the WHO guidelines.

### Animal Studies

All animal procedures were performed in accordance with the Home Office Guidance on the Operation of the Animals (Scientific Procedures) Act 1986 in the United Kingdom and were approved by the King’s College London Animal Welfare and Ethical Review Body. Age- and body weight–matched male C57BL/6J mice or male Wistar-Kyoto rats were purchased from Charles River Laboratories (United Kingdom) and were maintained at the on-site Biological Services Unit. Animals were kept in pathogen-free conditions, had ad libitum access to standard chow and water, and were kept in a 12-hour day/night cycle at 20 to 22 °C, 60% humidity. PH was modeled by exposing animals to normobaric hypoxia or Sugen (SU5146)/hypoxia, as described in more detail in Supplemental Material. All animals that met the inclusion criteria were included in the study. Mice in all studies were randomly assigned to experimental or treatment groups using a random number generator by GraphPad randomization method. Male and female mice were randomized separately. Rats were randomly assigned to different experimental groups by alternating animals to each group.

### Generation of Constitutive C135A CDK4 KI Mice

Mice constitutively expressing C135A CDK4 were generated for us on a pure C57BL/6NT background by Taconic Biosciences as following. Superovulated C57BL/6NTac females were mated with C57BL/6NTac males. One cell stage fertilized embryos were isolated from the oviducts at 0.5 days post coitus (dpc). Zygotes were electroporated with CRISPR ribonucleoprotein mix in a NEPA21 Type II electroporator using a NepaGene CUY501P1-1.5 electrode (Xceltis) to introduce the C135A mutation into exon 4 of Cdk4 using a specific guide RNA and oligonucleotide for homology-directed repair. After recovery, 25 to 35 injected one-cell stage embryos were transferred to the oviducts of 0.5 dpc, pseudo pregnant Swiss Webster female mice to produce F0 founder animals. Genomic DNA was extracted from biopsies and analyzed by PCR. An aliquot of the PCR reaction was used to validate the presence of the homology-directed repair–introduced restriction site MfeI. PCR products of the restriction-positive animals were subcloned and sequenced to estimate homology-directed repair mosaicism (60%–100%).

In vitro fertilization was performed using oocytes from superovulated C57BL/6NTac females and sperm from mutant males. After overnight incubation, 2 cell embryos were transferred into oviducts of 0.5 dpc pseudopregnant Swiss Webster recipient females to generate F1 heterozygous animals. The presence of the C135A CDK4 point mutation and the absence of modification at potential off-target sites was confirmed by sequencing of genomic DNA.

### Statistical Analysis

All data were analyzed using GraphPad Prism 9 and are presented as dot-plots with mean±SEM, and precise *P* values provided on graphs. Statistical parametric testing was performed based on data distribution. For samples with n≥6, the Shapiro-Wilk normality test was first applied. To obtain *P* values, the data with n≥6 that passed this normality test were analyzed by either parametric unpaired and 2-tailed *t* test with Welch correction for 2 groups, by parametric 1-way ANOVA for >2 groups, or by 2-way ANOVA for comparisons of multiple conditions applied to 2 or more groups, adjusted for multiple comparisons using Tukey post hoc test, unless otherwise stated in Figure legends. As the data sample size with n<6 cannot be reliably tested for normality, to obtain *P* values for samples with n<6, nonparametric tests were applied, either a 1-way ANOVA Kruskal-Wallis test for >2 groups, adjusted for multiple comparisons using Dunn post hoc test, or nonparametric unpaired 2-tailed Mann-Whitney *U* test when 2 groups were being compared. For all analyses, a *P* value of ≤0.05 was considered significant, except for some comparisons of samples with n=3, where it is indicated that the minimum achievable *P* value of 0.1000 was reached using the nonparametric test.

The sample size required to achieve statistical significance was estimated using a power calculation based on prior experience of anticipated differences between groups and group variance, 95% confidence level, probability of type-I error 5%, and probability of type-II error 20%. For experimental PH studies, the minimum number of animals for adequate study power was calculated based on the dichotomous primary end point, such as whether the animal developed a RV to left ventricular mass ratio on average 30% higher than in healthy adult control animal population, or not. Based on our prior experience, for any control group, this would occur in <5% of all animals, and for any experimental PH model used, it would occur in at least 80% of animals. As a result, a minimal number of at least 5 animals per group was required to complete the study. Where indicated, the sample size was larger, based on animal availability or when both male and female species were used. All in vivo experiments were performed according to ARRIVE guidelines for reporting animal research.^47^

## RESULTS

### CDK4 Forms a Reversible Intermolecular Disulfide Bond With Cyclin D

**Figure 1.**
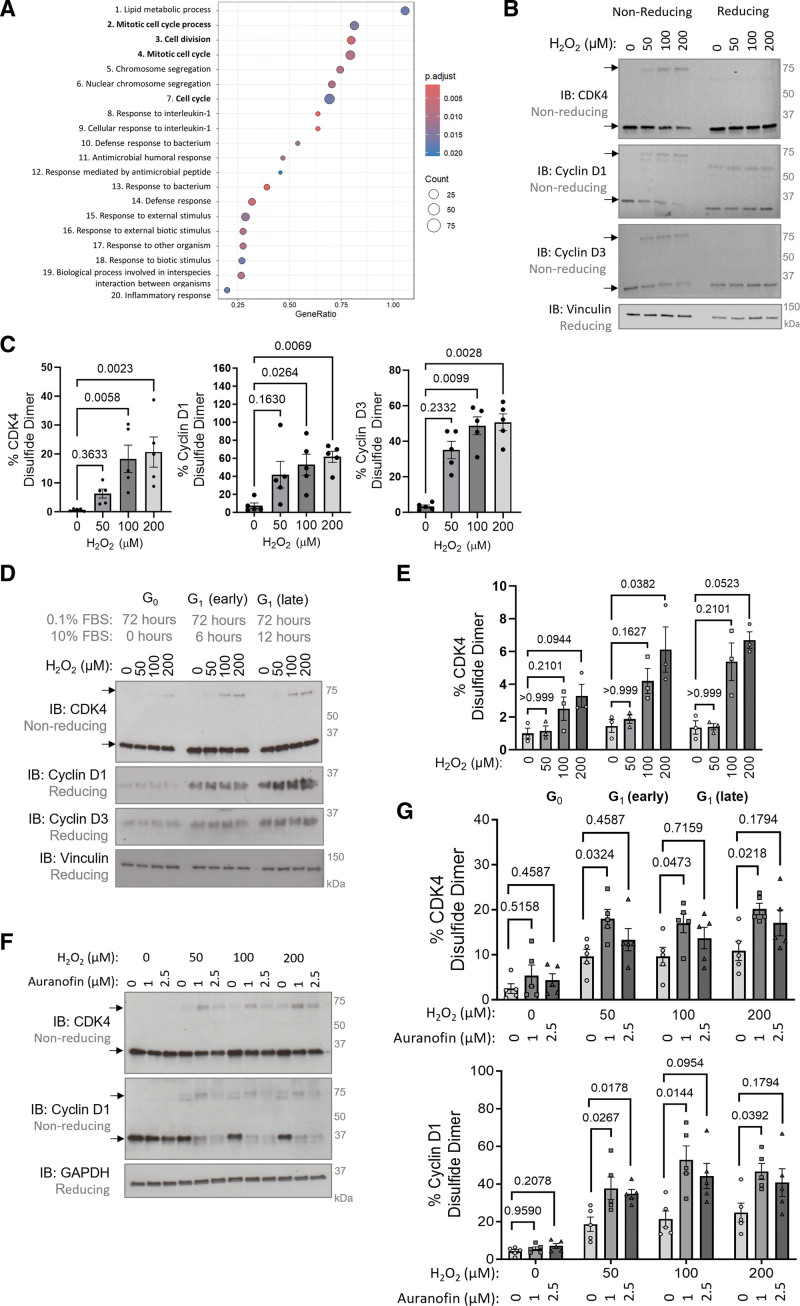
**Cyclin D-CDK4 (cyclin-dependent kinase 4) forms an inducible intermolecular disulfide dimer. A**, An Affymetrix GeneChip microarray with gene enrichment pathway analysis of approximately the top 1000 genes of defined biological function with an altered expression between mice exposed to chronic hypoxia (10% oxygen) or normoxia (21% oxygen) for 3 days, n=6 mice per group. The top 20 gene-enriched pathways are shown based on pathway analysis performed in R studio using the gseGO function of the clusterProfiler package. **B**, CDK4, cyclin D1, and cyclin D3 form oxidant-induced intermolecular disulfide bonds. Monomeric and disulfide dimeric (indicated by black arrows) CDK4, cyclin D1, or cyclin D3 were detected by nonreducing immunoblotting in human pulmonary arterial smooth muscle cells (HPASMCs) after treatment with H_2_O_2_. Vinculin was used as a loading control. **C**, The percentage of CDK4, cyclin D1, and cyclin D3 observed as a disulfide dimer in response to H_2_O_2_ treatment. As the data sample size with an n<6 cannot be reliably tested for normality, *P* values are calculated using a nonparametric Kruskal-Wallis test followed by Dunn multiple comparisons to compare H_2_O_2_-induced disulfide formation with 0 µmol/L control (n=5 independent experiments); the results are shown as means±SEM. **D**, Cyclin D expression is higher in G1 phase cells than quiescent cells, causing an increase in disulfide cyclin D-CDK4. Monomeric and disulfide dimeric CDK4 were detected by nonreducing immunoblotting of HPASMCs synchronized into the G0/1 phase by serum starvation (0.1% FBS) for 72 hours, followed by stimulation with 10% FBS for 6 or 12 hours. HPASMCs were then treated with H_2_O_2_ for 15 minutes. Cyclin D1, cyclin D3, and vinculin (loading control) were detected by immunoblotting under reducing conditions. **E**, Quantification of the percentage of CDK4 observed as a disulfide dimer. As the data sample size with an n<6 cannot be reliably tested for normality, *P* values are calculated using a nonparametric Kruskal-Wallis test followed by Dunn multiple comparisons to compare H_2_O_2_-induced disulfide formation with 0 µmol/L control within cell cycle phase (n=3 independent experiments); the results are shown as means±SEM. **F**, Auranofin potentiates accumulation of the oxidant-induced cyclin D1-CDK4 disulfide bond. Monomeric and disulfide dimeric (indicated by black arrows) CDK4 and cyclin D1 were detected by nonreducing immunoblotting in HPASMCs after pretreatment with the thioredoxin reductase inhibitor, auranofin, for 25 minutes followed by the addition of H_2_O_2_ for 15 minutes. GAPDH was used as a loading control. **G**, The proportion of CDK4 and cyclin D1 observed as a disulfide dimer after treatment with auranofin and H_2_O_2_. *P* values are calculated using a nonparametric Kruskal-Wallis test followed by Dunn multiple comparisons to compare disulfide formation in response to auranofin treatment within 0, 50, 100 or 200 µM H_2_O_2_-treatment group (n=5 independent experiments); the results are shown as means±SEM.

By employing an unbiased Affymetrix microarray, pathway analysis of differentially expressed genes was performed using the lungs of mice exposed to chronic hypoxia for 3 days. In line with the previously reported overactivation of CDKs in PH,^36^ numerous cell cycle pathways were enriched in response to hypoxia (Figure 1A), suggesting cell cycle dysregulation occurs early in disease pathogenesis. Chronic hypoxia is a time when ROS production is altered,^24^ which may influence protein redox modifications. Therefore, it was intuitive to hypothesize that the key cell cycle regulators, CDKs, are redox-regulated, which may alter their activity in disease. Initial observations of a disulfide bond in cyclin D1-CDK4 and cyclin D3-CDK4 were made in nonsynchronized HPASMCs in response to H_2_O_2_ treatment, where formation of a higher molecular weight complex at ≈75 kDa was observed, which is indicative of an intermolecular disulfide bond (Figure 1B and 1C). This observation was reproducible in human pulmonary arterial endothelial cells treated with H_2_O_2_ (Figure S1A and S1B), and in HPASMCs exposed to UV-induced oxidation (Figure S1C). Additional evidence for CDK4 cysteine oxidation was obtained using the PEG-switch assay,^48^ which revealed a higher molecular weight band in response to H_2_O_2_ that represents pegylated CDK4, confirming the presence of a reactive cysteine residue (Figure S1D).

Based on structural considerations^49,50^ including the heterodimeric nature of the cyclin D-CDK4 protein complex and its combined molecular weight, the disulfide bond was hypothesized to form between cyclin D and CDK4. However, there was a potential discrepancy in the proportion of each protein that formed the disulfide, with ≈60% of cyclin D and ≈20% of CDK4 protein forming the disulfide (Figure 1C). This can be explained by cyclin D being 10-fold lower in abundance than CDK4 (Figure S2A), meaning it limits the formation of a protein heterodimer and thus the amount of intermolecular disulfide that can form. Abundance of cyclin D is known to be periodic throughout the cell cycle,^44^ and as anticipated, G1 phase synchronization of HPASMCs elevated cyclin D expression causing a notable increase in CDK4 disulfide formation, in contrast to G0 phase (Figure 1D and 1E). Similarly, overexpression of cyclin D1 or cyclin D3 markedly increased the proportion of CDK4 that formed a disulfide dimer, while the opposite was observed in response to cyclin D knockdown using siRNA (Figure S2B and S2C).

Next, we sought to identify whether the cyclin D-CDK4 disulfide bond was endogenously reduced by intracellular antioxidant systems, such as NADPH-dependent thioredoxin/thioredoxin reductase.^51^ Treatment of HPASMCs with the thioredoxin reductase inhibitor, 1 μM auranofin, induced the accumulation of disulfide cyclin D-CDK4 (Figure S3A) and potentiated H_2_O_2_-induced oxidation of cyclin D-CDK4 (Figure 1F and 1G). Moreover, endogenous reduction of the cyclin D-CDK4 disulfide is shown by a time-dependent decrease in detection after H_2_O_2_ treatment (Figure S3B).

### A Heterodimeric Disulfide Bond Forms Between CDK4 C135 and Cyclin D1 C7/8

**Figure 2.**
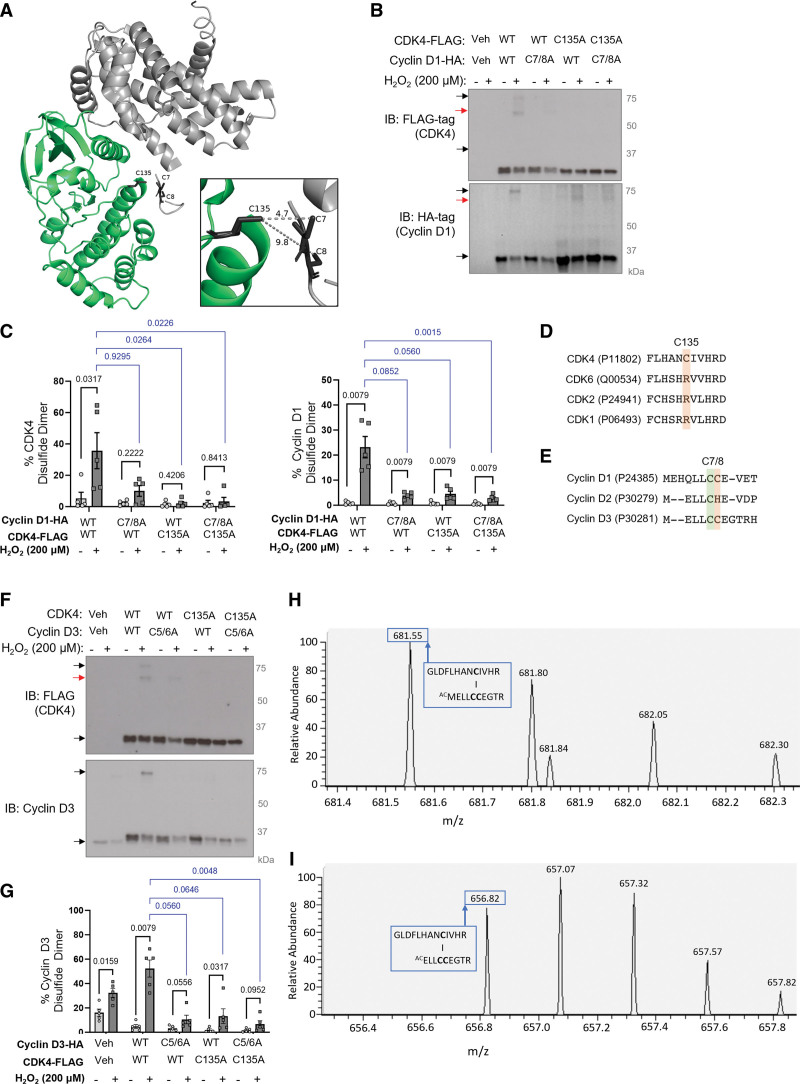
**The intermolecular disulfide bond forms between CDK4 (cyclin-dependent kinase 4) C135 and cyclin D1 C7/8. A**, The cyclin D1-CDK4 crystal structure (PDB 2W96) showing the locations of CDK4 C135 and cyclin D1 C7/8 in black. The distance (Å) between cysteine residues was measured using PyMOL. The cyclin D1 protein structure and CDK4 protein structure are shown in gray and green, respectively. **B**, C7/8A cyclin D1 and C135A CDK4 are redox-dead. Immunoblots show disulfide dimeric cyclin D1 and CDK4 (indicated by black arrows) in human pulmonary arterial smooth muscle cells (HPASMCs) overexpressed with wild-type (WT) or C135A CDK4-FLAG and WT or C7/8A cyclin D1-HA and treated with H_2_O_2_. Cyclin D1 (HA-tag) and CDK4 (FLAG-tag) were detected by nonreducing immunoblotting. Red arrows indicate additional artificial bands. **C**, The percentage of cyclin D1 and CDK4 observed as a disulfide dimer in overexpressed HPASMCs. As the data sample size with an n<6 cannot be reliably tested for normality, *P* values are calculated using unpaired 2-tailed nonparametric Mann-Whitney *U* test to compare between vehicle and H_2_O_2_ treatment for each mutant; nonparametric Kruskal-Wallis test followed by Dunn multiple comparisons was used to compare disulfide formation in response to 200 µM H_2_O_2_ treatment between each mutant and WT (n=5 independent experiments); the results are shown as means±SEM. **D**, BLAST alignments show CDK4 C135 is not conserved between cell cycle CDK proteins. **E**, BLAST alignments show cyclin D1 C7/8 is conserved between cyclin D subtypes. All sequences were obtained from the UniProt database with identification codes presented in brackets. **F**, C5/6A cyclin D3 and C135A CDK4 are redox-dead. Disulfide dimeric cyclin D3 and CDK4 (indicated by black arrows) in HPASMCs overexpressed with WT or C135A CDK4-FLAG and WT or C5/6A cyclin D3-HA. Cells were treated with H_2_O_2_ for 15 minutes. Cyclin D3 and CDK4 (FLAG-tag) were detected by nonreducing immunoblotting. Red arrows indicate additional artificial bands. **G**, The percentage of cyclin D3 and CDK4 observed as a disulfide dimer in overexpressed HPASMCs. *P* values are calculated using unpaired 2-tailed nonparametric Mann-Whitney *U* test to compare between H_2_O_2_ treatment and vehicle for each mutant; nonparametric Kruskal-Wallis test followed by Dunn multiple comparisons was used to compare disulfide formation in response to 200 µM H_2_O_2_ treatment between each mutant and WT (n=5 independent experiments); the results are shown as means±SEM. **H** and **I**, Analysis of an extracted ion chromatogram from an LC-MS/MS precursor ion survey scan identified an ion of (**H**) *m/z* 681.55^4+^ and (**I**) *m/z* 656.82^4+^, which correspond with the peptide ^127^GLDFLHANCIVHR^139^ in CDK4 bound through a disulfide bond to the peptide ^1^MELLCCEGTR^10^ in cyclin D3, as shown in the red boxes. The cyclin D3 peptide was identified with dioxidation of the free cysteine residue, and acetylation of the N-terminal, either with the N-terminal methionine (**H**) present, or (**I**) absent.

To identify which cysteine residues form a disulfide bond, the crystal structure of cyclin D1-CDK4 (PDB 2W96^49^) was explored. We found that CDK4 C135 is in close proximity to cyclin D1 C7 and C8, with thiol distances of 4.7 Å and 9.8 Å, respectively (Figure 2A). Using a cysteine oxidation prediction algorithm,^52^ CDK4 C135 was predicted to have a p*K*_a_ of 6.7, making it likely that this residue exists in a deprotonated state in cells, and is susceptible to oxidation to form a sulfenic acid intermediate, followed by a disulfide bond. Cysteine to alanine mutants were generated to identify whether these particular cysteine residues form the cyclin D-CDK4 disulfide bond. Overexpression of a cyclin D1-HA C7/8A double mutant and CDK4-FLAG C135A mutant inhibited formation of the disulfide bond (Figure 2B and 2C), while CDK4 C78A and CDK4 C202A mutants remained redox-active (data not shown), confirming the location of the disulfide bond. Interestingly, the presence of either cyclin D1 C7 or C8 was sufficient to permit disulfide formation, likely due to their vicinal proximity, meaning only the C7/8A double mutant was strikingly redox-dead (Figure S4). Of note, an additional complex at 60 to 70 kDa, as indicated by a red arrow (Figure 2B), was also detected in nonreducing immunoblots. As these additional bands are a lower molecular weight than the cyclin D-CDK4 disulfide dimer and are not observed endogenously, they do not represent the cyclin D-CDK4 disulfide bond and are likely to be homodimers that reflect a limitation of protein overexpression. While CDK4 C135 is highly conserved in mammals, it is not present in other cell cycle CDKs (Figure 2D; Figure S5A), potentially representing a mechanism of kinase regulation that is unique to CDK4. On the other hand, cyclin D1 C7 and C8 are highly conserved in both mammals and between cyclin D subtypes (Figure 2E; Figure S5B). Generation of a cyclin D3 C5/6A double mutant, which is equivalent to cyclin D1 C7/8, revealed a similar redox-dead phenotype in HPASMCs (Figure 2F and 2G).

To confirm that disulfide cyclin D-CDK4 is indeed observed at ≈75 kDa, LC-MS/MS was employed, first to identify the proteins present at this molecular weight in a sample of coimmunoprecipitated CDK4 from HeLa cells treated with H_2_O_2_. Both CDK4 (MW 34 kDa) and cyclin D3 (MW 33 kDa) were enriched at ≈75 kDa under oxidizing conditions compared with reducing conditions, supporting the presence of a disulfide bond that results in electrophoretic separation at their combined molecular weight (Figure S6A). High-sequence coverage was obtained for both proteins of interest, with all CDK4 cysteine residues identified (Figure S6B and S6C). Conclusive evidence of a disulfide bond between cyclin D3 and CDK4 was obtained in a parallel sample, which was enzymatically digested in the absence of reduction and alkylation steps to preserve the intact disulfide. Here, interrogation of the extracted ion chromatogram revealed evidence of 2 peptides in the oxidized H_2_O_2_ treated samples, of *m/z* 681.55^4+^ and *m/z* 656.82^4+^, which correspond to the predicted *m/z* of a peptide containing CDK4 C135 coupled to cyclin D3 C5/6 through a disulfide bond, either with or without the N-terminal methionine present in cyclin D3 (Figure 2H and 2I; Figure S7). As the peptide of interest in cyclin D3 contains 2 cysteine residues, it was predicted that 1 cysteine would be bound through a disulfide, while the other would be free and thus susceptible to dioxidation or trioxidation. The extracted ion chromatogram of the precursor ion confirmed the presence of this combined peptide, but it was not possible to identify whether cyclin D3 C5 or C6 preferentially forms the disulfide bond with CDK4 C135 (Figure 2H and 2I). While the combined cyclin D3-CDK4 peptide was observed under oxidizing conditions, no evidence was obtained under reduced conditions, corroborating an oxidant-induced heterodimeric disulfide bond formation between CDK4 C135 and cyclin D3 C5/6.

### Oxidation of Cyclin D-CDK4 Inhibits Kinase Activity and Induces G1 Phase Cell Cycle Arrest

**Figure 3.**
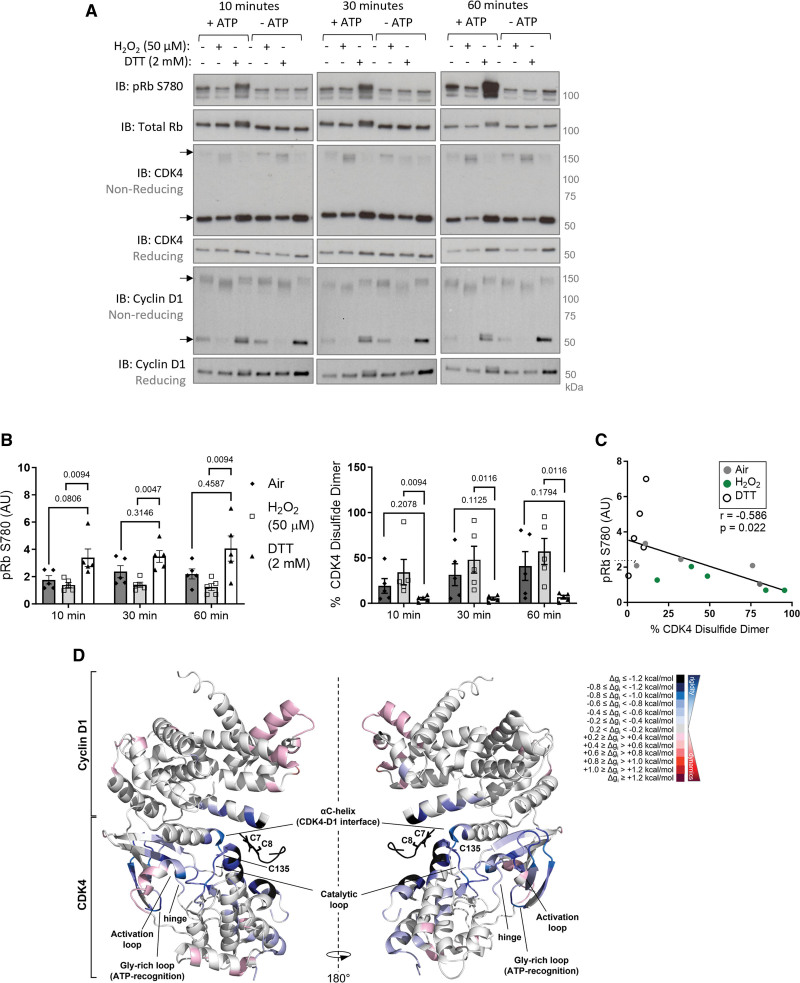
**Oxidation of C135 inhibits cyclin D1-CDK4 (cyclin-dependent kinase 4) kinase activity. A**, An in vitro kinase activity assay showing oxidation inhibits kinase activity of recombinant cyclin D1-CDK4 toward recombinant Rb substrate. Cyclin D1-CDK4 protein was oxidized by either air or H_2_O_2_ or reduced by DTT. Phosphorylation of Rb protein substrate was measured after incubation for 10, 30, or 60 minutes. pRb S780, total Rb, CDK4, and cyclin D1 were detected by immunoblotting under reducing conditions. Disulfide CDK4 and disulfide cyclin D1 was detected by immunoblotting under nonreducing conditions. **B**, Quantification of the proportion of CDK4 observed as a disulfide dimer, and pRb S780 normalized to control (untreated with no ATP). As the data sample size with an n<6 cannot be reliably tested for normality, *P* values are calculated using a nonparametric Kruskal-Wallis test followed by Dunn multiple comparisons to compare Air or H_2_O_2_ treatment to DTT for 10, 30, or 60-minute time points (n=5 independent experiments); the results are shown as means±SEM. **C**, Negative correlation between pRb S780 and CDK4 disulfide formation at the 60-minute time point. Analysis was performed using Simple Linear Regression model showing the line of best fit and Pearson correlation coefficient (r; n=5 independent experiments). **D**, AlloSigMA analysis of probing of CDK4 C135 and cyclin D1 C7/8 shows extensive rigidification of CDK4. The panel shows a cartoon model of the cyclin D1-CDK4 complex (PDB 2W96). The cysteine residues involved in the formation of the intermolecular disulfide bond, and the key secondary structure elements of interest are labelled. The per-residue allosteric free energy (Δg_i_) values obtained in the AlloSigMA calculation are mapped onto the structure: the darker the shade of red or blue, the greater the destabilization or gain in rigidity, respectively. DTT indicates dithiothreitol; and Rb, retinoblastoma.

To identify whether oxidation of cyclin D-CDK4 regulates protein function, an in vitro kinase activity assay was employed. Consistent with previous reports,^53,54^ the cyclin D1-CDK4 holoenzyme phosphorylates a full-length Rb protein substrate under reducing conditions (Figure 3A and 3B). However, we found that incubation under oxidizing conditions, which is when the disulfide bond forms, inhibits kinase activity resulting in negligible levels of Rb phosphorylation (Figure 3A and 3B; Figure S8A). As a result, an inverse relationship was observed between disulfide CDK4 and Rb phosphorylation (Figure 3C; Figure S8B), suggesting the presence of a redox-switch in cyclin D-CDK4.

To rationalize the effects of the disulfide bond between CDK4 C135 and cyclin D1 C7/8 on kinase activity, we performed in silico AlloSigMA experiments. This structural modeling tool analyses allosteric communications following a molecular perturbation, namely an intermolecular disulfide bond in this case, by calculating per-residue allosteric free energy variations (Δg_i_). While an increase in Δg_i_ entails augmented dynamics, a decrease in Δg_i_ indicates rigidification of residues in the cyclin D1-CDK4 complex.^55^ To evaluate the effects of the intermolecular disulfide bond on protein structure, the analysis was performed by probing CDK4 C135 and cyclin D1 C7/8. Remarkably, we found that CDK4 undergoes significant rigidification in the hinge, αC-helix, and catalytic loop (Figure 3D; Figure S9A). Our data indicate negligible effects on cyclin D1, upon formation of the disulfide bond, suggesting that the allosteric communication is specific to the kinase (Figure S9A). Notably, when CDK4 C135 was perturbed in isolation, rigidification was still observed in the hinge, and the catalytic and activation loops, corroborating the finding that oxidation of this residue alters kinase activity (Figure S9B). In contrast, perturbation of either CDK4 C78 or C215 revealed only a minor rigidification of the activation loop but failed to allosterically communicate with the hinge or catalytic loop, suggesting that oxidation of these residues does not affect kinase activity (Figure S9B).

**Figure 4.**
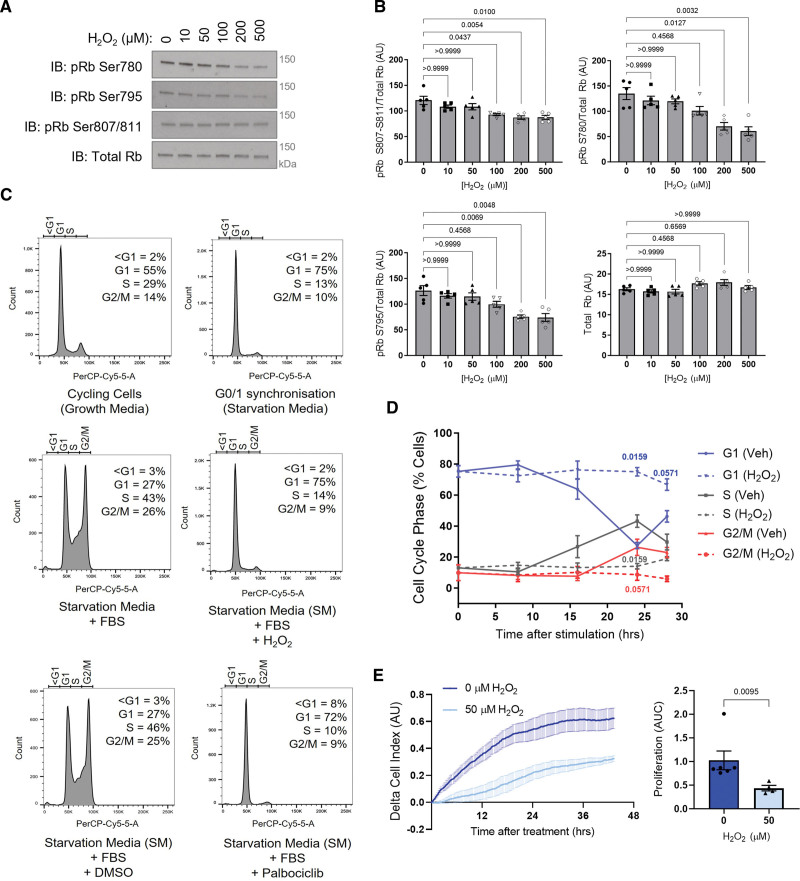
**Oxidation decreases cyclin D-CDK4 (cyclin-dependent kinase) substrate phosphorylation and induces G1 phase cell cycle arrest. A**, Oxidation of human pulmonary arterial smooth muscle cells (HPASMCs), induced by acute H_2_O_2_ treatment, decreased. Rb (retinoblastoma) phosphorylation at S780, S795, or S807-811, and total Rb were detected by immunoblotting under reducing conditions. **B**, Quantification of Rb phosphorylation, normalized to total Rb. As the data sample size with an n<6 cannot be reliably tested for normality, *P* values are calculated using a nonparametric Kruskal-Wallis test followed by Dunn post hoc multiple comparisons to compare Rb phosphorylation in response to H_2_O_2_ with the 0 µmol/L control (n=5 independent experiments); the results are shown as means±SEM. **C**, Histograms show cell cycle analysis using flow cytometry of propidium iodide-stained HPASMCs. Continuously cycling cells were maintained in DMEM growth medium (10% FBS; upper left), while G0/1 phase synchronized cells were maintained in starvation media (0.1% FBS) for 40 hours (upper right). Synchronized cells were stimulated to enter the cell cycle with 10% FBS alone for 24 hours (middle left), FBS plus 50 µmol/L H_2_O_2_ for 24 hours (middle right), FBS plus DMSO vehicle (bottom left), or FBS plus 10 µmol/L palbociclib for 24 hours (bottom right). The proportion of cells in each phase of the cell cycle at this time point was analyzed by the Watson Pragmatic cell cycle model and an average from 4 to 5 independent experiments is provided. **D**, H_2_O_2_ causes G1 phase cell cycle arrest. Graph shows the proportion of synchronized cells in each cell cycle phase with time after stimulation with 10% FBS alone (solid lines) or 10% FBS plus 50 µmol/L H_2_O_2_ (dashed lines). *P* values are calculated using unpaired 2-tailed nonparametric Mann-Whitney *U* test to compare between vehicle and H_2_O_2_ treatment in cell cycle phases at each time point (0 and 8 hours, n=5 experimental sample per group; 16, 24, or 28 hours, n=4 experimental samples per group); the results are shown as means±SEM. **E**, Proliferation of HPASMCs treated with 50 µmol/L H_2_O_2_ or vehicle. Cells were seeded in xCELLigence real-time cell analysis (RTCA) E-plates at 3×10^4^ cells/well in starvation media (0.1% FBS). After 40 hours, cells were stimulated with 10% FBS with or without H_2_O_2_. Proliferation rate of HPASMCs after treatment was quantified using area under the curve. As the data sample size with an n<6 cannot be reliably tested for normality, the *P* value is calculated using an unpaired 2-tailed nonparametric Mann-Whitney *U* test to compare between area under the curve for 0 and 50 µmol/L (n=4 independent experiments/ biological replicates per group, including n=2 performed in technical replicates for 0 µmol/L, altogether comprising n=6 replicates for 0 µmol/L and n=4 replicates for 50 µmol/L); the results are shown as means±SEM.

Next, to support the in vitro kinase activity data, HPASMCs were treated with H_2_O_2_, which induced a dose-dependent decrease in Rb phosphorylation (Figure 4A and 4B), in line with CDK4 inhibition. Importantly, a decrease in Rb phosphorylation was observed at 3 sites, including S780 which is a CDK4/6 specific site.^56^ Moreover, using propidium iodide staining to detect cell cycle stages, H_2_O_2_ was found to induce a G1 phase cell cycle arrest in HPASMCs, making the cells insensitive to serum-stimulation (Figure 4C and 4D; Figure S10), while cells stimulated with serum in the absence of oxidation progressed through the cell cycle (Figure 4C and 4D). This observation was also consistent with data obtained when measuring the proliferation rate of HPASMCs using real-time cell analysis (Figure 4E). H_2_O_2_ treatment caused a decrease in proliferation rate compared with cells stimulated with serum alone (Figure 4E). Similar observations were made in human pulmonary arterial endothelial cells, where H_2_O_2_ treatment inhibited proliferation in a manner that was comparable to the CDK4/6 inhibitor (Figure S11A and S11B).

Given the reported similarities between PH and cancer,^4^ and the efficacy of CDK4/6 inhibitors in the treatment of breast cancer, we sought to identify whether a similar oxidant-induced inhibition was observed in the proliferation of MCF7 breast epithelial cells. As anticipated, treatment with H_2_O_2_ or the pharmacological thioredoxin reductase inhibitor, auranofin, decreased the proliferation rate of MCF7 cells to a similar extent as the classical CDK4/6 inhibitor, palbociclib (Figure S11C and S11D).

### CDK4 C135 Is Critical for Optimal Cyclin D-CDK4 Kinase Activity and Cell Proliferation

**Figure 5.**
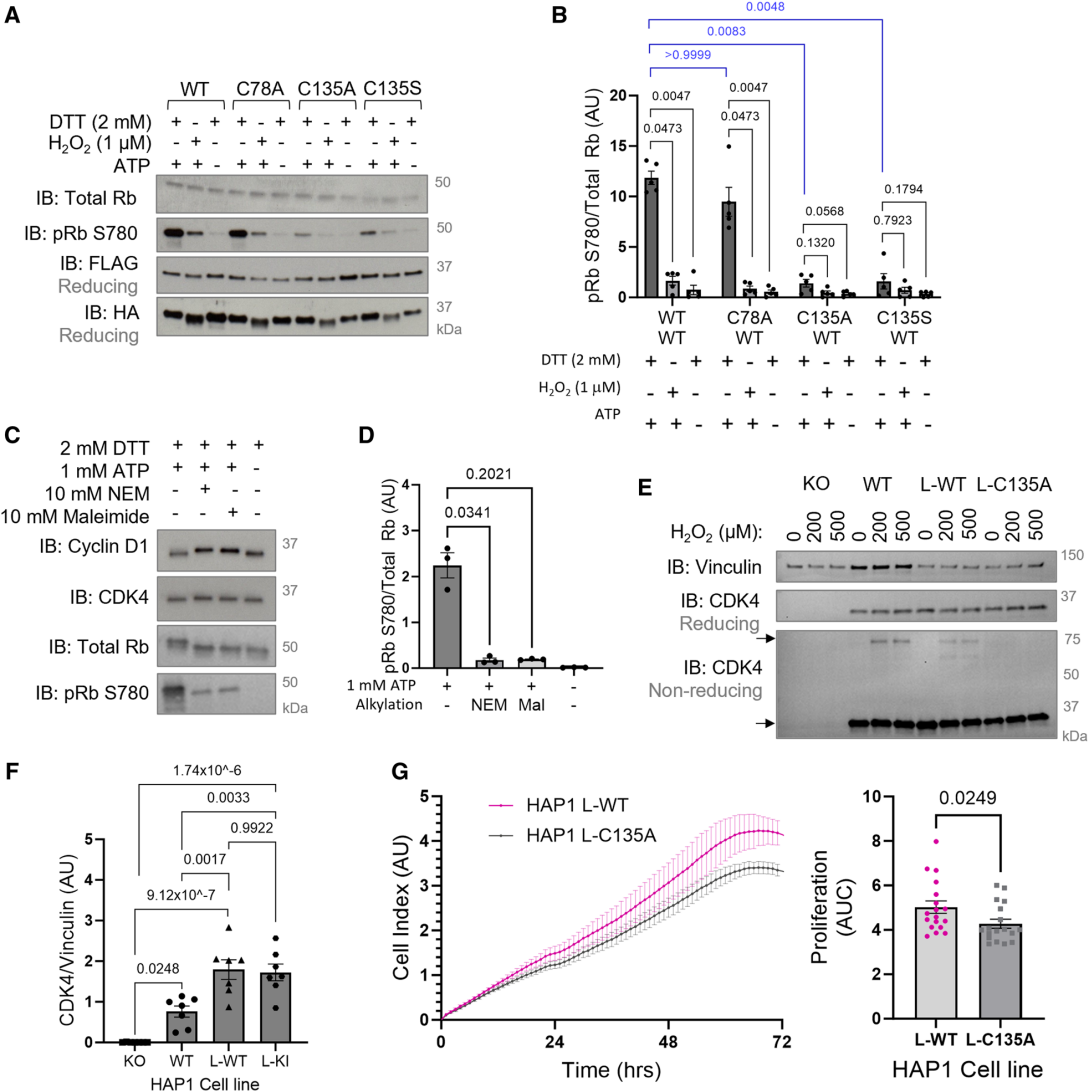
**C135A CDK4 (cyclin-dependent kinase) is kinase-impaired causing a decreased proliferation rate of cells. A**, An in vitro kinase activity assay of coimmunoprecipitated WT or mutant cyclin D1-CDK4 shows C135A/S CDK4 is kinase-impaired. WT, C135A, C135S, or C78A CDK4-FLAG, and WT cyclin D1-HA were overexpressed and coimmunoprecipitated from HAP1 (near-haploid human cell line) CDK4 KO cells. Protein was incubated with 2 mmol/L DTT, 1 µmol/L H_2_O_2_ or vehicle (H_2_O) to reduce or oxidize, respectively. Rb (retinoblastoma) phosphorylation was measured by immunoblotting after 60-minute incubation with recombinant Rb protein substrate. **B**, Quantification of pRb S780 normalized to total Rb. As the data sample size with an n<6 cannot be reliably tested for normality, *P* values are calculated using a nonparametric Kruskal-Wallis test followed by Dunn post hoc multiple comparisons to compare H_2_O_2_ or no ATP-treated conditions to DTT+ATP control within each mutant group, and DTT-treated conditions between the WT, C78A, and C135A/S mutants (n=5 independent experiments); the results are shown as means±SEM. **C**, An in vitro kinase activity assay shows thiol alkylation inhibits cyclin D1-CDK4 kinase activity toward Rb. Cyclin D1-CDK4 was reduced by DTT and incubated with 10 mmol/L *N*-ethylmaleimide (NEM) or 10 mmol/L maleimide for 2 hours. Phosphorylation of recombinant Rb substrate was measured by immunoblotting after incubation for 60 minutes. CDK4, cyclin D1, total Rb, and pRb S780 were detected by immunoblotting under reducing conditions. **D**, Quantification of pRb S780 normalized to total Rb after incubation with or without NEM or maleimide. As the data sample size with an n<6 cannot be reliably tested for normality, *P* values are calculated using a nonparametric Kruskal-Wallis test followed by Dunn post hoc multiple comparisons to compare Rb phosphorylation in response to alkylating agents with no alkylating agent+ATP control (n=3 independent experiments); the results are shown as means±SEM. **E**, Reducing immunoblots showing CDK4 expression and nonreducing immunoblots showing monomeric and disulfide dimeric CDK4 in CDK4 KO HAP1 cells, WT HAP1 cells, and HAP1 lentiviral stable cell lines expressing WT (L-WT) or C135A (L-C135A) CDK4. Cells were treated with H_2_O_2_ for 15 minutes. Vinculin was used as a loading control. **F**, Quantification of CDK4 expression normalized to vinculin. The data passed the normality test performed by the Shapiro-Wilk test. *P* value is calculated using 1-way ANOVA followed by Tukey post hoc multiple comparisons to compare between the groups (n=7 independent experiments per group); the results are shown as means±SEM. **G**, A decreased proliferation rate of L-C135A HAP1 cells compared with L-WT HAP1 cells. Proliferation of L-WT and L-C135A HAP1 cells was measured by electrical impedance (cell index) for 72 hours. Cells were seeded in xCELLigence real-time cell analysis (RTCA) E-plates at 2×10^4^ cells/well. Proliferation rate was quantified by area under the curve (AUC) of L-WT and L-C135A cells measured by RTCA. As the data sample size with an n<6 cannot be reliably tested for normality, *P* value is calculated using unpaired 2-tailed nonparametric Mann-Whitney *U* test to compare AUC values between L-WT and L-C135A (n=5 independent biological replicates per group, in technical quadruplicates for the first n=4 and duplicates for the last n=1, altogether comprising 18 replicates); the results are shown as means±SEM. DTT indicates dithiothreitol.

The kinase-inhibitory nature of the disulfide bond was reproducible in WT cyclin D1-CDK4 protein that was overexpressed and coimmunoprecipitated from HPASMCs or HAP1 CDK4 knockout cells (Figure 5A and 5B; Figure S12). While activity was observed under reducing conditions in WT cyclin D1-CDK4, remarkably, the C135A or C135S cyclin D1-CDK4 mutant displayed a kinase-impaired phenotype, as shown by a decrease in Rb phosphorylation (Figure 5A and 5B). To ensure this finding was specific to CDK4 C135, an alternative cysteine residue, C78, which is located in the N-lobe of CDK4, was mutated. C78A cyclin D1-CDK4 displayed comparable activity to WT cyclin D1-CDK4 under reducing conditions and retained its sensitivity to oxidant-induced inhibition (Figure 5A and 5B). These data, together with in silico modeling using AlloSigMA (Figure S9), suggest that CDK4 C135 plays a critical role in cyclin D-CDK4 kinase activity, possibly through allosteric communications that permit structural rearrangement of the kinase into the active conformation. Interestingly, CDK4 kinase activity was also inhibited by incubation with the alkylating agent *N*-ethylmaleimide or markedly reduced by the alkylating agent maleimide (Figure 5C and 5D), which both adduct to cysteine thiols, supporting the role of a cysteine residue, C135, in being critical for optimal kinase activity.

A stable cell line expressing WT or redox-dead C135A CDK4 was generated from CDK4 KO HAP1 cells using lentiviral transduction. The expression of CDK4 was comparable between L-WT or L-C135A HAP1 cells, and as expected, the cyclin D-CDK4 disulfide was absent in the L-C135A HAP1 cells (Figure 5E and 5F; Figure S13). It was anticipated that this redox-dead stable cell line would also be kinase-impaired, meaning cells with the CDK4 C135A mutation could not be used to show resistance to oxidant-induced cell cycle arrest. For this reason, the L-C135A HAP1 cells were used to show the critical role of C135 in CDK4 activity and proliferation, providing a surrogate for C135 oxidation. Mutation of CDK4 C135 caused a considerably small but reproducible and significant decrease in the proliferation rate of HAP1 cells, supporting the role of C135 as a critical cysteine residue that regulates CDK4 function and cell proliferation (Figure 5G).

**Figure 6.**
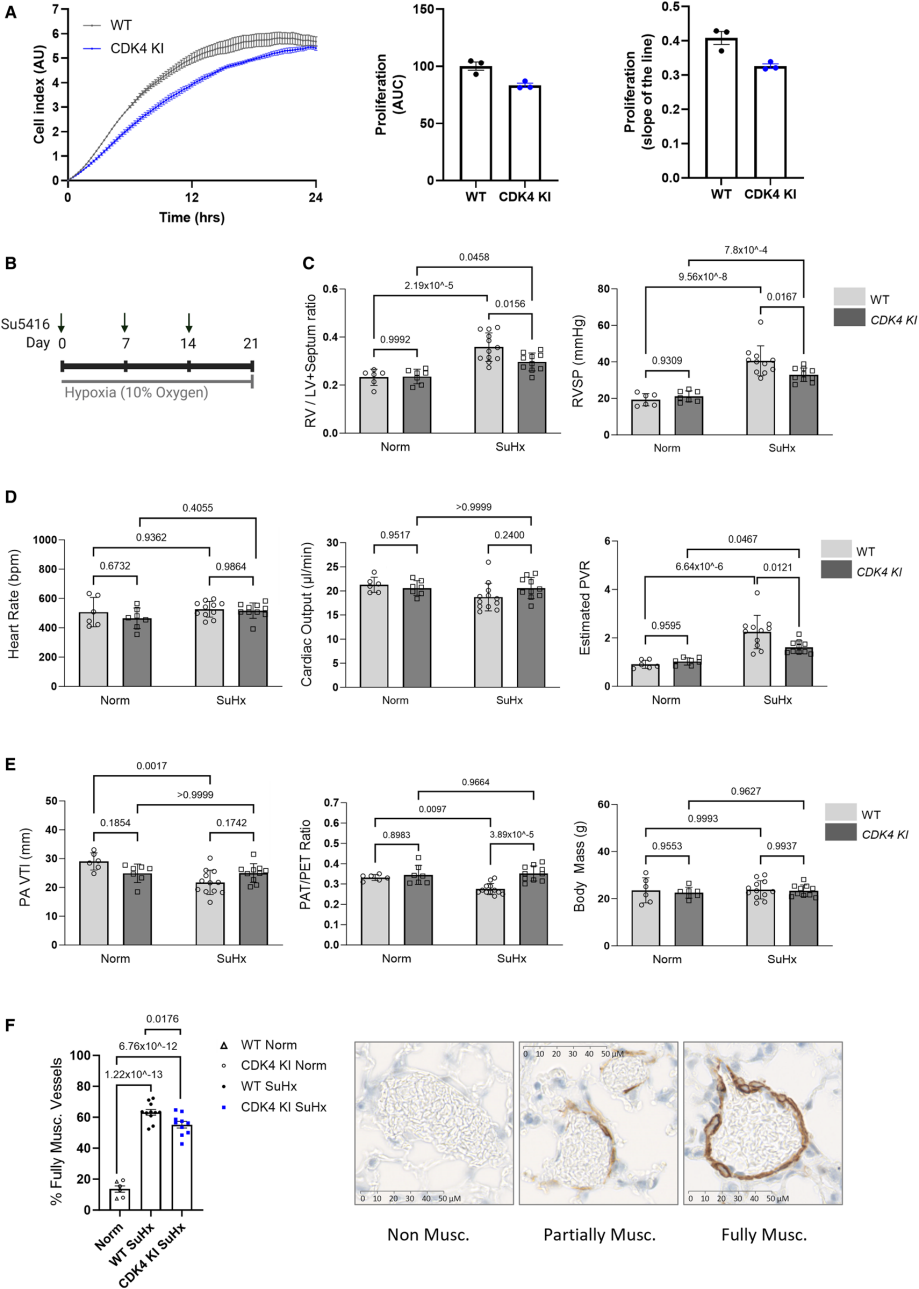
**Redox-dead C135A CDK4 (cyclin-dependent kinase) KI mice develop a less severe disease phenotype in Sugen/hypoxia experimental models of pulmonary hypertension (PH). A**, Mouse lung endothelial cells (MLECs) isolated from C135A CDK4 KI mice demonstrate a decreased proliferation rate compared with MLECs isolated from wild-type (WT) mice. Proliferation of MLECs was measured by electrical impedance (cell index) for 24 hours. Cells were seeded in xCELLigence gelatin and fibronectin-coated real-time cell analysis (RTCA) E-plates at 5×10^3^ cells/well. Proliferation rate was analyzed by area under the curve of MLECs, and the slope is calculated from the linear portion of the graph (first 14 hours). Two WT or 2 CDK4 KI mouse lungs were pooled for one cell isolation procedure per genotype, and the proliferation experiment was run in technical triplicate. **B**, The Sugen (SU5146)/hypoxia mouse model of PH was induced by exposure to hypoxia (10% O_2_) for 3 weeks along with weekly subcutaneous injections of 20 mg/kg Sugen (Su5416). Control mice were maintained in normoxia (Norm, 21% oxygen). Male and female mice were used. **C**, Right ventricular (RV) hypertrophy (RV/LV+septum ratio) and RVSP (mm Hg), (**D**) heart rate (beats per minute), cardiac output (μL/minute) and estimated pulmonary vascular resistance (PVR), (**E**) pulmonary flow velocity time integral (PA VTI), pulmonary flow acceleration time (PAT)/pulmonary ejection time (PET) ratio and body mass were measured in both sexes of WT or KI mice to assess the severity of PH. **C** through **E**, An assessment of the data normality was performed by the Shapiro-Wilk test; all data parameters presented in **C**, **D**, and **E** of this figure passed the normality test. As the data passed the test for normality, *P* values were calculated using a parametric test 2-way ANOVA followed by Tukey multiple comparisons tests to compare between all groups (WT Norm, n=6; CDK4 KI Norm, n=7; WT SuHx, n=12; CDK4 KI SuHx, n=10), and the results are shown as means±SEM. **F**, Muscularization of small pulmonary arteries (10–100 µm) was measured using alpha smooth muscle actin (α-SMA) immunohistochemical staining of mouse lung sections after induction of the Sugen/hypoxia pulmonary hypertension model. The data passed the normality test performed by the Shapiro-Wilk test. *P* values were calculated using a 1-way ANOVA followed by Tukey multiple comparisons test to compare between all groups (Norm, n=6; WT SuHx, n=11; CDK4 KI SuHx, n=10); the results are shown as means±SEM. WT and CDK4 KI mice from normoxic control groups were combined into 1 group.

A novel redox-dead constitutive C135A CDK4 knock-in (KI) mouse was next generated (Figure S14) and employed to definitively establish the importance of C135 in pulmonary vascular cell proliferation. As anticipated and consistent with C135A CDK4 being kinase-impaired, both mouse pulmonary arterial smooth muscle cells (Figure S15A and S15B) and mouse lung endothelial cells (Figure 6A; Figure S15C) isolated from C135A CDK4 KI mice demonstrated a pronounced decrease in the proliferation rate compared with cells isolated from WT littermates. While these data further support the role of C135 CDK4 as a critical cysteine residue that regulates cell proliferation, it was intuitive to test the role of C135A mutation in pulmonary vascular disease progression. For this reason, adult WT or KI male or female mice were subjected to a widely used model of experimental PH induced by weekly subcutaneous injections of Sugen (SU5416) in combination with 3 weeks of hypoxic exposure (Figure 6B). Interestingly, redox-dead C135A CDK4 KI mice developed a less severe PH phenotype in the Sugen/hypoxia experimental PH model, which was manifested by an attenuated right ventricular (RV) systolic pressure (RVSP), RV hypertrophy and estimated pulmonary vascular resistance, compared with WT littermates (Figure 6C and 6D; Figure S16A through S16C). The protection in KI mice after Sugen/hypoxia was further evidenced by an attenuated decline in pulmonary arterial blood flow indexes (Figure 6E), reduced pulmonary vascular muscularization (Figure 6F), and notably improved blood oxygen saturation (Figure S17A). There was no difference in nonfasting blood glucose, sodium, potassium or calcium concentration, hematocrit, hemoglobin, or blood pH between WT and KI mice after Sugen/hypoxia, assessed by blood analyzer (Figure S17B through S17E). Altogether, the data are consistent with the role of C135 as a critical cysteine residue that regulates CDK4 function and pulmonary vascular cell proliferation in vitro and in vivo.

### Therapeutic Implications of the Cyclin D-CDK4 Disulfide In Vivo

**Figure 7.**
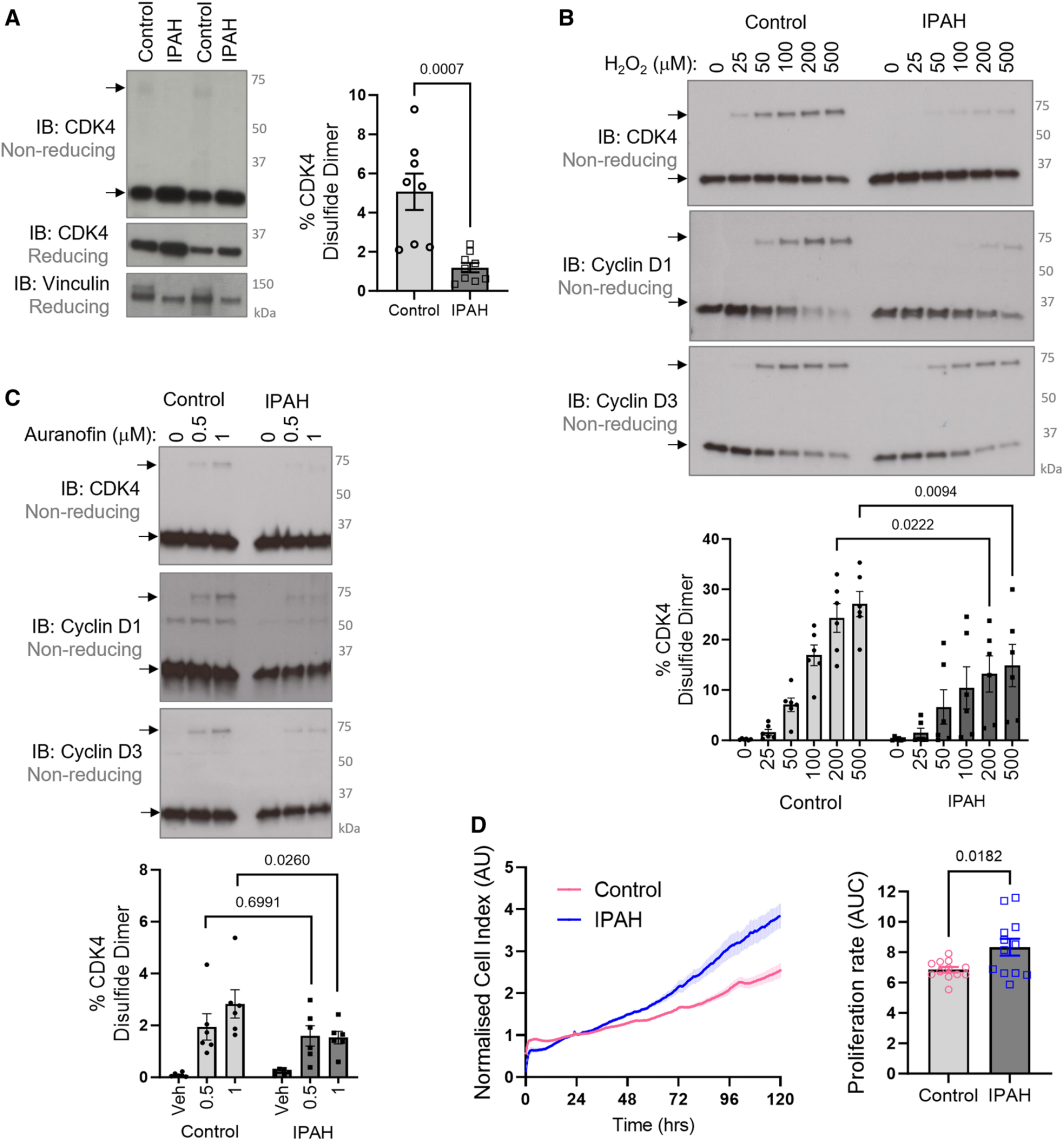
**Idiopathic pulmonary arterial hypertension (PAH; IPAH) pulmonary arteries and human pulmonary arterial smooth muscle cells (HPASMCs) have less CDK4 (cyclin-dependent kinase 4) disulfide than donor controls. A**, Formation of the CDK4 disulfide bond is decreased in pulmonary arteries of IPAH patients compared with healthy volunteer controls. Nonreducing immunoblots show monomeric and disulfide dimeric CDK4, while reducing immunoblots show CDK4 expression with vinculin used as a loading control. Quantification is provided for the percentage of CDK4 observed as a disulfide dimer. The data passed the normality test performed by the Shapiro-Wilk test. *P* values were calculated using the parametric unpaired 2-tailed *t* test to compare between 2 groups (control, n=8; IPAH, n=9), and the results are shown as means±SEM. **B**, IPAH HPASMCs have an attenuated response to oxidant treatment compared with donor control cells. HPASMCs were treated with H_2_O_2_ for 15 minutes in DMEM growth medium. The formation of disulfide cyclin D-CDK4 was detected by nonreducing immunoblotting probed for CDK4, cyclin D1, and cyclin D3. The graph shows quantification of the percentage of CDK4 that is detected as a disulfide dimer. The data passed the normality test performed by the Shapiro-Wilk test. *P* values were calculated using the parametric 2-way ANOVA followed by Sidak post hoc multiple comparisons test (n=6 per group); the results are shown as means±SEM. **C**, IPAH HPASMCs accumulate less disulfide CDK4 than donor control cells in response to auranofin treatment. HPASMCs were treated with auranofin for 1 hour in serum-free DMEM. Monomeric and disulfide dimeric CDK4, cyclin D1, and cyclin D3 were detected by nonreducing immunoblotting. The graph shows quantification of the proportion of CDK4 that was observed to form a disulfide (n=6 per group). The data did not pass the normality test performed by the Shapiro-Wilk test. *P* values are calculated using an unpaired 2-tailed nonparametric Mann-Whitney *U* test to compare 0.5 or 1 μM Aur-treatment between Control and IPAH groups (n=6 per group). The results are shown as means±SEM. **D**, HPASMCs isolated from patients with IPAH have an increased proliferation rate compared with donor control HPASMCs. Proliferation was measured by electrical impedance in real time using xCELLigence (representative of n=6 independent biological replicates per group, in technical duplicate). Proliferation rate was then analyzed using area under the curve (AUC). The data passed the normality test performed by the Shapiro-Wilk test. *P* value is calculated using the parametric unpaired 2-tailed *t* test to compare between 2 groups (n=6 independent biological replicates per group, in technical duplicate), and the results are shown as means±SEM.

**Figure 8.**
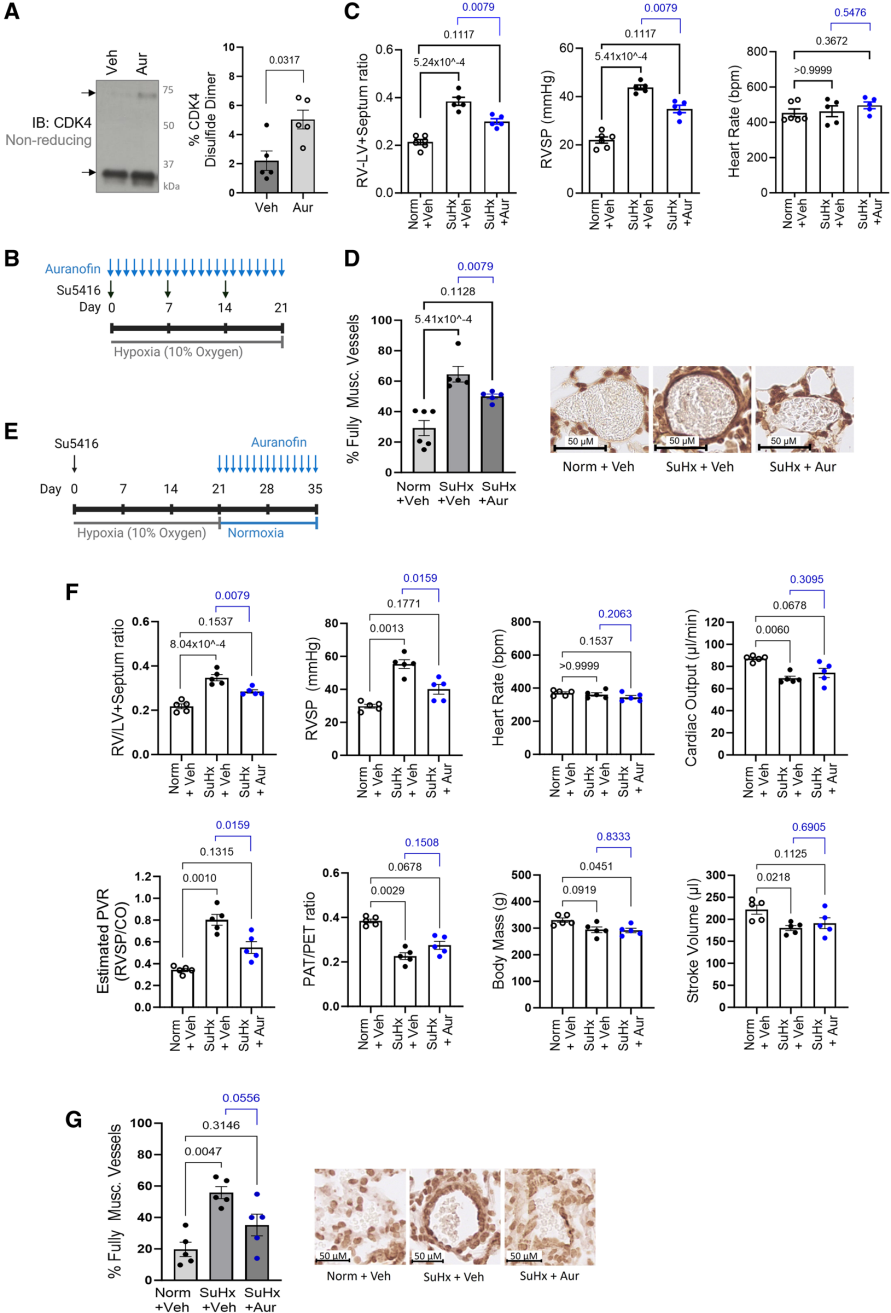
**Pro-oxidant treatment provides therapeutic benefits in experimental models of pulmonary hypertension (PH). A**, Thioredoxin reductase inhibition by auranofin treatment potentiated the accumulation of disulfide CDK4 (cyclin-dependent kinase 4). Mice were treated, by intraperitoneal injection, with auranofin (10 mg/kg) or vehicle (10% DMSO/saline) for 6 hours before rapid isolation and snap-freezing of pulmonary arteries. Mouse pulmonary arteries were analyzed by nonreducing immunoblotting to assess monomeric and disulfide dimeric CDK4 (representative of n=5/group). Graphs show quantification of the proportion of CDK4 that was observed as a disulfide dimer. As the data sample size with an n<6 cannot be reliably tested for normality, a *P* value is calculated using an unpaired 2-tailed nonparametric Mann-Whitney *U* test to compare between Vehicle and Aur-treatment (n=5 per group), and the results are shown as means±SEM. **B**, The Sugen (SU5146)/hypoxia mouse model of PH was induced by exposure to hypoxia (10% O_2_) for 3 weeks along with weekly subcutaneous injections of 20 mg/kg Sugen (Su5416). Control mice were maintained in normoxia (Norm, 21% oxygen). Auranofin (Aur, 10 mg/kg per day) or vehicle (Veh, 10% DMSO/saline) was injected daily for the duration of the model. **C**, Right ventricular (RV) systolic pressure (RVSP, mm Hg), RV hypertrophy (RV/LV+septum ratio), and heart rate (beats per minute) were measured to assess the severity of PH. As the data sample size with an n<6 cannot be reliably tested for normality, *P* values are calculated using nonparametric Kruskal-Wallis test followed by Dunn multiple comparisons to compare Vehicle and Aur-treated SuHx groups to Norm+Veh; unpaired 2-tailed nonparametric Mann-Whitney *U* test was used to compare between Vehicle and Aur-treatment SuHx groups (Norm+Veh, n=6; SuHx+Veh, SuHx+Aur, n=5), and the results are shown as means±SEM. **D**, Muscularization of small pulmonary arteries (10–100 µm) was measured using α-SMA immunohistochemical staining, of mouse lung sections after induction of the Sugen/hypoxia pulmonary hypertension model for 3 weeks along with daily injections of 10 mg/kg auranofin or vehicle. *P* values are calculated using nonparametric Kruskal-Wallis test followed by Dunn multiple comparisons to compare the proportion of fully muscularized vessels in Vehicle or Aur-treated SuHx groups to Norm+Veh; unpaired 2-tailed nonparametric Mann-Whitney *U* test was used to compare between Vehicle and Aur-treatment SuHx groups (Norm+Veh, n=6; SuHx+Veh, SuHx+Aur, n=5), the results are shown as means±SEM. Representative images of each treatment group are provided, along with a 50 µm scale bar. **E**, Pulmonary hypertension was modeled using Sugen (SU5416)/hypoxia (SuHx) in Wistar rats by a single subcutaneous injection of 20 mg/kg Sugen followed by exposure to hypoxia (10% oxygen) for 3 weeks. Rats were then returned to normoxia (21% oxygen) and treated daily with 8 mg/kg Auranofin (Aur) or Vehicle (Veh, 10% saline/DMSO). **F**, RV hypertrophy, RVSP, heart rate, cardiac output, pulmonary vascular resistance (PVR), pulmonary acceleration time (PAT)/pulmonary ejection time (PET) ratio, body mass, and stroke volume were analyzed to assess disease severity. As the data sample size with an n<6 cannot be reliably tested for normality, *P* values are calculated using a nonparametric Kruskal-Wallis test followed by Dunn multiple comparisons to compare parameters in Vehicle and Aur-treated SuHx groups to Norm+Veh; unpaired 2-tailed nonparametric Mann-Whitney *U* test compare between Vehicle and Aur-treatment SuHx groups (n=5 per group), the results are shown as means±SEM. **G**, Muscularization of small pulmonary arteries (10–100 µm) was measured using alpha smooth muscle actin (α-SMA) immunohistochemical staining of rat lung sections after induction of the Sugen/hypoxia pulmonary hypertension model followed by treatment with auranofin or vehicle. *P* values are calculated using a nonparametric Kruskal-Wallis test followed by Dunn multiple comparisons to compare the proportion of fully muscularized vessels in Vehicle and Aur-treated SuHx groups to Norm+Veh; unpaired 2-tailed nonparametric Mann-Whitney *U* test was used to compare between Vehicle and Aur-treatment SuHx groups (n=5 per group), the results are shown as means±SEM. Representative images of each treatment group are provided, along with a 50 µm scale bar.

Considering that smooth muscle cell and endothelial cell proliferation is causative to pulmonary vascular remodeling in PH, the role of the cyclin D-CDK4 disulfide was next determined in pulmonary vasculature in vivo. Notably, pulmonary arteries isolated from patients with PAH display a decreased level of CDK4 disulfide compared with healthy controls (Figure 7A). Furthermore, treatment of HPASMCs isolated from PAH patients revealed an attenuated response to H_2_O_2_ (Figure 7B), or auranofin (Figure 7C), suggesting PAH HPASMCs encounter a more reducing environment than donor control cells and hence less prone to form disulfide-CDK4. In line with previous literature,^6,36^ these idiopathic PAH HPASMCs are hyperproliferative compared with control donor cells (Figure 7D), which is likely, at least partially, to be caused by the lack of cyclin D-CDK4 disulfide. Importantly, the abundance of disulfide CDK4 can be potentiated in mouse pulmonary arteries using auranofin treatment (Figure 8A), which inhibits disulfide reduction by thioredoxin/thioredoxin reductase. To characterize the potential therapeutic benefits of auranofin, which triggers the inhibitory redox-switch at C135 in CDK4, 3 experimental models of PH were employed. First, mice were subjected to hypoxia for 14 days and treated continuously with 8.5 mg/kg per day auranofin via subcutaneous osmotic minipump (Figure S18). Treatment with auranofin prevented the progression of PH disease, as demonstrated by an attenuation of RVSP and RV hypertrophy, compared with vehicle-treated hypoxic controls (Figure S18). Next, the more severe Sugen/hypoxia experimental PH model was employed (Figure 8B), and mice were treated daily with auranofin treatment, or vehicle, by intraperitoneal injection. As expected, vehicle-treated mice developed PH evidenced by pronounced increase in RV hypertrophy and RVSP, and once again, hemodynamic benefits were observed after cotreatment with auranofin, through an attenuated increase in RVSP and RV hypertrophy compared with normoxic vehicle-treated controls (Figure 8C). To examine whether these hemodynamic benefits were due to alleviation of pulmonary vascular remodeling, a quantitative analysis of histologically stained lung sections was performed. Immunohistochemical staining of α-smooth muscle actin (α-SMA) showed that auranofin treatment decreased the percentage of fully muscularized small pulmonary arteries, in response to Sugen/hypoxia (Figure 8D), likely due to a decrease in smooth muscle cell proliferation. Finally, PH was induced in rats using the Sugen/hypoxia model to assess whether auranofin could improve cardiopulmonary functions. Rats were treated with a single dose of Sugen (SU5416) followed by 3 weeks of exposure to hypoxia (Figure 8E). Once returned to normoxia, rats were treated daily with vehicle or auranofin for 2 weeks, which reversed disease severity resulting in a significant decrease in RV hypertrophy, RVSP, and pulmonary vascular resistance. Along with this, there was a trend toward an improvement in pulmonary acceleration time/pulmonary ejection time ratio (Figure 8F). Immunohistological staining of α-SMA in the lung showed only a moderate increase in the proportion of fully muscularized vessels in the auranofin-treated group (Figure 8G), consistent with a decrease in smooth muscle cell proliferation.

## Discussion

Cell cycle inhibition, induced by the CDK4/6 inhibitor palbociclib, has recently been demonstrated to be therapeutically beneficial in experimental PH.^36^ In this study, we identify that CDK4, a key cell cycle kinase, is regulated by a novel redox-switch that inhibits the function of this kinase in health and disease (Figure S19). Our work reveals several novel findings. First, we demonstrate the formation of an inducible heterodimeric disulfide bond between CDK4 and cyclin D, which contributes to oxidant-induced cell cycle arrest, thus providing insights into the redox regulation of the cell cycle. Second, CDK4 C135 is indispensable for CDK4 kinase activity, meaning perturbation of this thiol impairs kinase activity leading to attenuation of cell proliferation in vitro and in vivo. Third, the cyclin D-CDK4 disulfide is present in pulmonary arteries in vivo and its abundance is decreased in PAH patients, which may drive disease pathogenesis. Finally, pharmacological treatment with auranofin increased the accumulation of disulfide cyclin D-CDK4, which provided cardiopulmonary benefits in 3 experimental models of PH. Altogether, our findings reveal a novel mechanism by which oxidants such as H_2_O_2_ induce cell cycle arrest to halt the proliferation of pulmonary vascular cells. Furthermore, oxidative inhibition of cyclin D-CDK4 kinase activity provides a novel site for allosterically targeting CDK4 and provides a mechanistic rationale for the use of antiproliferative redox therapies, such as auranofin, which induce disulfide formation.

Cell cycle arrest is a critical response that protects cells from unrepaired DNA damage and genomic instability^57^ and is regulated by transcription, as well as more rapid oxidative posttranslational modifications of proteins. Using an Affymetrix microarray, we highlight the importance of cell cycle reprogramming in the pathogenesis of PH and the potential therapeutic benefits of selectively targeting cell cycle regulators (Figure 1A). In the hypoxia-induced model of PH, at the 3-day time point used, arteries are reportedly constricted but not yet remodeled.^24,58^ This implies that the transcriptional changes in cell cycle–related gene expression observed here are likely to be causative in disease pathophysiological changes. To date, only a handful of redox-regulated cell cycle effector proteins have been fully characterized, including p16,^59^ Aurora A kinase,^60,61^ CDC25 (cell division cycle 25 phosphatase),^62,63^ CDK2,^64^ and ataxia-telangiectasia mutated (ATM) kinase.^65^ Here, we found that the G1 phase cell cycle regulator, cyclin D-CDK4, forms an intermolecular disulfide bond in response to oxidation (Figure 1B and 1C). Both mutagenesis and LC-MS/MS analysis confirmed that this disulfide forms between CDK4 C135 and cyclin D1 C7/8 or cyclin D3 C5/6 (Figure 2). Due to the proximity of cyclin D1 C7/8 and CDK4 C135 (Figure 2A), together with the low p*K*_a_ of CDK4 C135, the formation of a disulfide bond between these residues is perhaps unsurprising. The cyclin D-CDK4 disulfide is reversible owing to endogenous reduction by the thioredoxin/thioredoxin reductase antioxidant system (Figure 1F and 1G). Our data show that the formation of a disulfide bond in cyclin D-CDK4 inhibits kinase activity toward its key substrate, Rb, both in recombinant protein in vitro (Figure 3A) and in HPASMCs (Figure 4A and 4B). Accordingly, and in line with previous reports in other cell types,^57,66^ H_2_O_2_ treatment induced G1 phase cell cycle arrest (Figure 4C and 4D), decreasing the proliferation rate of HPASMCs (Figure 4E) and human pulmonary arterial endothelial cells (Figure S11). While the proportion of CDK4 that forms a disulfide bond may seem to be small (Figure 1B and 1C), the role this mechanism plays in cell cycle control is anticipated to be greater as kinase activity is restricted only to the proportion of CDK4 protein that is in a heterodimeric complex with cyclin D. We have not yet been able to test the cytosolic versus nuclear localization of cyclin D-CDK4 disulfide heterodimer; however, it is possible that, at least basally, the oxidized form of cyclinD-CDK4 is at lower abundance in the nucleus, where it serves a regulatory feedback mechanism to control growth and proliferation. This speculation is based on findings that the nucleus is a relatively reducing and resistant to oxidation cell compartment, compared with the cytoplasm or mitochondria.^20^

Interestingly, we found that CDK4 C135 is a critical cysteine residue that is essential for optimal cyclin D-CDK4 kinase activity (Figure 5A and 5B). As such, mutation of CDK4 C135 resulted in a kinase-impaired protein complex that did not efficiently phosphorylate Rb, while mutation of an alternative residue, CDK4 C78, had no effect on kinase activity or oxidant-sensitivity. Moreover, HAP1 cells stably expressing CDK4 C135A had a kinase-impaired phenotype, as demonstrated by a decreased proliferation rate compared with their WT counterpart (Figure 5G). Furthermore, primary pulmonary vascular smooth muscle or endothelial cells, isolated from novel transgenic redox-dead C135A CDK4 KI mice, phenocopy C135A HAP1 cells by also proliferating at slower rates, compared with the cells originating from WT littermates (Figure 6A; Figure S15). This consistently reproducible effect is similar to reports of a decreased proliferation rate and reduced mitogen sensitivity of CDK4^−/−^ mouse embryonic fibroblasts.^56,67^ As both C135A CDK4 and disulfide CDK4 are kinase-impaired (Figure S19), C135A CDK4 expressing HAP1 cells and C135A CDK4 KI pulmonary vascular cells provide a surrogate measure for the effect of CDK4 oxidation on cell cycle control and proliferation in pulmonary vasculature. This was further reiterated in the phenotype of CDK4 KI mice, which demonstrated a small but significant reduction in pulmonary vascular muscularization, likely due to reduced pulmonary vascular cell proliferation, leading to the development of a less severe disease phenotype in a Sugen/hypoxia experimental PH model (Figure 6B through 6F; Figure S16), altogether supporting the role of C135 CDK4 as a critical cysteine residue that regulates cell proliferation. However, it must be acknowledged that the use of a C135A KI mouse is somewhat limited because the mutation not only generates an oxidant-insensitive kinase but also severely reduces its activity, and therefore in this case, redox-dead C135A CDK4 KI cannot be used to dissect the direct role of disulfide cyclin D-CDK4 in the PH. Consistent with this, structural in silico modeling revealed that it is indeed CDK4 C135 that mediates the allosteric structural modifications in cyclin D-CDK4 that plausibly explain the inhibitory effects of thiol oxidation or mutation. In fact, perturbation of CDK4 C135, either alone or in combination with cyclin D1 C7/8, increased rigidity of numerous key regions of the kinase complex (Figure 3D; Figure S9). Whereas perturbation of alternative cysteine residues in CDK4 had little effect on the rigidity of these regions. In particular, probing of C135, caused rigidification of the hinge, whose torsion is required to allow conformational rearrangements that lead to the active kinase conformation.^49^ Moreover, at the interface between CDK4 and cyclin, the αC-helix is partly rigidified, which may impair the overall dynamics to rearrange the heterodimeric complex into its active form. Interestingly, the allosteric landscape shows that the catalytic loop, including residues _138_HRDLKPEN_145_, is rigidified indicating that catalysis is affected by the formation of the disulfide bond. This is supported by the observed decrease in Rb phosphorylation in oxidizing conditions or the presence of C135A CDK4. The substrate-assisted model of CDK4 activation proposes that in order for the active cyclin D-CDK4 conformation to be transiently achieved, substrate binding, ATP binding, and activation loop phosphorylation are required.^49,50,68^ Therefore, since conformational changes from the inactive to the active state involve remodeling of the αC-helix and the activation segment to permit catalysis,^50,69^ it is likely that CDK4 C135 oxidation or mutation impairs these required structural changes. This is also consistent with experiments showing *N*-ethylmaleimide or maleimide, which adducts to cysteine thiols including C135, inhibits kinase activity (Figure 5C and 5D). Altogether, these findings highlight the potential for translation of this novel mechanistic insight into the clinic, using C135 as a potential drug target to selectively inhibit CDK4 with an aim to combat vascular hyperproliferative diseases.

Given one of the important roles that cell hyperproliferation plays in vascular remodeling during the progression of group 1 PAH and group 3 PH,^1^ we sought to investigate the therapeutic implications of the cyclin D-CDK4 disulfide bond. Critically, we found that the cyclin D-CDK4 disulfide forms basally in healthy human pulmonary arteries, and its abundance is decreased in the pulmonary arteries of group 1 idiopathic PAH patients (Figure 7A). Similarly, HPASMCs isolated from patients with idiopathic PAH displayed an attenuated response to H_2_O_2_-induced disulfide formation (Figure 7B), and auranofin-induced disulfide accumulation (Figure 7C). This may be due to an increase in cellular reducing equivalents in PH, or a decrease in SOD expression and activity,^25,70^ shifting the balance between proproliferative superoxide and the antiproliferative signaling molecule H_2_O_2_.^57,66,71^ Indeed, it has been shown that adaptation to hypoxia involves metabolic changes that promote a proreducing environment, through increased production of reductant NADPH from the pentose phosphate pathway,^72^ and a shift from oxidative phosphorylation toward glycolysis.^73–76^ Additional evidence for a protective role of ROS in PH comes from the use of antioxidants. More specifically, resveratrol, which is a polyphenol compound that is considered to have antioxidant and anti-inflammatory properties,^77^ but was paradoxically shown to induce protein oxidation of PKGIα and thus act as a pro-oxidant to induce systemic vasodilation.^78^ In both the monocrotaline rat model,^79^ and the hypoxia rat model of PH,^80^ resveratrol attenuated disease severity, which may be explained by PKGIα oxidation,^24^ as well as the antiproliferative oxidant-induced inhibition of CDK4. It is likely that remodeling of the redox network occurs in disease, in a similar way to aging,^81^ resulting in a decrease in oxidation (ie, reduction) of some proteins including cyclin D-CDK4. The exact cause for the reduced state of cyclin D-CDK4 in PAH is likely linked with altered redox metabolism and is yet to be established. In any case, given the kinase-inhibitory nature of the cyclin D-CDK4 disulfide bond (Figure 3A through 3C), this decrease in its abundance is anticipated to contribute to the reported hyperactivity of CDK4 in PH^36^ and the hyperproliferation of PH cells (Figure 7D).

Loss-of-function BMPR2 (bone morphogenetic protein receptor 2) mutations are known to cause familial PAH, characterized by excessive PASMC proliferation, particularly in response to transforming growth factor-β.^82^ Recently, it was elegantly shown that human PASMC with loss of BMPR2 are hyperproliferative, and resistant to apoptosis, suggesting that deficiency of BMPR2 in PASMC can contribute to the development of PAH.^41^ However, apart from CDK4 being an end-effector protein that ultimately drives cell cycle progression, a connection between BMPR2 signaling and the cyclin D-CDK4 pathway currently remains unknown.

Cyclin D-CDK4 is a key cell cycle regulatory protein that controls entry into and progression through the G1 phase of the cell cycle by phosphorylating Rb. However, there is emerging evidence of novel noncanonical roles of this protein complex, beyond cell cycle control and progression, including the regulation of cellular differentiation^83^ and metabolism.^84–86^ Recent studies showed CDK4 inhibition caused an increase in mitochondrial oxygen consumption in muscle cells, while CDK4 knockout mice demonstrate an elevated metabolic rate under basal conditions.^84^ E2F1, a target of cyclin D1-CDK4, also plays a role in energy production by repressing oxidative metabolism in mitochondria.^85^ It was also reported that insulin may use components of the cell cycle machinery to control glucose homeostasis in postmitotic cells independently of cell division.^86^ Further experiments would be required to establish the effect of CDK4 oxidation on these noncanonical roles in metabolism, and their potential involvement in the protective benefits of CDK4 oxidation and C135A mutation that we have evidenced in PH.

Importantly, accumulation of the cyclin D-CDK4 disulfide can be potentiated by treatment with the thioredoxin reductase inhibitor, a gold-containing phosphine compound auranofin (Figure 8A), which attenuated disease severity in multiple pathophysiological parameters in 3 experimental PH models (Figure 8; Figure S18). These findings suggest that the increased abundance of the cyclin D-CDK4 disulfide is one of the mechanisms by which auranofin decreases vascular remodeling in PH to provide therapeutic benefits (Figure 8D and 8G). Based on its high reactivity with cellular nucleophiles such as selenocysteine and cysteine and its known capacity to selectively inhibit thioredoxin reductase and glutathione peroxidase, auranofin inhibits the endogenous reduction of disulfide bonds by the former. Although, it is likely that auranofin also potentiates the oxidation of other targets in addition to CDK4. However, in this case, redox-dead C135A CDK4 KI mice would not help to dissect the specific role of disulfide cyclin D-CDK4 in the response to auranofin. This is because the mutation does not only generate an oxidant-insensitive kinase but also severely reduces its activity. In this study, auranofin was administered to increase the level of cyclin D-CDK4 disulfide in WT mice, promoting this adaptive redox response to measure the therapeutic benefits. Interestingly, auranofin can also shift the steady-state ROS levels that are carefully maintained by the cellular antioxidant system toward pro-oxidizing conditions. In particular, it can increase ROS levels (including H_2_O_2_ and superoxide) and inhibit growth and proliferation in several cancer cell lines; however, this effect can vary depending on the cell line.^87^

Currently, auranofin is food and drug administration–approved for the treatment of rheumatoid arthritis and is in clinical trials for the treatment of cancers including leukemia or ovarian cancer.^88^ Ultimately, the work presented here provides a mechanistic rationale for the potential future clinical development of auranofin in the field of hyperproliferative vascular diseases, to provide therapeutic benefits in PH. This mechanism of targeting CDK4 may offer a more favorable toxicity profile for chronic use compared with current CDK4/6 inhibitors. Additionally, interest surrounding the use of novel electrophilic compounds is growing with the identification of covalent inhibitors of EGFR,^89^ GSK-3 (glycogen synthase kinase-3),^90^ BFL-1 (B-cell lymphoma 2-like protein 1),^91^ and CDK2.^92^ In fact, electrophilic drugs can directly induce protein oxidation, as recently demonstrated for PKGIα,^93^ or prevent oxidation by covalently binding to the redox-active cysteine, as shown for GSK-3^90^ and EGFR.^89^ Since CDK4 C135 represents a critical residue that allosterically regulates cyclin D-CDK4 activation, it offers a novel site that warrants the development of a new therapy for selectively and covalently targeting CDK4 with a selective electrophilic inhibitor that may prove useful in the treatment of PH. Future work using high-throughput screening of an electrophile library is envisaged to find a novel electrophilic compound, which may represent a unique drug class that stimulates an endogenous mechanism responsible, in this case, for cell cycle arrest in vivo.

Overall, the results of this study highlight the importance of oxidative posttranslational modifications in proteins, that serve as regulatory feedback mechanisms to control growth and proliferation of pulmonary vascular cells in health and PH. A disulfide bond in cyclin D-CDK4 inhibits kinase activity, which contributes to the protective benefits of auranofin treatment in PH, by triggering a brake on the cell cycle. Our work provides evidence for a novel mechanism of inhibiting CDK4 that has the potential to be therapeutically harnessed in PH.

## ARTICLE INFORMATION

### Acknowledgments

The authors thank Dr Hyun-Ju Cho for her initial help with setting up the flow cytometry experiment, Dr Rhys Anderson for providing the protocol for the lentiviral stable cell line, Dr Friedrich Baark for his initial help with rat ultrasound imaging, Dr Alison Brewer for lending us a Manual Magnetic-activated cell sorting Separator for mouse lung endothelial cell separation, and Dr Konstantinos Theofilatos for advising about data deposition. The authors thank Dr Rebecca Charles and Dr Sebastian Guttzeit for their indispensable advice regarding kinase activity assays.

### Author Contributions

O. Rudyk designed the study and sought funding. H. Knight and O. Rudyk designed, planned, and performed the majority of the experiments and wrote the article. H. Knight, G. Abis, M. Kaur, H.L.H. Green, S. Krasemann, K. Hartmann, J. Clark, S. Lynham, A. Weiss, C. Ruppert, and O. Rudyk performed the experiments and analyzed the data. A. Weiss, C. Ruppert, and R.T. Schermuly provided human tissues. H. Knight, L. Zhao, S. Lynham, R.T. Schermuly, P. Eaton, and O. Rudyk discussed the data. All authors read the article and approved its final version.

### Sources of Funding

O. Rudyk is supported by a British Heart Foundation Intermediate Basic Science Research Fellowship (FS/14/57/31138), British Heart Foundation Project Grant (PG/20/10427), and King’s British Heart Foundation center of Research Excellence (RE/18/2/34213). P. Eaton is supported by The Bart’s Charity Cardiovascular Program Award G00913 and by program grants from the British Heart Foundation and the Medical Research Council. Ralph Schermuly is supported by Deutsche Forschungsgemeinschaft (DFG, German Research Foundation)—Project-ID 268555672—SFB 1213, project A08. This research was also supported by Research and Development, Guy’s and St Thomas’ National Health Service Foundation Trust. The views expressed are those of the authors and not necessarily those of the National Health Service.

### Disclosures

None.

### Supplemental Material

Expanded Materials and Methods

Figures S1–S19

Data Set file

Major Resources Table

Full unedited gels

References 94–99

## Supplementary Material


