# Supplementary material for: Cyclin D-CDK4 Disulfide Bond Attenuates Pulmonary Vascular Cell Proliferation
Source: Circ Res. 2023 Nov 13;133(12):966–88. doi: 10.1161/CIRCRESAHA.122.321836 (PMC10699508; doi:10.1161/CIRCRESAHA.122.321836)
Supplement: Supplementary file 2 [file res-133-0966-s002.pdf]

Supplemental Materials

A cyclin D-CDK4 disulfide bond attenuates pulmonary vascular cell proliferation

Hannah Knight[1], Giancarlo Abis[2#], Manpreet Kaur[1#], Hannah L. H. Green[1#], Susanne Krasemann[3], Kristin Hartmann[3], Steven Lynham[4], James Clark[1], Lan Zhao[5], Clemens Ruppert[6], Astrid Weiss[7], Ralph T. Schermuly[7], Philip Eaton[8], Olena Rudyk[1*]

[1]School of Cardiovascular and Metabolic Medicine & Sciences, British Heart Foundation Centre of Research Excellence, King's College London, London, UK
[2]Division of Biosciences, Institute of Structural and Molecular Biology, University College London, London, UK
[3]Institute of Neuropathology, University Medical Centre Hamburg-Eppendorf, Hamburg, Germany
[4]Proteomics Core Facility, Centre of Excellence for Mass Spectrometry, King's College London, London, UK
[5]National Heart and Lung Institute, Faculty of Medicine, Imperial College London, London, UK
[6]Universities of Giessen & Marburg Lung Center Giessen Biobank, Justus-Liebig-University Giessen, Giessen, Germany
[7]Department of Internal Medicine, Justus-Liebig-University Giessen, Giessen, Member of the German Center for Lung Research (DZL), Giessen, Germany
[8]William Harvey Research Institute, Barts & The London School of Medicine & Dentistry, Queen Mary University of London, London, UK

Short title: CDK4 oxidation attenuates cell proliferation

[#] Giancarlo Abis, Manpreet Kaur and Hannah L. H. Green contributed equally.

***Correspondence should be addressed to:**

Dr Olena Rudyk (email: olena.rudyk@kcl.ac.uk)

School of Cardiovascular and Metabolic Medicine & Sciences, King's College London, The Rayne Institute, St. Thomas' Hospital, SE1 7EH, London, UK

Supplemental Materials File Includes Expanded Materials and Methods and Supplemental Figures **S1** to **S19** with Figure Legends.

Expanded Materials and Methods

G0/1 phase synchronization

HPASMCs were synchronized to the G0/1 phase of the cell cycle using serum starvation [66]. Cells were maintained in DMEM supplemented with 0.1% FBS and 1% Penicillin/Streptomycin (referred to as starvation media) for 48 or 72 hours, as required, before growth media was replenished to stimulate cell cycle entry. For detection of the cyclin D1-CDK4 disulfide bond by immunoblotting, cells were treated with H_2O_2 for 15 minutes before lysis in non-reducing sample buffer containing 100 mM maleimide.

Protein overexpression

CDK4, cyclin D1 and/or cyclin D3 were overexpressed in HPASMCs using Lipofectamine 3000 transfection reagent (Invitrogen #L3000008) or in HAP1 cells using lipofectamine 2000 (Invitrogen #11668019)) according to manufacturer's instructions. Cells were seeded at $1x10^5$ cells/ml in 6-well or 12-well plates. Cells were transfected with pcDNA3.1-CDK4-FLAG (GenScript Clone OHu21245), pcDNA3.1-cyclinD1-HA (Addgene #11181), pcDNA3.1-cyclinD3-HA (GenScript clone OHu21276C). The following concentrations of plasmid DNA were used in a 12-well plate or 6-well plate, respectively; 400ng/800ng cyclin D1 plasmid DNA, 400ng/800ng cyclin D3 plasmid DNA, and/or 400/800ng CDK4 plasmid DNA. In brief, the plasmid DNA, 2μL/μg p3000 reagent (lipofectamine 3000 only), and 1.5μL/well (12-well plate) or 4μL/well (6-well plate) lipofectamine 2000 or 3000 were diluted in opti-MEM reduced serum medium and incubated at room temperature for 15 minutes before addition to cells. After 24 hours, media was replenished, and cells were treated with H_2O_2 for 15 minutes.

PEG-switch

To detect reversible oxidation of CDK4 the PEG-switch method was used, as described before [48]. Briefly, HPASMCs were treated with H_2O_2 for 15 minutes, harvested in cell lysis buffer (100 mM Tris-HCl pH 7.4, 100 mM maleimide, 1% SDS) and incubated at 50°C for 25 minutes with constant agitation. A sample was taken and added to non-reducing sample buffer, this is termed 'input'. 200 mM DTT was added to the remaining samples and incubated for 30 minutes to reduce any reversible oxidative modifications. Using a Zeba spin desalting column (7K MWCO, 0.5 mL, #89882 ThermoFisher), samples were desalted immediately into buffer containing 70 mM Tris-HCl pH 7.4, 10 mM PEG-maleimide, 1% SDS. Samples were incubated for 2 hours at room temperature before the addition of reducing sample buffer (supplemented with 5% β-mercaptoethanol), this was termed 'PEG output'. Monomeric and disulfide dimeric CDK4 were detected by non-reducing immunoblotting of input samples, while monomeric and PEGylated CDK4 were detected by reducing immunoblotting of PEG output samples.

Site-directed mutagenesis

Cysteine to alanine or serine mutants were generated by site-directed mutagenesis using the Q5 mutagenesis kit (New England Biolabs, #E0554S) or Phusion mutagenesis kit (Thermo Fisher Scientific, #F541) following manufacturer's instructions. Primers (**Table 1**) were designed using the NEBaseChanger online tool and modified for the Phusion kit, as required. Successful mutagenesis was confirmed by DNA sequencing (Eurofins MWG).

Protein	Mutation	Forward Primer	Reverse Primer	Polymerase
CDK4-FLAG	C135A	TCATGCCAATGCCATCGTTCACC	AGGAAATCTAGGCCTCTTAG	Q5
CDK4-FLAG	C135S	TCATGCCAATAGCATCGTTCACC	AGGAAATCTAGGCCTCTTAG	Q5
CDK4-FLAG	C78A	GATGGACGTCGCTGCCACATCCCG AAC	AGCCGGACAACATTGGGA	Q5
Cyclin D1-HA	C7A	CCAGCTCCTGGCCTGCGAAGTGGA AAC	TGTTCCATGGCTGGGGCT	Q5
Cyclin D1-HA	C8A	GCTCCTGTGCGCCGA AGTGGAAACC	TGGTGTTCCATGGCTGGG	Q5
Cyclin D1-HA	C7/8A	CCAGCTCCTGGCCGCCGAAGTGGA AACCATCCG	TGTTCCATGGCTGGGGCT	Q5
Cyclin D1-HA	C38A	CGGAGGAGACCGCCGCGCCCTCG	CGAGGGCGCGGCGGTCTCCTCCG	Phusion
Cyclin D3-HA	C5/6A	GGAGCTGCTGGCTGCCGAAGGCA CCCGGC	ATCTCGAGCGGCCGCCAC	Q5

Table 1. Primers for site-directed mutagenesis of CDK4, cyclin D1 and cyclin D3.

Measurements of cysteine proximity

The cyclin D1-CDK4 crystal structure (PDB 2W96 [49]) was explored using PyMOL Molecular Graphics System 2.3.4 (Schrödinger, LLC). Measurements of distances between sulfur atoms of cysteine residues were carried out using the wizard measurement tool in PyMOL.

AlloSigMA analysis

AlloSigMA is a structure-based statistical mechanical modelling tool which estimates the per-residue allosteric free energies (Δg_i) of a protein complex after a perturbation. Increased ($\Delta g_i > 0$) and decreased ($\Delta g_i < 0$) allosteric free energies correspond to enhanced dynamics or rigidification respectively [55, 94]. Our experiments were performed on the AlloSigMA web-server [94], using the X-ray crystal structure of the cyclin D1-CDK4 complex (PDB 2W96[3]), upon alternate locations correction with FirstGlance 3.7. To mimic the disulfide bond between CDK4 and cyclin D1, the analysis was carried out in probing mode by perturbing CDK4 C135 and cyclin D1 C7/8, followed by controls on CDK4 C78, CDK4 C135 or CDK4 C215 in isolation. The Δg_i values were mapped onto the cyclin D1-CDK4 complex structure in blue and red color scales to indicate, respectively, rigidified and dynamized residues, using PyMOL.

Sequence alignments

The FASTA sequence of human cyclin D1 (P24385), cyclin D2 (P30279), cyclin D3 (P30281), CDK4 (P11802), CDK6 (Q00534), CDK2 (P24941) and CDK1 (P06493), along with those of appropriate species were obtained from the UniProt database. The National Library of Medicine Basic Local Alignment Search Tool (BLAST) was used to align and compare cysteine conservation between proteins or species.

Protein knockdown by siRNA

Cyclin D1 protein expression was knocked down in HPASMCs using Silencer Select siRNA (assay ID 229, Thermo Fisher Scientific) and scrambled silencer select siRNA (Negative control no. 1, #4390843, Thermo Fisher Scientific). siRNA was resuspended in ultrapure nuclease-free H_2O to a working stock concentration of 10 μM. Cells were transfected with 20 nM siRNA using 6 μL/well Lipofectamine RNAiMAX, according to manufacturer's instructions. 48 hours later, media was replenished, and cells were treated with H_2O_2 for 15 minutes.

Measurements of cyclin D1 and CDK4 protein concentration

HPASMCs were cultured for 48 hours to reach 70-80% confluency before being harvested in cell lysis buffer (100 mM Tris-HCl pH 7.4, 100 mM maleimide, 2% SDS). Total protein concentration was measured using a Pierce BCA Protein Assay Kit (Thermo Fisher Scientific, #23225), according to manufacturer's instructions. The total protein concentration (μg/mL) in HPASMC lysate was estimated from the BSA standard curve. 4 μg of total protein (HPASMC lysate) was loaded into a pre-cast gel (Bio-Rad) alongside known amounts of recombinant cyclin D1 His-tagged protein (LSBio #LS-G20572) or recombinant CDK4 His-tagged protein (Invitrogen #RP-75530) and separated by SDS-PAGE under reducing conditions. Immunoblots were probed with antibodies for cyclin D1 or CDK4 and signal intensities were measured by densitometry. The signal intensity of known amounts of cyclin D1 or CDK4 were plotted, and a linear regression curve was fitted using GraphPad Prism. From the equation of the line, the amount of CDK4 and cyclin D1 in 4 μg total protein (HPASMC lysate) was estimated and divided to obtain the amount (pg) per μg total protein. Three independent experiments (biological replicates) were performed in triplicate.

***In vitro* kinase activity assay using recombinant cyclin D1-CDK4**

Recombinant cyclin D1-CDK4 GST-tagged protein complex (Invitrogen, #PV4400) was diluted to 5 ng/μL (40 nM) with 25 mM Tris-HCl, pH 7.4 and incubated with either 2 mM DTT (reduced), 50 μM H_2O_2 (oxidized) or H_2O (air-oxidized) at 30°C for 30 minutes. 10 mM $MgCl_2$, 0.5 μg (0.23 μM) full length Rb protein (Abcam, #ab83205), and/or 1 mM ATP was added and incubated at 30°C for an additional 10, 30 or 60 minutes. At each time point, a sample was taken and added to non-reducing sample buffer to end the assay. Cyclin D1-CDK4 disulfide formation and Rb phosphorylation were measured by immunoblotting.

Cyclin D1-CDK4 immunoprecipitation

WT cyclin D1-HA (pcDNA3 Addgene #11181) and WT or cysteine mutant CDK4-FLAG (pcDNA3.1-C-(k)-DYK GenScript OHu21245) were overexpressed in HPASMCs or HAP1 CDK4 KO cells for 24 hours using Lipofectamine 3000 or Lipofectamine 2000, respectively. After 24 hours, cells were washed with PBS and lysed in 300 μL ice-cold lysis buffer (50 mM Tris-HCl pH 7.4, 150 mM NaCl, 1% Triton X-100) supplemented with cOmplete^{TM} EDTA-free protease inhibitor tablets (Roche, #11836170001, 1 tablet per 10 mL). Cell lysates were collected using a cell scraper and maintained on ice for 5 minutes with frequent mixing using a vortex. The lysate was cleared by centrifugation at 14000 g for 5 minutes at 4°C. 50 μL cell supernatant was added to equal volumes of non-reducing sample buffer, this was termed 'input'. Meanwhile, anti-FLAG agarose beads (Merck, #A2220), used at 30 μL/sample, were equilibrated with excess lysis buffer by centrifugation at 8200 g for 30 seconds. Beads were resuspended in lysis buffer. 200 μL of cell supernatant was transferred into an Eppendorf test tube containing 30 μL of equilibrated beads and rotated at 18 RPM for 2 hours at 4°C. Beads were collected by centrifugation at 1000 g for 1 minute. The beads-containing pellet was placed on ice and washed three times with 500 μL wash buffer (50 mM Tris-HCl pH 7.4, 300 mM NaCl, 1% Tween-20). The pellet was resuspended in 50 μL non-reducing sample buffer and heated at 65°C for 5 minutes, this was termed 'IP'. All samples were centrifuged at 1000 g for 1 minute before separation of the supernatant by SDS-PAGE and analysis by non-reducing or reducing immunoblotting.

***In vitro* kinase activity assay of immunoprecipitated cyclin D1-CDK4**

Cyclin D1-HA (pcDNA3 Addgene #11181) and CDK4-FLAG (pcDNA3.1-C-(k)-DYK GenScript OHu21245) were co-immunoprecipitated under non-reducing conditions using anti-FLAG agarose beads, as

described above. After incubation of anti-FLAG agarose beads and cell supernatant for 2 hours, beads were washed three times with wash buffer. The beads-containing pellet was then washed once in kinase assay buffer (25 mM Tris-HCl pH 7.4 and 10 mM $MgCl_2$). Each sample was then resuspended in 95 μL of kinase assay buffer supplemented with 2 mM DTT (reduced), 1 μM H_2O_2 (oxidized), or H_2O (air-oxidized), and incubated for 30 minutes at 30°C and 400 RPM. Subsequently, 1 μM (4.3 μg) Rb GST-tagged fragment (Merck, #SRP0256) and/or 1 mM ATP was added, along with 1 μM, palbociclib (LKT laboratories, #P0344), where required, to a final volume of 100 μL. Each experimental condition was incubated for 30 or 60 minutes at 30°C, 400 RPM. Non-reducing sample buffer was added to end the assay, and samples were heated at 65°C for 5 minutes. Samples were analyzed by non-reducing or reducing immunoblotting.

The effect of alkylation on recombinant cyclin D1-CDK4 kinase activity

Recombinant cyclin D1-CDK4 GST-tagged protein complex (Invitrogen, #PV4400) was diluted to 50 ng/μL with 25 mM Tris-HCl pH 7.4. 250 ng (40 nM) cyclin D1-CDK4 protein, 25 mM Tris pH 7.4 and 2 mM DTT were incubated at 30°C for 30 minutes to reduce the protein. 10 mM *N*-ethylmaleimide (NEM) or 10 mM maleimide (Mal) were added from an 85 mM stock diluted in ultrapure H_2O, and incubated for a further 2 hours at 30°C. Following this, 1 mM ATP, 10 mM $MgCl_2$ and 2.13 μg (1 μM) Rb GST-tagged fragment (Merck, #SRP0256) were added to a final volume of 50 μL. After incubation for 30 or 60 minutes at 30°C reducing sample buffer containing 5% β-mercaptoethanol was added to stop the assay.

Liquid Chromatography tandem mass spectrometry

HeLa cell treatment and co-immunoprecipitation:

$9x10^6$ HeLa cells seeded in twelve 15cm plates, each overexpressed with 3 μg WT CDK4-FLAG and 6 μg WT cyclin D3-HA plasmid DNA for 24 hours. The plasmid DNA, 30μL/15cm plate, and lipofectamine 2000 were diluted in opti-MEM reduced serum medium and incubated at room temperature for 15 minutes before addition to cells. After 24 hours, cells were treated for 15 minutes with or without H_2O_2 and lysed in 2 mL ice-cold lysis buffer (50 mM Tris-HCl pH 7.4, 150 mM NaCl, 1% Triton X-100) supplemented with cOmplete™ EDTA-free protease inhibitor tablets. Cell lysates were collected, cleared by centrifugation at 14,000g for 5 minutes at 4°C, and incubated with FLAG-agarose beads under non-reducing conditions for 2 hours at 4°C. The co-immunoprecipitated sample was separated by SDS-PAGE under non-reducing conditions (H_2O_2-treated) or reducing conditions (5% β-mercaptoethanol) and stained overnight with Colloidal Coomassie stain. A band at ~75kDa was excised and analysed by liquid chromatography tandem mass spectrometry, firstly for protein identification, and secondly for presence of a peptide corresponding to the cyclin D3-CDK4 disulfide.

Identification of proteins in a ~75kDa band by LC-MS/MS:

The 75kDa band was excised, reduced with 10mM DTT at 56°C for 30 minutes, and alkylated with 55mM iodoacetamide. Enzymatic digestion was performed by trypsinization for 16 hours. The peptide samples were resuspended in 20 μL of MS sample buffer (2% acetonitrile in 0.05% formic acid), 6 μL of which was injected to be analyzed by LC-MS/MS. Chromatographic separation was performed using a U3000 UHPLC NanoLC system (ThermoFisherScientific, UK). Peptides were resolved by reversed phase chromatography on a 75 μm C18 Pepmap column (50 cm length) using a three-step linear gradient of 80% acetonitrile in 0.1% formic acid. The gradient was delivered to elute the peptides at a flow rate of 250 nL/min over 60 minutes starting at 5% B (0-5 minutes) and increasing solvent to 40% B (5-40 minutes) prior to a wash step at 99% B (40-45 minutes) followed by an equilibration step at

5% B (45-60 minutes). The eluate was ionised by electrospray ionisation using an Orbitrap Fusion Lumos (ThermoFisherScientific, UK) operating under Xcalibur v4.3. The instrument was first programmed to acquire using an Orbitrap-Ion Trap method by defining a 3s cycle time between a full MS scan and MS/MS fragmentation by collision induced dissociation. Orbitrap spectra (FTMS1) were collected at a resolution of 120,000 over a scan range of *m/z* 375-1800 with an automatic gain control (AGC) setting of $4x10^5$ (100%) with a maximum injection time of 35 ms. Monoisotopic precursor ions were filtered using charge state (+2 to +7) with an intensity threshold set between $5x10^3$ to $1x10^{20}$ and a dynamic exclusion window of 35s ± 10 ppm. MS2 precursor ions were isolated in the quadrupole set to a mass width filter of 1.6 *m/z*. Ion trap fragmentation spectra (ITMS2) were collected with an AGC target setting of $1x10^4$ (100%) with a maximum injection time of 35 ms with CID collision energy set at 35%.
Raw mass spectrometry data were processed into peak list files using Proteome Discoverer (ThermoScientific; v2.5). The raw data file was processed and searched using the Sequest [95] search algorithms against the Uniprot Human Taxonomy (50,727 entries). All database searching was performed at a stringency of 1% FDR including a decoy database search. The database output file was uploaded into Scaffold software (version 5.1.2) for visualisation and manual verification.

Identification of disulfide cyclin D3-CDK4:

The 75kDa band was excised, and enzymatically digested using trypsin for 16 hours, without prior reduction or alkylation. The peptide samples were resuspended in 20 μL of MS sample buffer, 6 μL of which was injected to be analyzed by LC-MS/MS, as described above.
Raw spectra were processed into peak list files and searched against a bespoke database containing only the sequence files for the two proteins of interest, i.e., CDK4 and cyclin D3 (P11802 and P30281), as described above, only with changes to the selection of modifications. Carbamidomethylation was removed from the parameter list while dioxidation and trioxidation on cysteine residues were included (**Table 2**). Acetylation (N-terminal) and oxidation (methionine) were unchanged.
The searched data were uploaded into Scaffold software for interrogation of the assigned peptides and fragmentation spectra. The raw Base Peak MS spectra was used for identification of a combined peptide with predicted mass that corresponds to disulfide cyclin D3-CDK4. The predicted mass of the combined peptide **(Figure S7C)** was calculated with the inclusion of possible modifications such as N-terminal acetylation and free cysteine dioxidation or trioxidation, prior to *in-silico* digest using the MS-Digest function in Protein Prospector, results are shown in **Table 2**. The mass-to-charge ratios (*m/z*) were used to search the raw data for evidence of MS precursor peaks that matched correctly. This was performed in Xcalibur v4.3 allowing for a short range of masses +/- 1 Da either side of the selected peptide *m/z*. All extracted peaks from the MS survey scan were manually inspected for the correct mass of the precursor ion and charge state (+/- 0.6 Da from predicted *m/z*).
The mass spectrometry proteomics data have been deposited to the ProteomeXchange Consortium via the PRIDE [96] partner repository with the dataset identifier PXD035743 and 10.6019/PXD035743.

Modification	Molecular weight	m/z
Non-modified	2649.2367	
Acetyl+dioxidation	2723.2367	681.3092^{4+}; 545.1473^{5+}
Acetyl+trioxidation	2739.2367	685.3092^{4+}; 548.3473^{5+}
Met-loss+acetyl+dioxidation	2624.1966	656.5492^{4+}; 525.3393^{5+}
Met-loss+acetyl+trioxidation	2640.1966	660.5491^{4+}; 528.53932^{5+}

Table 2. The predicted mass of a combined peptide between CDK4 C135 and cyclin D3 C5/6 was calculated with the inclusion of possible modifications prior to *in-silico* digest using the MS-Digest function in Protein Prospector.

Cell cycle analysis by flow cytometry

HPASMCs were seeded at $1x10^5$ cells/mL in 6-well plates in starvation media (0.1% FBS) and incubated for 40 hours to induce G0/1 phase synchronization. Two additional wells were plated in growth media, these cells were termed 'cycling cells'. After 40 hours, the two wells of 'cycling cells' and one well of 'G0/1 synchronized cells', were harvested. The remaining cells were stimulated to enter the cell cycle using growth media (10% FBS), supplemented with or without 50 μM H_2O_2 or 10 μM palbociclib (diluted in DMSO), for 8, 16, 24, or 28 hours. At each time point, cells were detached with trypsin and pelleted at 500 g for 5 minutes. The supernatant was discarded, and the cell pellet was washed with PBS. Cells were then fixed using 400 μL ice-cold PBS, into which 800 μL ice-cold ethanol was added dropwise whilst cells were held on a slowly rotating vortex. The fixed cells were stored at 4°C for up to one week until analysis by flow cytometry.

On the day of analysis, fixed cells were warmed to room temperature and pelleted at 500 g for 5 minutes. The cell pellet was washed once with PBS and then resuspended in 190 μL 1x RNase solution (Abcam, #ab139418) diluted in PBS, and incubated for 5 minutes at 37°C. 10 μL (50 μg/mL) propidium iodide (Abcam #ab139418) was then added for a further 25 minutes at 37°C. Cells were stored on ice in the dark until analysis was performed at the NIHR GSTT BRC Flow Cytometry Platform at Guy's Hospital. Propidium iodide-stained cells were analyzed using the FACSCANTO™II (BD Biosciences) and data were analyzed using FlowJo™ Software version 10.0.7 (BD Biosciences). A 488 nm excitation laser, set for PerCPCy5.5, was used to excite the propidium iodide. Samples were acquired at a low flow rate to encourage single cells to pass through the laser. The gating strategy was set up to exclude cell doublets and a threshold of 5000 was set to all gates to exclude debris, after this, no changes to voltages or gating were made. The number of cells in each cell cycle phase was measured using the Watson Pragmatic cell cycle model in FlowJo™. The following constraints were added to this model: the G1 peak range was set between 35K-60K on the PerCP intensity scale, the G2 peak was set at 1.90-1.95x G1 peak, and G1 and G2 have equal coefficients of variation.

Proliferation measurements using the xCELLigence RTCA system

Proliferation of HPASMCs, HAP1 or MCF7 cells was assessed by electrical impedance using xCELLigence real-time cell analysis (RTCA, Agilent) in a humid incubator at 37°C and 5% CO_2. HPASMCs were seeded in a 16-well E-plate at $3x10^4$ cells/well in starvation media (0.1% FBS) for 40 hours, before growth media (10% FBS) was added to stimulate proliferation in the absence or presence of 50 μM H_2O_2. Cell index was recorded every 15 minutes and the data were normalized to a cell index value of zero at treatment time. HAP1 cells were seeded at $2x10^4$ cells/well in IMDM supplemented with 10% FBS. Cell index was measured for 72 hours from the time of seeding. MCF7 cells were seeded at $3x10^4$ cells/well in DMEM supplemented with 10% FBS. After 24 hours, H_2O_2, palbociclib or auranofin treatment was

added for an additional 72 hours. Data were normalized to cell index at the time of treatment. Area under the curve was employed to present and compare proliferation rate between experimental groups.

Generation of WT and C135A HAP1 stable cell lines

A mammalian expression lentiviral vector encoding human WT CDK4, pLV-PuroCMV-hCDK4, was purchased from GenScript Biotech. Using the Q5 mutagenesis kit (New England Biolabs), CDK4 C135 was mutated to alanine (C135A) using the primers described in Table 1, according to manufacturers' instructions. HEK293FT cells were seeded at $4x10^5$ cells/mL in 2x 10 cm cell culture dishes in Gibco™ DMEM supplemented with 10% FBS. After 24 hours, each plate was transfected with 3rd generation lentiviral plasmids (2.83 μg pLP1, 1.33 μg pLP2, 1.84 μg pLP/VSVG), along with 5 μg WT or C135A CDK4 expression plasmid. 16 hours later, media was replenished with IMDM supplemented with 10% FBS and 1% Penicillin/Streptomycin. 24 hours later, the viral particle-containing media was collected and filtered using a 0.45 μm filter. The viral particles were immediately added to HAP1 CDK4 KO cells in a 2-fold serial dilution along with 8 μg/mL polybrene. After 24 hours, media was replenished, and cells were returned to the incubator for 3 days. Stably transduced cells were selected using 2 μg/mL puromycin for 3 days.

Genotyping of C135A CDK4 knock-in mice

Genomic DNA was extracted and amplified from mouse ear clips using a Rapid Extract PCR kit (PCR biosystems), the primers indicated in **Figure S14**, and the following thermocycler protocol: 95°C 5 minutes, 35 cycles [95°C 30 seconds, 60°C 30 seconds, 72°C 1 minute], 72°C 10 minutes. PCR products underwent MfeI restriction digest (5 enzyme units /10μl PCR product) at 37°C in buffer G (Thermo Scientific) for at least 1 hour. Mice were genotyped according to the presence of a single 510bp band (wildtype, WT), a 374bp and 136bp band (knock-in, KI), or all three bands (heterozygous) when resulting fragments were separated on a 2% agarose gel in TAE buffer (**Figure S14B**).

Isolation and culture of mouse pulmonary artery smooth muscle cells

Mouse pulmonary artery smooth muscle cells (PASMCs) were isolated by a method adapted from Lee et al [97]. 10 weeks old male WT and CDK4 KI mice were terminally anaesthetized with a single intraperitoneal injection of sodium pentobarbital (300 mg/kg, Pentoject, Animalcare) and heparin (150 U) and dissected to expose the chest cavity. The pulmonary vasculature was slowly perfused with 3-5ml sterile PBS at room temperature *via* the right ventricle using a 25G needle, followed by 3-5ml 37°C 0.5% agarose, 0.5% iron particles, 1% penicillin-streptomycin and 1% amphotericin B in serum-free M199. Lungs were next inflated by infusion of 1% agarose, 1% penicillin-streptomycin and 1% amphotericin B in serum-free M199 *via* a 24G angiocatheter placed into the trachea. The heart-lung bloc was removed and submerged in ice cold PBS to solidify agarose before mincing of lung tissue. Iron-containing pulmonary vessels within the lung tissue were magnetically separated and washed three times with sterile PBS before collagenase (125U/mg) digestion for 1 hour at 37°C in a humidified incubator. The resulting slurry was triturated with an 18G needle. Iron-containing vessels were magnetically separated and washed three times with M199 containing 20% FBS, 1% penicillin-streptomycin and 1% amphotericin B (media), then resuspended in 3ml media and placed in a 35mm plate overnight. Magnetic separation of iron-containing vessels, washes and resuspension were repeated on day 2 (dish 1), day 6 (dish 2) and day 9 (dish 3) after isolation to ensure maximal migration of PASMCs from the wall of the vessels onto the cell culture plates. On day 13, PASMCs from the three 35mm dishes were washed with PBS, trypsinised and resuspended in M199 containing 20% FBS, 1%

penicillin-streptomycin and 1% amphotericin B. Iron particles were magnetically separated and discarded. PASMCs were pooled together and cultured to confluence in a T25 flask at 37°C with 5% CO_2 in a humidified incubator. For proliferation measurements using the xCELLigence RTCA system (as described above), passage 2 PASMCs were seeded at 3000 or 8000 cells/well (in duplicate or triplicate) in a 16-well E-plate.

Isolation and culture of mouse lung endothelial cells (MLEC)

Lungs were harvested from 6-7 weeks old male WT and CDK4 KI mice and pooled from two mice for each isolation. Lungs were washed twice with 37°C PBS containing 1% penicillin-streptomycin before finely mincing with scissors and a scalpel. Minced lung tissue was digested in 1mg/ml collagenase-dispase (Merck, #10269638001) in Dulbecco's Modified Eagle Medium (DMEM) for 45 minutes at 37°C with continuous shaking and triturated with an 18-gauge needle before passing through a 70μm nylon strainer, followed by a 30μm nylon strainer. The cell suspension was centrifuged at 350 x g for 10 minutes, the supernatant discarded, and the resulting cell pellet was resuspended in 5ml PBS before a further 5-minute centrifugation at 1300 RPM. The supernatant was removed, and the cell pellet was resuspended in 90μl of 4°C MACS buffer (1:20 dilution of MACS BSA stock solution Miltenyi, #130-091-376 in autoMACS rinsing solution Miltenyi, #130-091-222). 10μl of anti CD31 antibody-conjugated microbeads (Miltenyi, #130-097-418) were added and incubated at 4°C for 15 minutes. CD31+ cells were magnetically separated by passing the suspension through an LS column inside a MACS separator (Miltenyi #130-042-401) and washing thrice with 4°C MACS buffer. The LS column was removed from the MACS separator and CD31+ cells were flushed from the column in 5ml MACS buffer and centrifuged at 1300 RPM for 5 minutes. CD31+ cells were resuspended in DMEM media containing 20% FBS, 1X endothelial cell growth supplements (Merck, #211F-GS), 100μg/ml heparin (Merck, #H3149-100KU) and 1% penicillin-streptomycin and plated onto a 12 well plate well pre-coated with 0.1% fibronectin (Sigma, #F1141) and 0.4% gelatin (Merck, #G1393). MLEC were maintained at 37°C with 5% CO_2 in a humidified incubator. Media was replaced the following day and cells were passaged when 90% confluent. Cells displayed the 'cobblestone' morphology characteristic of cultured endothelial cells and stained positive for VE-cadherin. For proliferation measurements using the xCELLigence RTCA system (as described above), passage 2 MLEC were seeded at 2500 or 5000 cells/well (in duplicate or triplicate) in a 16-well E-plate pre-coated with 0.1% fibronectin and 0.4% gelatin.

Hypoxia-induced model of pulmonary hypertension

Hypoxic PH was induced by housing 10-12 weeks old C57BL/6J male mice obtained from Charles River Laboratories (UK) in a chamber (A-chamber, Biospherix Ltd.) containing normobaric hypoxia (10% oxygen) for 14 days. Oxygen was displaced by 100% nitrogen and was regulated with the dual redundant ProOx P360 oxygen probe (Biospherix Ltd.). The carbon dioxide concentration remained unchanged and was monitored using a carbon dioxide monitor and regulated using soda lime pellets (Merck, #72073). Control mice were exposed to normal air (normoxia, 21% oxygen) for the same duration of time. Throughout experiments, fresh cages, water, and food were provided every 7-10 days, during which the chamber remained ventilated with fresh air for a short period of time. Mice were randomized and treated with auranofin or vehicle by osmotic minipump, up to 24 hours before subjecting them to hypoxia and throughout the whole duration of hypoxic episode. Briefly, mice were surgically implanted with subcutaneous osmotic mini-pumps (Alzet, model 2002, 0.5 μL per hour, 200 μL) for continuous delivery of auranofin (8.5 mg/kg/day) (Enzo, #BMLEI206) for 14 days during the induction of hypoxic PH. The day before surgery, minipumps were filled with auranofin or vehicle (60%

DMSO/saline) and primed overnight at 37°C under sterile conditions. The surgery was performed in mice anaesthetized with 2% isoflurane (Centaur Services) in 0.5 L of oxygen/minute with perioperative analgesia (1 mg/kg methadone, Comfortan, Dechra, UK).

Sugen (SU5146)/hypoxia-induced model of pulmonary hypertension

Wild type (WT) or 'redox-dead' CDK4 C135A knock-in (KI) adult 8-12 weeks old male or female mice were subjected to hypoxia (10% oxygen) for 3 weeks and subcutaneously injected with the VEGF receptor antagonist Sugen (SU5146, 20mg/kg, Abcam) at the start of each week. SU5416 was suspended in 0.5% (w/v) carboxymethylcellulose sodium, 0.9% (w/v) sodium chloride, 0.4% (v/v) polysorbate 80, 0.9% (v/v) benzyl alcohol in ultrapure water, as reported previously [98]. The whole study was performed in two randomized cohorts. At the end of the Sugen (SU5146)/hypoxia protocol, all mice were subjected to echocardiographic and hemodynamic assessment followed by RV:LV+septum ratio measurements and (with the technical exclusion of some unsuitable lungs, as explained below) a histological assessment of pulmonary vascular remodeling, as described below. The left lung lobe was isolated from the second cohort of mice, or from rats at the end of the experimental protocol. Venous blood taken from inferior vena cava after hemodynamic measurements from some WT or KI mice from Sugen/hypoxia group was analysed with a hand-held iSTAT analyser using CG8+ cartridges (Abbott Laboratories).

In an intervention study, 10-12 weeks old C57BL/6J male mice, obtained from Charles River Laboratories (UK), were subjected to hypoxia (10% oxygen) for 3 weeks and subcutaneously injected with the VEGF receptor antagonist Sugen (SU5146, 20mg/kg, BioTechne) at the start of each week. SU5416 was resuspended as above. For the duration of the experiment, mice were treated with daily intraperitoneal injections of 10 mg/kg auranofin diluted in 10% DMSO/saline, or vehicle (10% DMSO/saline). This experiment was performed in two cohorts. At the end of the Sugen (SU5146)/hypoxia protocol, both cohorts were subjected to hemodynamic assessment and RV:LV+septum ratio measurements, while only the second cohort was subjected to histological assessment of pulmonary vascular remodeling, as described below.

Alternatively, 10-12 weeks old Wistar-Kyoto male rats obtained from Charles River Laboratories (UK) were subcutaneously injected on day 0 with Sugen (SU5416, 20 mg/kg, BioTechne) before exposure to hypoxia (10% oxygen) for 3 weeks. Rats were then returned to normoxia (21% oxygen) and treated with daily intraperitoneal injections of 8 mg/kg Auranofin (Aur) or Vehicle (Veh, 10% saline/DMSO). Rats underwent non-invasive imaging assessment by echocardiography at day 21 and combined echocardiography and hemodynamic assessment under terminal anesthesia [24] at day 35, after which lungs were fixed for immunohistochemical staining.

Echocardiography

Non-invasive transthoracic echocardiography was performed with a Vevo 3100 or Vevo F2 (VisualSonics, Toronto, ON, Canada) system equipped with a 10-22 MHz transducer (MX201 for Vevo 3100 or UHF46X for Vevo F2) to assess cardiac function and pulmonary arterial stiffness in anaesthetized mice or rats. Briefly, Wistar-Kyoto rats were anaesthetized with 2% isoflurane in 1 L of oxygen per minute in a supine position and with all legs taped to ECG electrodes for heart rate monitoring. The core body temperature was maintained at 36.5-37°C with a rectal body temperature probe. High-resolution, two-dimensional B-mode and M-mode images were obtained at the level of the papillary muscles and further analyzed offline with VevoLab software (VisualSonics), as before [24]. Pulsed-wave Doppler probe and B-mode images were employed to assess pulmonary arterial flow parameters distally of pulmonary trunk bifurcation. The estimated pulmonary vascular resistance

index was calculated by dividing right ventricular systolic pressure (measured as described below) by cardiac output assessed by echocardiography.

Hemodynamic assessment

Animals were anesthetized, as described above, and maintained at 36.5-37°C with a rectal temperature probe feedback-regulated heating table. Ventilation was induced with a small rodent ventilator at 140 breaths per minute. The chest was then opened, and the right ventricle was punctured with a 25 G needle (Terumo, #NN2516R). A pre-calibrated pressure catheter (1.2 French), connected to the ADVantage ADV500 system (SciSense Inc) was inserted, and right ventricular systolic pressure (RVSP) and heart rate were recorded. The reading used for analysis was taken after at least 15 minutes of stable recording at 36.5-37°C. One WT SuHX female mouse was excluded from RVSP and heart rate measurements due to unsuccessful intubation. Immediately after hemodynamic measurements were completed, the left lung was perfusion fixed via the trachea with 4% (v/v) formalin/PBS by inflation to 20 cm of H_2O. Only successfully inflated lungs through fixation by perfusion with 4% formalin were included in the study. In the CDK C135A KI mouse study (**Figure 5**), seven lungs were excluded from normoxic groups, and one lung was excluded from the Wt SuHx group. The lungs were then processed into paraffin blocks which were later sectioned. After completion of the RVSP measurements and lung perfusion, the RV:LV+septum ratio was examined postmortem by dissecting the right ventricle and left ventricle plus septum and weighing them separately using scales.

Measurements of the cyclin D-CDK4 disulfide in mouse pulmonary arteries

Where stated, mice were treated for 6 hours with 10 mg/kg auranofin diluted in 10% DMSO/saline, or vehicle (10% DMSO/saline), which were administered through a single intraperitoneal injection. Alternatively, mice were subjected to normobaric hypoxia for 3 days (10% oxygen) or normoxia (21% oxygen), as described above. Mice were terminally anaesthetized with a single intraperitoneal injection of sodium pentobarbital (300 mg/kg, Pentoject, Animalcare) and heparin (150 U). Once unconscious and the hind leg pinch reflex was lost, both the heart and lungs were isolated. Under a microscope the pulmonary trunk together with the right and left main pulmonary arteries were cleaned by removing fat and surrounding tissue in an ice-cold saline solution (Sterets Normasol). Samples were snap-frozen in liquid nitrogen. Homogenization was performed by instant crushing of the whole vessel sample in liquid nitrogen using a pre-cooled custom-made metal mortar and pestle, followed by the addition of non-reducing sample buffer.

Immunohistochemical staining

Paraffin embedded sections (5 µm) of lung from WT and CDK4 KI mice were processed for immunohistochemical staining for α-smooth muscle actin (α-SMA) in Mauspathologie Facility, Institute of Neuropathology of University Medical Centre Hamburg-Eppendorf, as following. After dewaxing and inactivation of endogenous peroxidases (3% hydrogen peroxide), antibody specific antigen retrieval was performed using the Ventana Benchmark XT machine (Ventana, Tuscon, Arizona, USA). Sections were blocked and afterwards incubated with the anti-SMA antibody (DAKO #M0851; 1:100). Specific anti-mouse N-Histofine® MOUSESTAIN KIT (#414322F; Nichirei Biosciences, Wedel, Germany) was used as secondary antibody. Detection of secondary antibodies and counter staining were performed with the ultraview universal DAB detection kit from Ventana (#760-500; Ventana, Tuscon, Arizona, USA) and mounted in mounting media. Stained lung sections were scanned with a NanoZoomer 2.0-HT digital slide scanner (Hamamatsu). For this, 8 to 10 focus points were chosen

manually per individual lung section to ensure a focused scan of the entire cross section of each lung. Scans were performed at a 40x magnification with 0,23 µm/Pixel using the Hamamatsu software NDP.view 2.3.

Paraffin embedded sections (5 µm) of lung from the rest of the animals were processed for immunohistochemical staining for α-SMA in King's College London as following. Deparaffinized sections were boiled for 20 min in 10 mM citrate buffer pH 6.0, for antigen retrieval. Primary antibody against α-SMA (α-smooth muscle actin, DAKO Clone 1A4, #M0851; 1:100), or negative control mouse IgG2a (DAKO, #X0943; 1:100) was diluted in horse serum (VECTASTAIN Elite ABC Kit, PK-6102, Vector Laboratories) in PBS. Sections were then incubated with primary antibody (1:100) overnight at 4°C. Biotinylated anti-mouse secondary antibody (VECTASTAIN Elite ABC kit, PK-6102, Vector Laboratories) was incubated for 1 hour at room temperature and α-SMA signal was developed using the 3,3 diaminobenzidine as the substrate for color in the peroxidase reaction (DAB, SK-4100, Vector Laboratories). Stained lung sections were scanned with a NanoZoomer S360 digital slide scanner (Hamamatsu). For this, 8 to 10 focus points were chosen manually per individual lung section to ensure a focused scan of the entire cross section of each lung. Scans were performed at a 40x magnification with 0,23 µm/Pixel using the Hamamatsu software NDP.view 2.3.

For all lungs, pulmonary vascular remodeling was quantified by assessing the percentage of non-muscularized, partially muscularized and fully muscularized pulmonary vessels of diameter between 10 and 100 µm using the Hamamatsu software NDP.view 2. Each vessel was classified as either non-muscularized (up to 25% α-SMA around the vessel), partially muscularized (25% to 75% α-SMA around the vessel) or fully muscularized (≥75% α-SMA around the vessel). The percentage of pulmonary vessels in each muscularization category was determined by dividing the number of vessels in that category by the total number counted in the same experimental group. The analysis was done by an observer blinded to the treatment of the animals; on average, 243 vessels/mouse for WT or CDK4 KI mice, 147 vessels/mouse and 110 vessels/rat for the rest of the studies were analyzed.

Affymetrix GeneChip microarray

Affymetrix analysis was performed as described previously[24]. In brief, mice were subjected to normobaric hypoxia (10% oxygen) for 3 days. Mice were anaesthetized with a single intraperitoneal injection of sodium pentobarbital (300 mg/kg, Pentoject, Animalcare) and heparin (150 U), and left lung lobes were dissected, washed in saline, and snap frozen in liquid nitrogen. mRNA extraction was performed using the RNeasy Plus Universal kit (Qiagen). ~5 µg of SPIA-amplified complementary DNA was fragmented and biotin-labelled using the Encore Biotin module (NuGEN), and hybridization cocktails prepared as recommended by NuGEN. Samples were hybridized to Mouse Gene 2.0 ST arrays using standard protocols as recommended by the manufacturer (Affymetrix). Arrays were scanned using GCS3000 scanner and the resulting image intensity (CEL) files were processed using the RMA-sketch algorithm in Expression Console software (Affymetrix), to generate normalized, background-corrected, summarized data files (CHP format). All chips passed basic data quality control checks as suggested by Affymetrix. Qlucore Omics Explorer software was then employed to process the raw CHP files, prepare comparisons of the fold changes and p values using the anova algorithm, and present the data as the top 1000 genes/transcripts ranked by the p- or q-value. Pathway analysis was performed in R-studio using the gseGO function of the clusterProfiler package[99]. The ontology (ont) *biological processes* (BP) was used; the boundary to calculate the p-value (eps) was set to 1e-300, and the database *Mus musculus* (org.Mm.eg.db) was employed, while all other parameters were set at the default. Affymetrix data are deposited at Gene Expression Omnibus with accession number GSE244830 (https://www.ncbi.nlm.nih.gov/geo/query/acc.cgi).

Supplemental Figures

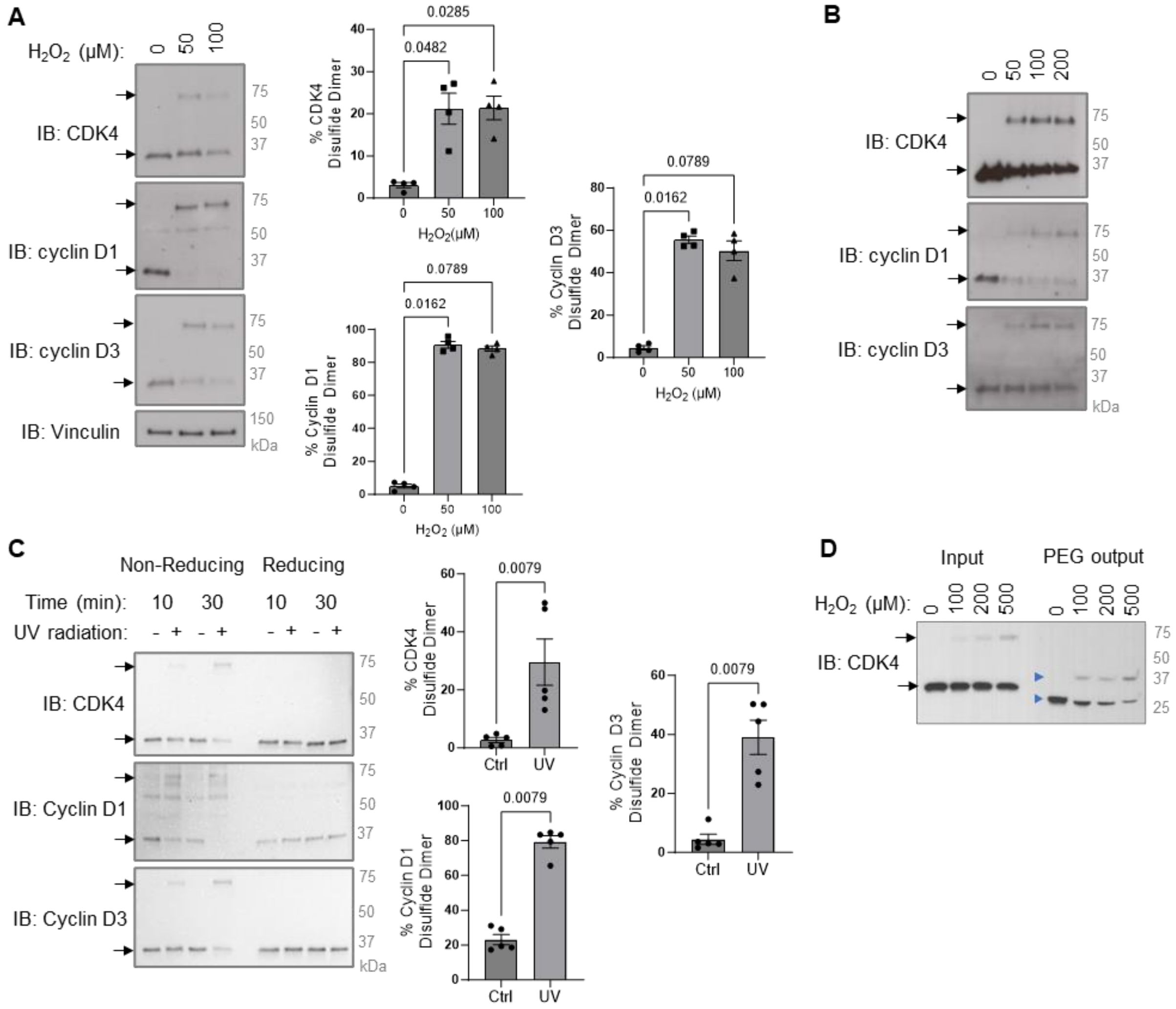

Figure S1. Cyclin D-CDK4 forms an oxidant-induced disulfide dimer in HPAECs and HPASMCs.
A) Cyclin D-CDK4 forms an oxidant-induced intermolecular disulfide bond in HPAECs treated with H_2O_2 in PBS. Monomeric and disulfide dimeric bands in cyclin D1, cyclin D3 and CDK4 are indicated by black arrows. Vinculin was used as a loading control. Graphs show the proportion of CDK4, cyclin D1 and cyclin D3 observed as a disulfide dimer. As the data sample size with an n<6 cannot be reliably tested for normality, *P* values are calculated using a non-parametric Kruskal-Wallis test followed by Dunn's multiple comparisons to compare H_2O_2-induced disulfide formation with 0 µM control (n=4 independent experiments), and the results are shown as means±SEM. **B)** Detection of the cyclin D-CDK4 disulfide bonds in HPAECs after treatment with H_2O_2 in Endothelial Cell Growth Medium-2. Non-reducing immunoblots are representative of n=3. **C)** UV-induced oxidation increased disulfide cyclin D-CDK4. Monomeric and disulfide dimer CDK4, cyclin D1 and cyclin D3 (indicated by black arrows) were detected by non-reducing immunoblotting of HPASMCs exposed to (+) or shielded from (-) a 365 nm UVA lamp for 10 minutes or 30 minutes. Graphs show quantification of the 30-minute time point. *P* values are calculated using unpaired two-tailed non-parametric Mann-Whitney test to compare UV-induced disulfide formation with 0 µM control (n=5 independent experiments), and the results are shown as means±SEM. Immunoblots are representative of the results that are the most similar to the mean value. **D)** CDK4 contains a redox-active cysteine residue as detected by a mobility shift following the PEG-switch assay of H_2O_2-treated HPASMCs. CDK4 monomer and disulfide (input) is shown by black arrows, while monomeric and PEGylated CDK4 (PEG-output) are shown by blue arrowheads (immunoblots are representative of the average result of n=3 independent experiments).

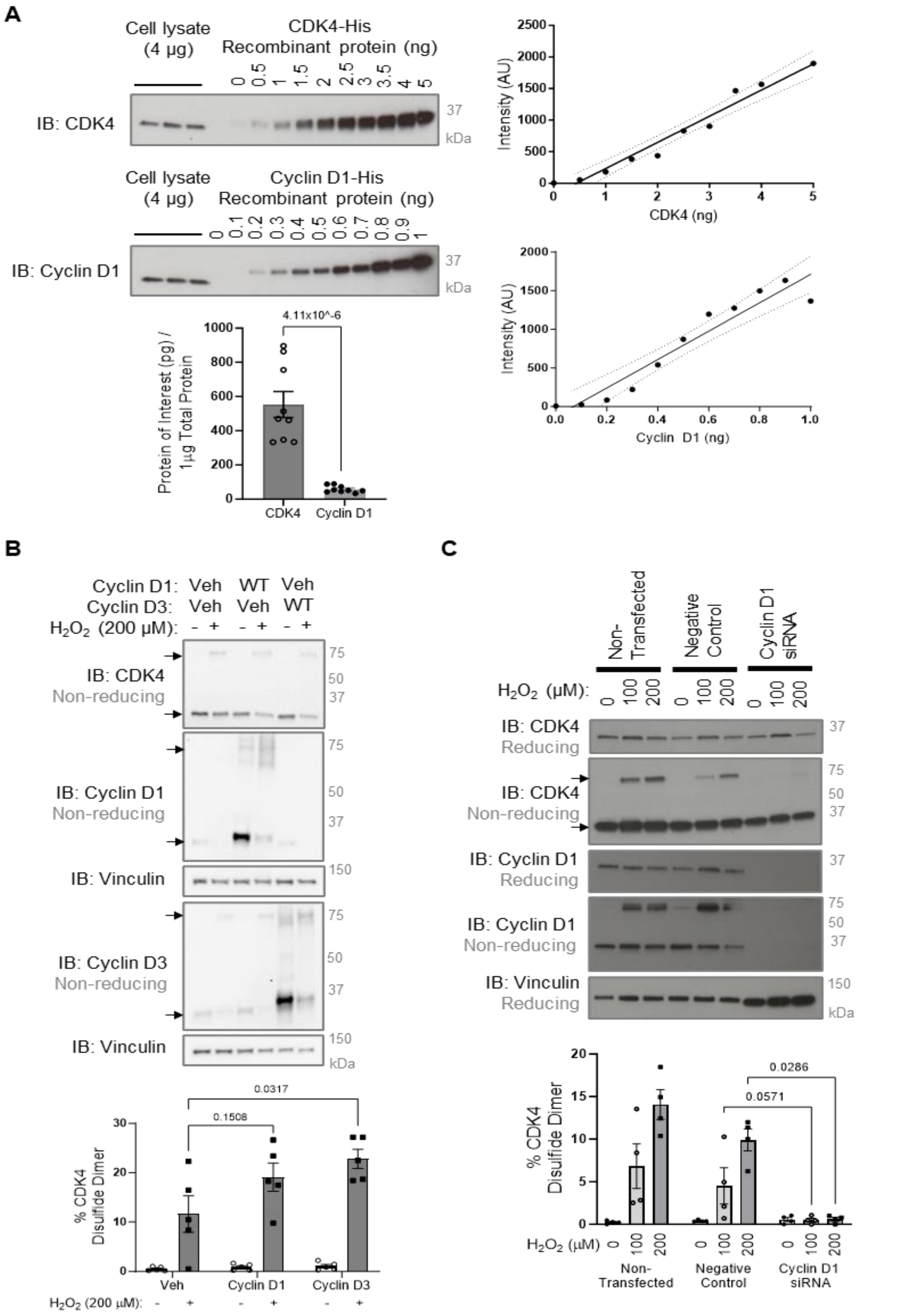

Figure S2. Cyclin D protein expression is limiting in disulfide formation

A) A comparison between the amount of cyclin D1 and CDK4 protein in HPASMCs shows CDK4 is in excess. Total protein concentration (µg/mL) in HPASMCs was measured using a BCA assay. 4 µg total

protein, alongside known concentrations of recombinant cyclin D1 or recombinant CDK4 protein, were immunoblotted under reducing conditions for cyclin D1 and CDK4, respectively. The signal intensities of immunoblots was measured by densitometry and was plotted. GraphPad Prism was used to fit a linear regression curve. The equation of the line was used to estimate the concentration of cyclin D1 and CDK4 in 4 µg HPASMC lysate. This was then divided to obtain the amount in pg/µg total protein. As the data sample size with an n<6 cannot be reliably tested for normality, a *P* value is calculated using an unpaired two-tailed non-parametric Mann-Whitney test to compare between two groups (n=3 independent experiments performed in technical triplicate). **B)** Cyclin D1/3 overexpression increased CDK4 disulfide dimer formation. Monomeric and disulfide dimeric cyclin D1, cyclin D3 and CDK4 were detected by non-reducing immunoblotting of HPASMCs overexpressed with cyclin D1 or cyclin D3 and treated with H_2O_2 for 15 minutes. The loading control, vinculin, was detected by immunoblotting under reducing conditions. *P* values are calculated using unpaired two-tailed non-parametric Mann-Whitney test to compare H_2O_2-induced disulfide formation between Cyclin D1 or Cyclin D3 overexpression and a Vehicle (n=5 independent experiments); the results are shown as means±SEM. **C)** Cyclin D1 protein knock down decreased CDK4 disulfide dimer formation. Monomeric and disulfide dimeric cyclin D1 and CDK4 was detected by immunoblotting of HPASMCs transfected with cyclin D1 siRNA or scrambled siRNA (negative control) for 48 hours. Cells were then treated with H_2O_2 for 15 minutes. *P* values are calculated using unpaired two-tailed non-parametric Mann-Whitney test to compare 100 µM or 200 µM H_2O_2-induced disulfide formation between Negative Control siRNA and Cyclin D1 siRNA treatment (n=4 independent experiments); the results are shown as means±SEM.

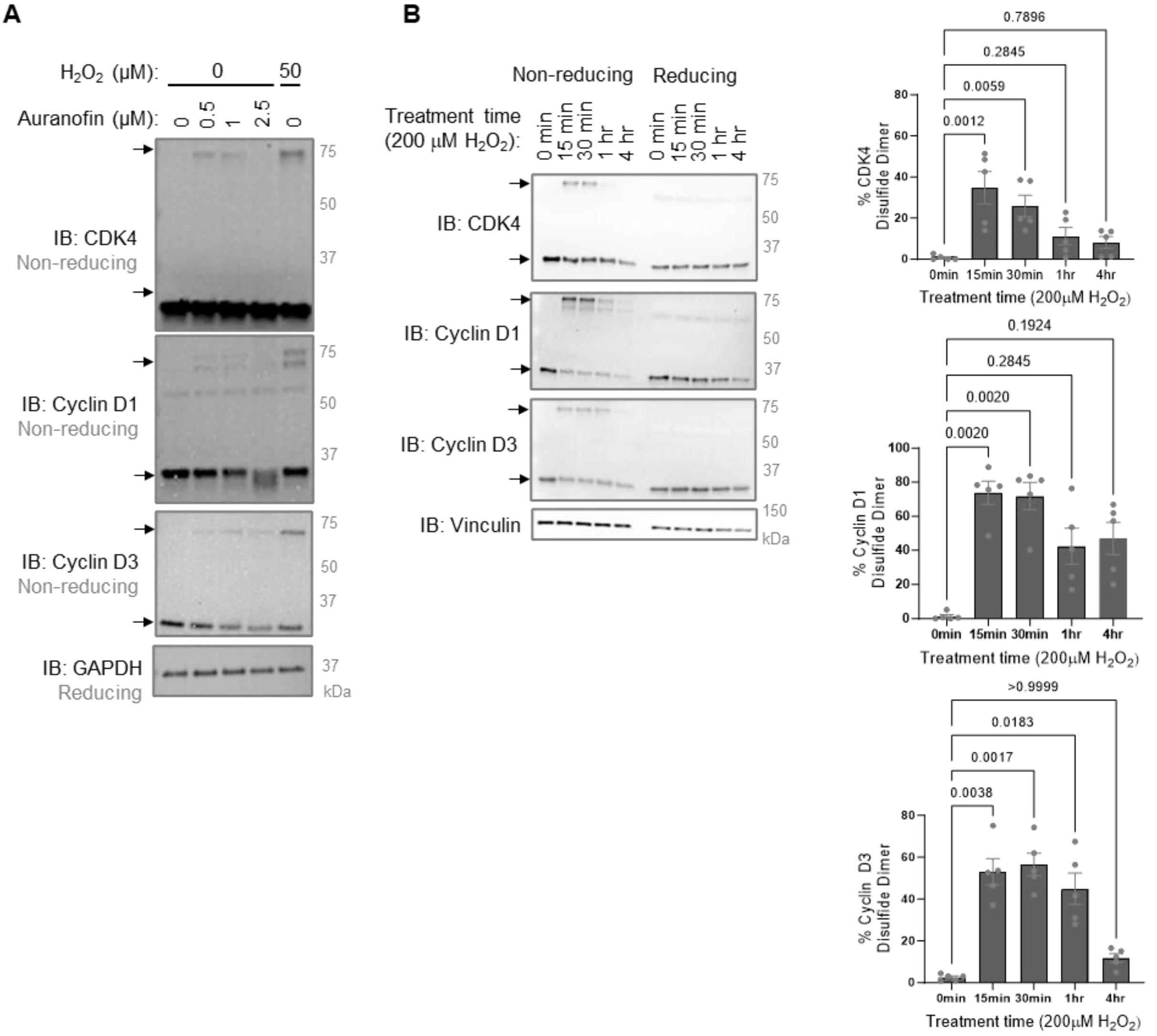

Figure S3. Reversibility of the cyclin D-CDK4 disulfide dimer

A) Auranofin treatment induced formation of disulfide cyclin D-CDK4. Monomeric and disulfide dimeric CDK4, cyclin D1 and cyclin D3 (indicated by black arrows) were detected by non-reducing immunoblotting of HPASMCs treated with auranofin or vehicle (0.05% DMSO) for a total of 1 hour 30 minutes in serum-free media. Where stated, H_2O_2 or vehicle was added for the last 15 minutes (immunoblots are representative of the average results, n=5 independent experiments). **B)** The cyclin D-CDK4 disulfide is reversible over time. Monomeric and disulfide dimeric CDK4, cyclin D1 and cyclin D3 were detected by immunoblotting of HPASMCs treated with 200 µM H_2O_2 for the stated duration. As the data sample size with an n<6 cannot be reliably tested for normality, *P* values are calculated using a non-parametric Kruskal-Wallis test followed by Dunn's multiple comparisons to compare H_2O_2-induced disulfide formation with 0 minutes control (n=5 independent experiments), and the results are shown as means±SEM.

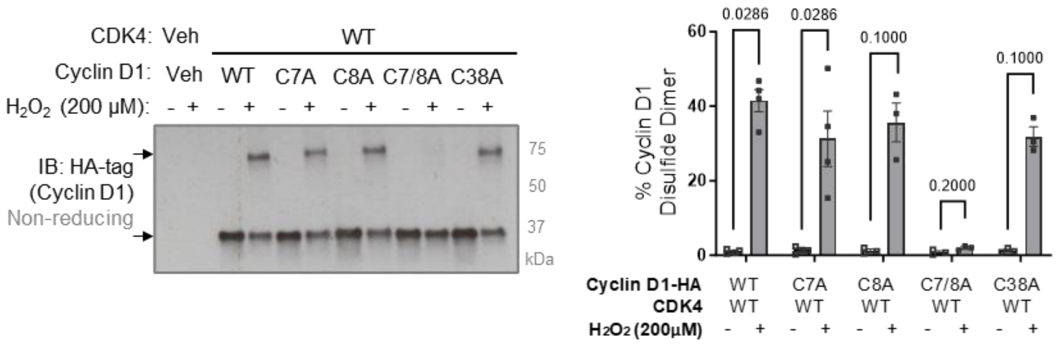

Figure S4. C7/8A cyclin D1 is redox-dead

Either cyclin D1 C7 or C8 can form the cyclin D-CDK4 disulfide bond. HPASMCs were transfected with WT or mutant cyclin D1-HA along with WT CDK4. After 24 hours, cells were treated with H_2O_2 for 15 minutes. Monomeric and disulfide dimeric cyclin D1 was detected by immunoblotting under non-reducing conditions using a HA-tag antibody. As the data sample size with an n<6 cannot be reliably tested for normality, *P* values are calculated using unpaired 2-tailed non-parametric Mann-Whitney test to compare between vehicle and H_2O_2 treatment for each mutant (WT/WT, C7A/WT, n=4; C8A/WT, C7/8A/WT, C38A/ WT, n=3), and the results are shown as means±SEM. Please note that for n=3, the only minimal achievable *P* value with the test used is 0.1000.

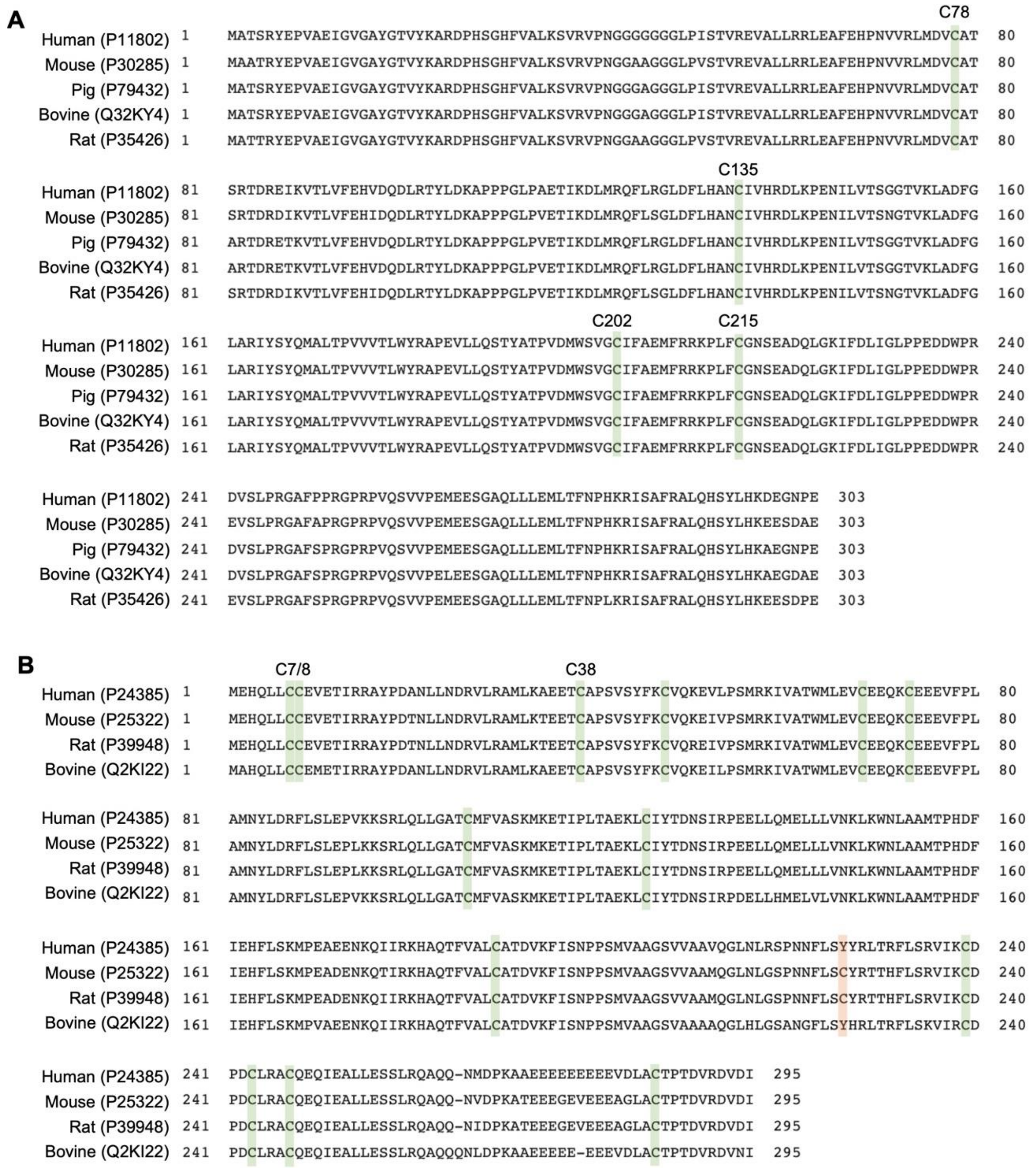

Figure S5. CDK4 and cyclin D1 sequence alignments

All cysteine residues in human CDK4 and human cyclin D1 are conserved between mammals. BLAST alignments of **A)** CDK4 mammalian sequences and **B)** cyclin D1 mammalian sequences. All sequences were obtained from the UniProt database with identification codes presented in brackets. Conserved cysteine residues are highlight in green, while non-conserved cysteine residues are highlighted in orange.

A

	Total Spectrum Count		
	H_2O_2-treated	Reduced	P-value
**CDK4**	340.5 +/- 73.5	17.8 +/- 4.1	0.0001
**CCND3**	184.8 +/- 43.9	12.3 +/- 3.8	0.0322

B

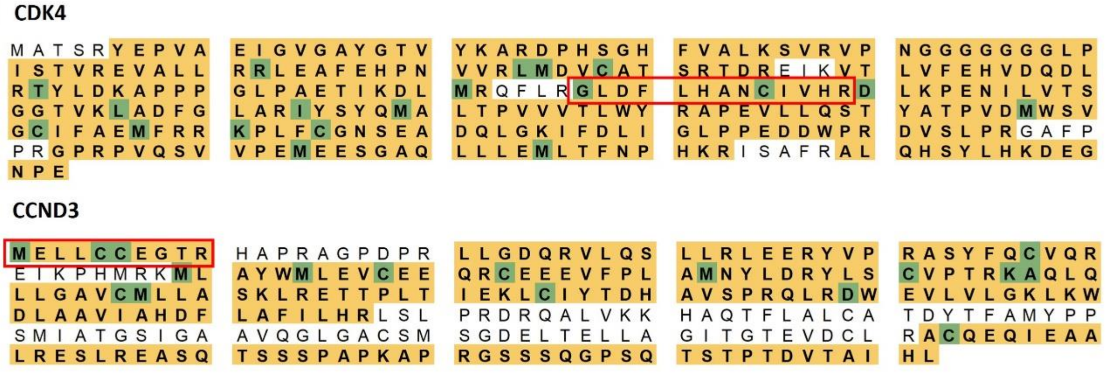

C

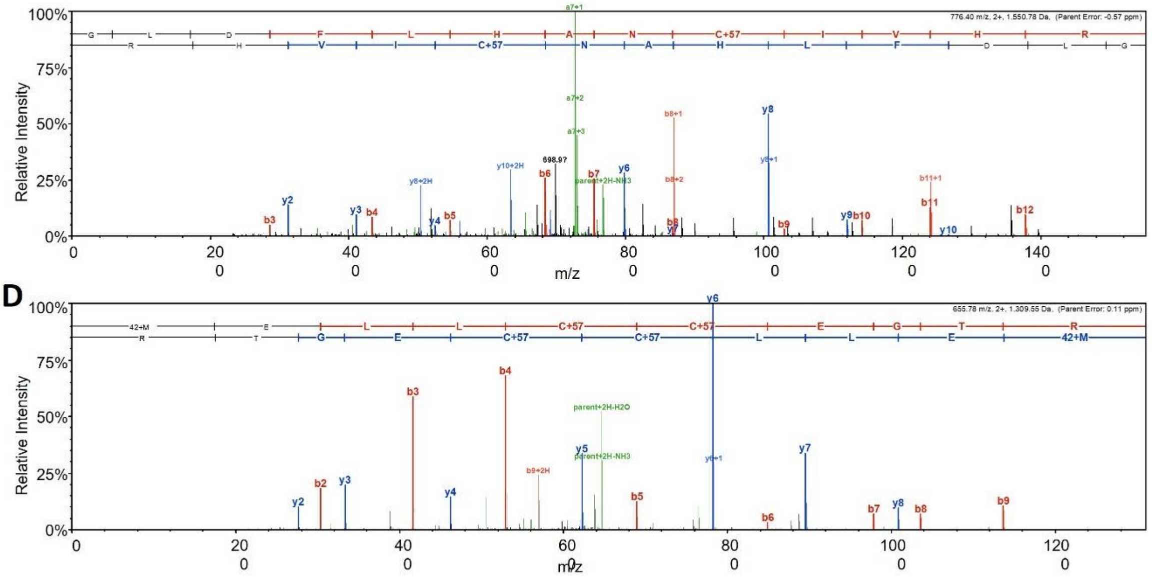

Figure S6. Identification of CDK4 and cyclin D3 at ~75kDa by LC-MS/MS

Protein identification was performed by LC-MS/MS of a protein sample excised at ~75kDa. Samples were produced from HeLa cells overexpressing cyclin D3 and CDK4, treated for 15 minutes with or without 200 µM H_2O_2, purified by co-immunoprecipitation and separated by SDS-PAGE under non-reducing or reducing conditions, respectively. In-gel reduction, alkylation and enzymatic digestion was performed before analysis by LC-MS/MS and database searching against the Human Taxonomy. **A)** The number of spectra assigned to CDK4 (P11802) and cyclin D3 (P30281) shows enrichment of CDK4 and cyclin D3 at ~75kDa under oxidising conditions. Mean +/- SD from 4 independent experiments. **B)** 92% sequence coverage of CDK4 and 68% sequence coverage of CCND3 identified by LC-MS/MS. The peptides of interest are shown in red boxes, with all detected residues shown in yellow and all modified residues highlighted in green. **C)** A peptide of *m/z* 776.20^{2+} was detected by LC-MS/MS analysis, which corresponds to the CDK4 peptide ^{127}GLDFLHANC$_{carb}$IVHR^{139} showing carbamidomethylation of C135. **D)** A peptide of *m/z* 655.78^{2+} was detected by LC-MS/MS analysis, which corresponds to the CCND3 peptide ^{1AC}MELLC$_{carb}$C$_{carb}$EGTR^{10} showing carbamidomethylation of C5 and C6.

A

	Total Spectrum Count		
	H_2O_2-treated	Reduced	P-value
CDK4	277.0 +/- 28.3	10.5 +/- 0.7	0.0056
CCND3	82.5 +/- 7.8	2.0 +/- 0	0.0046

B

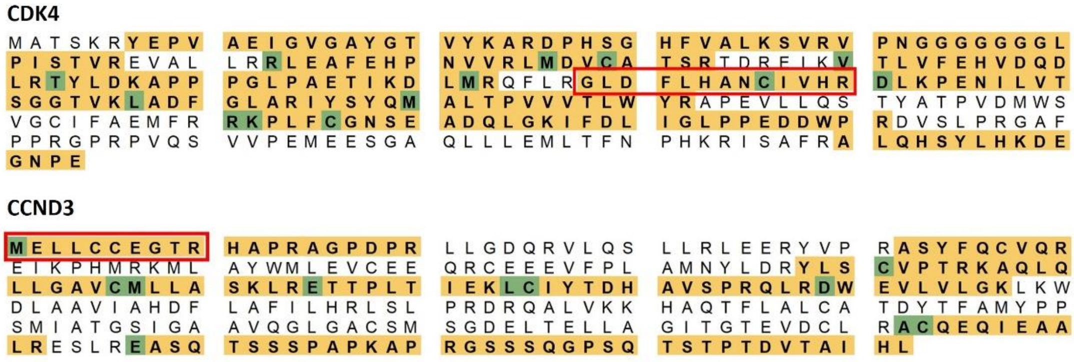

C

GLDFLHAN**C**IVHR (M_r 1494.7364 Da)

|

MELL**CC**EGTR (M_r 1154.5003 Da)

'MELLCCEGTRGLDFLHANCIVHR' (M_r 2649.2367 Da)

Figure S7. Identification of disulfide cyclin D-CDK4 by LC-MS/MS

Identification of disulfide cyclin D-CDK4 was performed by LC-MS/MS of a protein sample excised at ~75kDa. Samples were produced from HeLa cells overexpressing cyclin D3 and CDK4, treated for 15 minutes with or without 200 µM H_2O_2, purified by co-immunoprecipitation and separated by SDS-PAGE under non-reducing or reducing conditions, respectively. Gel bands were processed by enzymatic digestion without reduction and alkylation before analysis by LC-MS/MS and database searching against a bespoke database. **A)** The number of spectra assigned to CDK4 (P11802) and cyclin D3 (P30281) shows enrichment of CDK4 and cyclin D3 at ~75kDa under oxidising conditions. Mean +/- SD from 2 independent experiments. **B)** 68% sequence coverage of CDK4 and 47% sequence coverage of CCND3 identified by LC-MS/MS. The peptides of interest are shown in red boxes, with all modified residues highlighted in green. **C)** The peptides containing CDK4 C135 (^{127}GLDFLHANCIVHR^{139}) and cyclin D3 C5/6 (^{1}MELLCCEGTR^{10}) were concatenated with the methionine at the first point of the sequence to allow for acetylation and methionine+acetylation predictions. The predicted molecular weights are reported in red.

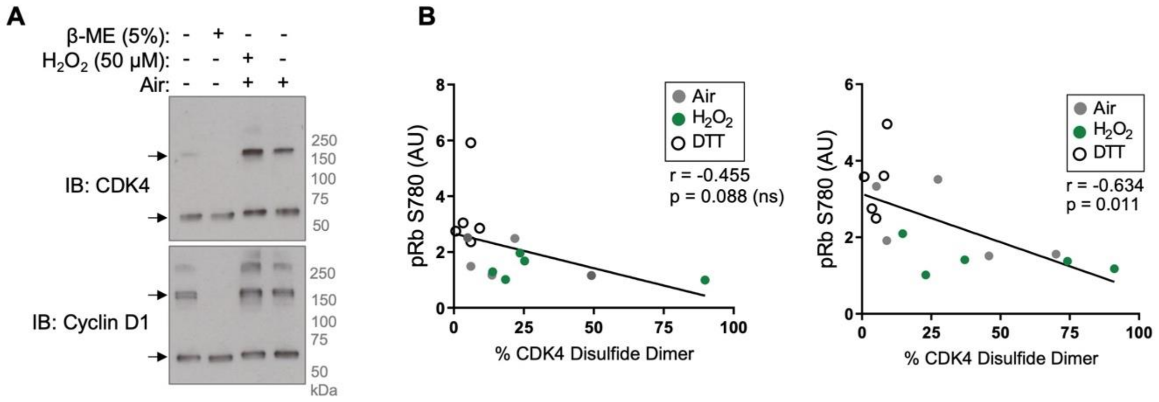

Figure S8. Cyclin D1-CDK4 recombinant protein assays

A) Recombinant cyclin D-CDK4 forms an oxidant-induced disulfide bond. Recombinant cyclin D1-CDK4 protein complex (Invitrogen, PV4400) was either untreated, reduced with β-ME, or oxidized in air with or without 50 µM H_2O_2 for 15 minutes. Monomeric and disulfide dimeric cyclin D1 and CDK4 (indicated by black arrows) were detected by non-reducing immunoblotting (n=2). **B)** An *in vitro* kinase activity assay shows a negative correlation between pRb S780 and disulfide CDK4. Recombinant cyclin D1-CDK4 protein (Invitrogen, #PV4400) was incubated with 2 mM DTT, 50 µM H_2O_2 or vehicle (H_2O) for 30 minutes at 30°C to reduce or oxidize, respectively. After the addition of recombinant Rb protein (Abcam, #ab83205), the assay was incubated for 10, 30 or 60 minutes at 30°C. pRb S780 phosphorylation and disulfide CDK4 were detected by immunoblotting. Graphs show CDK4 oxidation and Rb phosphorylation after incubation of the assay for 10 minutes (left) or 30 minutes (right). Analysis was performed using Simple Linear Regression model showing the line of best fit and Pearson's correlation coefficient (r) (n=5 independent experiments).

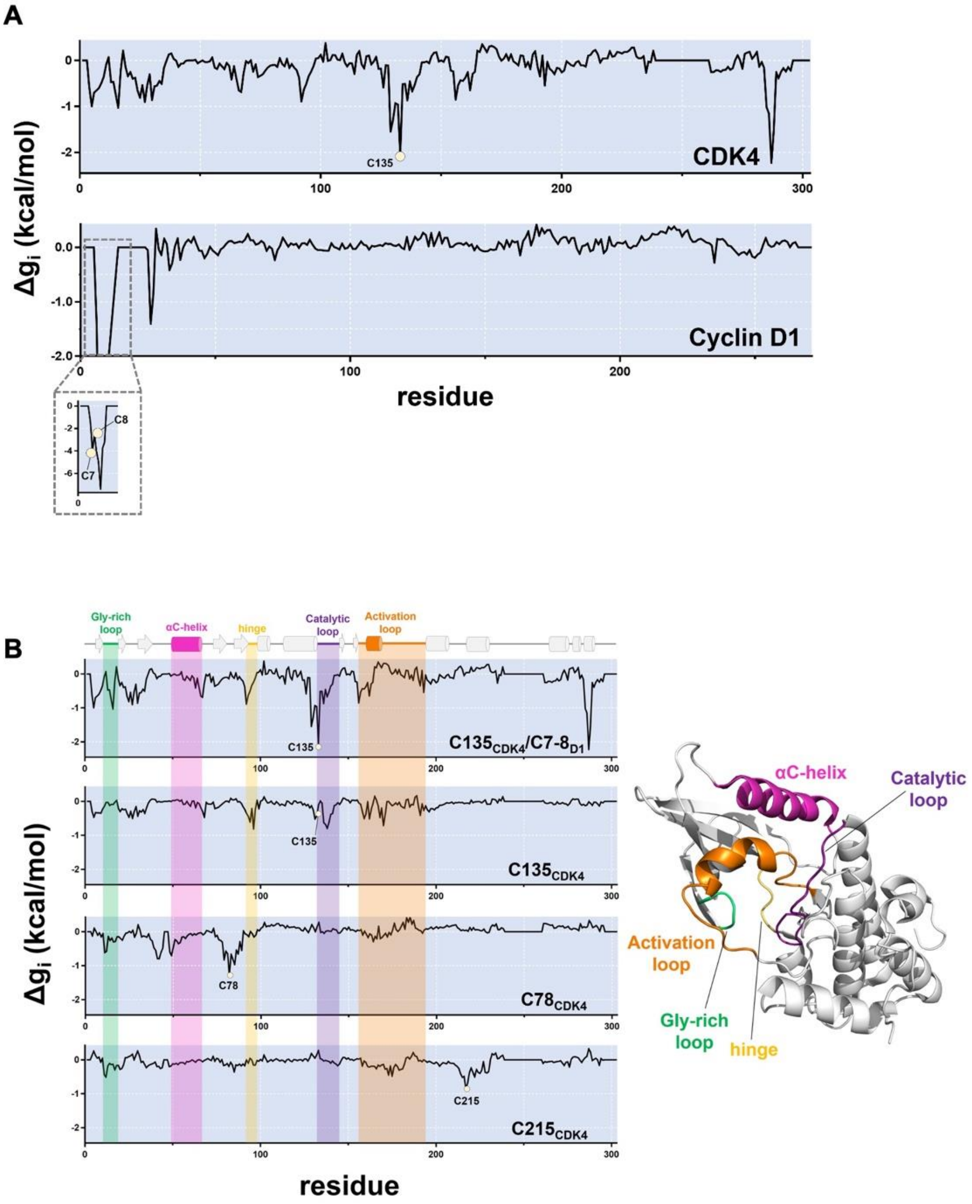

Figure S9. Per-residue allosteric free energy (Δg_i) plots obtained from AlloSigMA calculations

Positive and negative Δg_i values correspond to increased and decreased free energies associated with system perturbation. The probed amino acids are highlighted with yellow circles. **A)** Probing of CDK4 C135 and cyclin D1 C7/8 residues in the cyclin D1-CDK4 complex. The plots report the allosteric landscape for both CDK4 and cyclin D1. **B)** Comparison of CDK4 allosteric landscapes obtained by probing CDK4 C135 and cyclin D1 C7/8 in the cyclin D1-CDK4 complex, with the probing of CDK4 C78, CDK4 C135 or CDK4 C215 in isolation. The CDK4 secondary structure elements are reported at the top. The colored boxes highlight the Δg_i values of structural elements of interest, which are mapped on the structure of CDK4 in the inset on the right side.

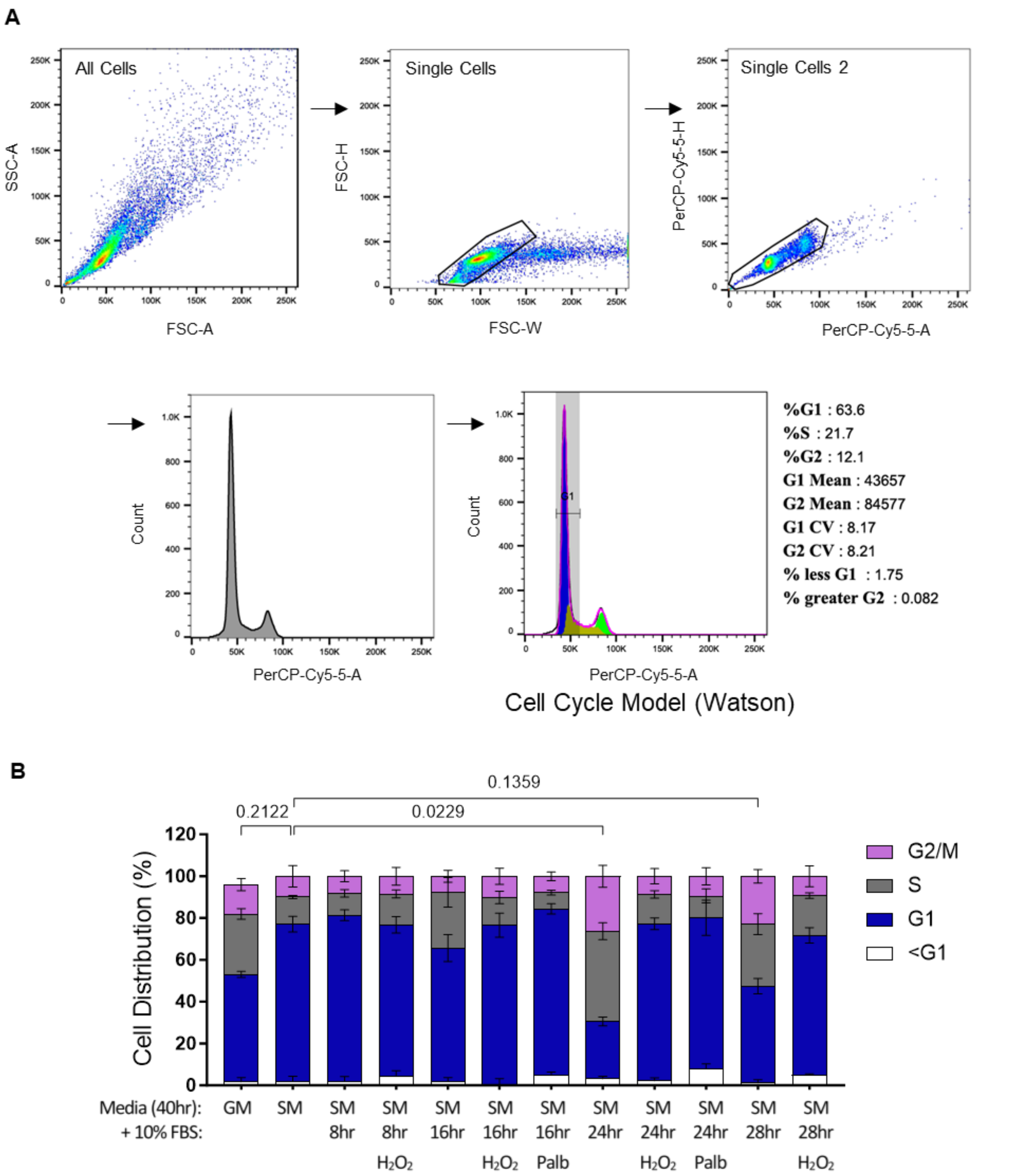

Figure S10. Cell cycle analysis of propidium iodide stained HPASMCs

A) Gating strategy of cell cycle analysis of propidium iodide-stained HPASMCs gated to select single cells only. These cells were then modelled using a Watson Pragmatic cell cycle model using FlowJo™ to categorize cells into each cell cycle phase. **B)** Graph showing the proportion of cells in each cell cycle phase after maintenance in growth media (GM) or starvation media (SM) for 40 hours, after which cells were stimulated with 10% FBS for 8, 16, 24 or 28 hours in the absence or presence of 50 µM H_2O_2 or 10 µM palbociclib. As the data sample size with an n<6 cannot be reliably tested for normality, *P* values are calculated using a non-parametric Kruskal-Wallis test followed by Dunn's multiple comparisons to compare between the number of G0/1 phase cells (blue) at each timepoint with the proportion of synchronized cells in the G0/1 phase after 40 hours in starvation media (SM, bar 2, blue) (GM, SM, 8 and 24 hours, n=5 experimental samples per group; 16 and 28 hours, n=4 experimental samples per group).

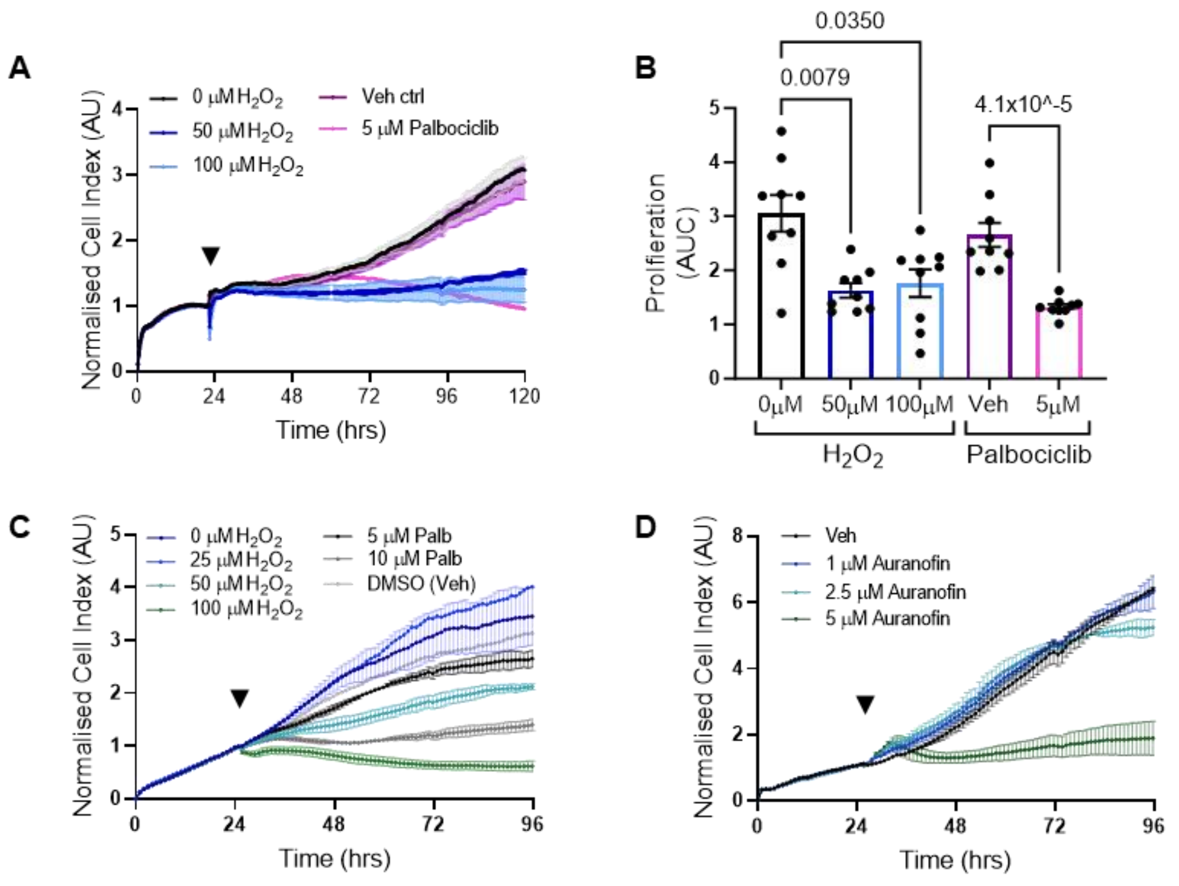

Figure S11. Oxidation inhibits proliferation of HPAECs and MCF7 cells.

A and B) HPAECs were seeded in xCelligence RTCA E-plates at 2.5×10^3 cells/well in Endothelial Cell Growth Medium. After 24 hours, cells were treated with H_2O_2 or Palbociclib. **A)** Proliferation was measured by electrical impedance (cell index) for a subsequent 96 hours and normalized to treatment time. **B)** Proliferation rate of HPAECs was quantified using area under the curve from the time of treatment. As the data sample size with an n<6 cannot be reliably tested for normality, *P* values are calculated using a non-parametric Kruskal-Wallis test followed by Dunn's multiple comparisons to compare between H_2O_2-treatment conditions and 0μM control or unpaired two-tailed non-parametric Mann-Whitney test to compare between vehicle and Palbociclib treatment (n=3 independent experiments, each performed in triplicate); the results are shown as means±SEM. **C and D)** MCF7 cells were seeded in xCELLigence RTCA E-plates at 3×10^4 cells/well. After 24 hours, cells were treated with **C)** H_2O_2 or Palbociclib (n=3 independent experiments, each performed in duplicate), or **D)** auranofin (n=4 independent experiments, each performed in duplicate). Proliferation was measured by electrical impedance (cell index) for a subsequent 72 hours and normalized to treatment time (indicated by black arrowhead).

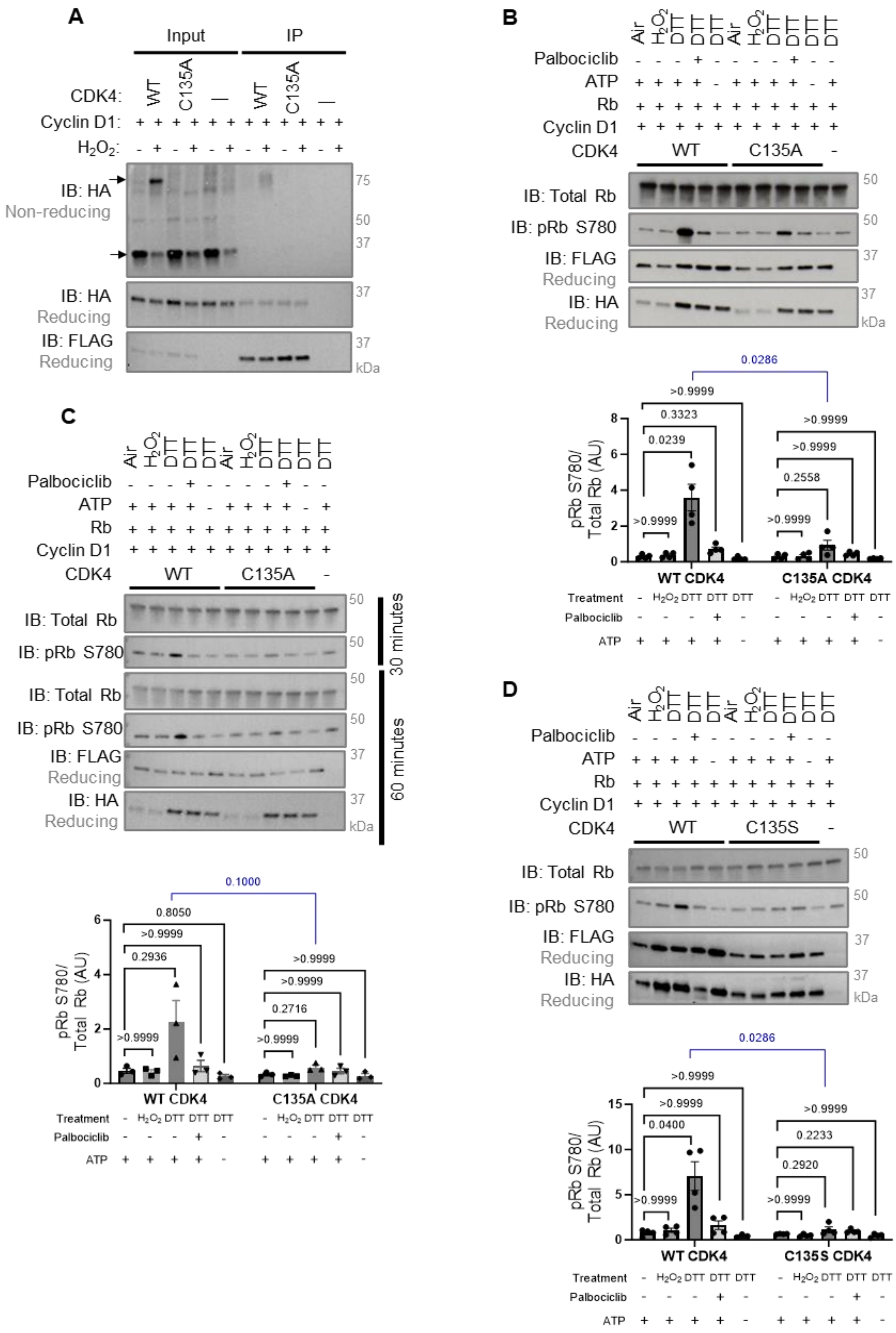

Figure S12. Cyclin D1-CDK4 co-immunoprecipitation and kinase activity assays

A) Both WT or C135A CDK4 co-immunoprecipitate cyclin D1. Disulfide cyclin D1 is only observed with WT CDK4 but not C135A CDK4. WT cyclin D1-HA and WT or C135A CDK4-FLAG were overexpressed in HPASMCs for 24 hours before treatment with H_2O_2 for 15 minutes. Cyclin D1 and CDK4 were co-immunoprecipitated using FLAG-agarose beads under non-reducing conditions. Cyclin D1 and CDK4

were detected by immunoblotting under non-reducing or reducing conditions using antibodies against HA or FLAG, respectively. **B)** An *in vitro* kinase activity assay of WT or C135A cyclin D1-CDK4 co-immunoprecipitated from HPASMCs. **C)** An *in vitro* kinase activity assay of WT or C135A cyclin D1-CDK4 co-immunoprecipitated from HAP1 CDK4 KO cells. **D)** An *in vitro* kinase activity assay of WT or C135S cyclin D1-CDK4 co-immunoprecipitated from HAP1 CDK4 KO cells. **B-D)** The co-immunoprecipitated protein was incubated with 2 mM DTT, 1 µM H_2O_2 or vehicle (H_2O) for 30 minutes to reduce or oxidize, respectively. Rb-GST tagged protein substrate was added in the absence or presence of 1 µM palbociclib and incubated for a further 30 or 60 minutes at 30°C. Reducing immunoblots were probed for pRb S780, total Rb, CDK4-FLAG, and cyclin D1-HA. Graphs show Rb phosphorylation at S780, normalized to total Rb. As the data sample size with an $n<6$ cannot be reliably tested for normality, *P* values are calculated using a non-parametric Kruskal-Wallis test followed by Dunn's multiple comparisons to compare all group treatment conditions to Air + ATP condition within WT or C135A/S mutant group; non-parametric unpaired 2-tailed Mann-Whitney test was used to compare DTT-treated conditions between WT and C135A/S mutants (**B:** WT or C135A mutants co-immunoprecipitated from HPASMCs, n=4; **C:** WT or C135A mutants co-immunoprecipitated from HAP1 cells, n=3; **D:** WT or C135S mutants co-immunoprecipitated from HPASMCs, n=4 independent experiments). The results are shown as means±SEM. Please note that for n=3, the only minimal achievable *P* value with the non-parametric unpaired 2-tailed Mann-Whitney test used is 0.1000.

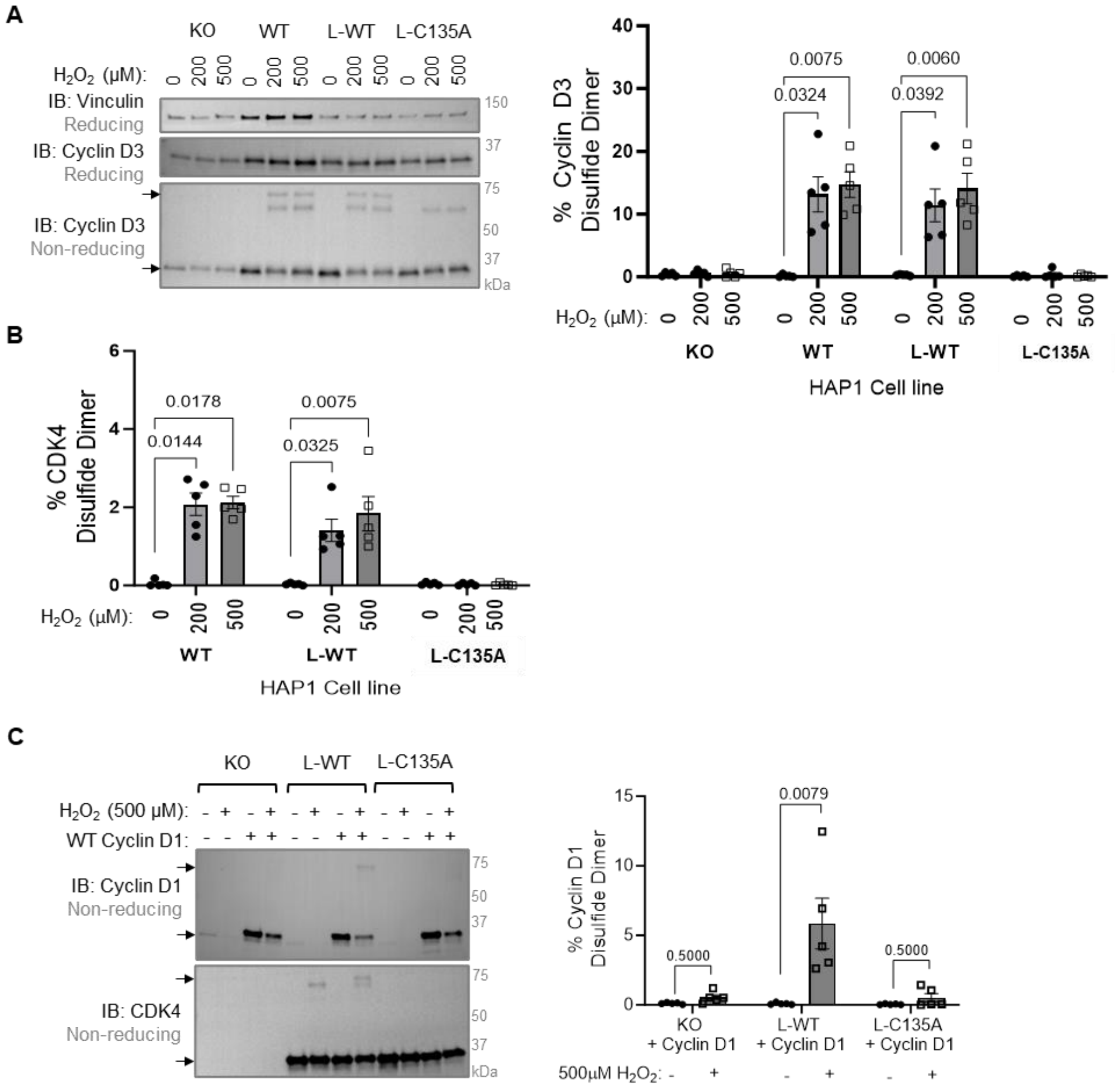

Figure S13. L-C135A HAP1 cells are 'redox-dead'

A) The cyclin D3-CDK4 disulfide dimer is absent in CDK4 KO HAP1 cells and L-C135A mutant HAP1 cells. WT HAP1 cells, CDK4 KO HAP1 cells, L-WT or L-C135A CDK4 HAP1 stable cell lines were treated with H_2O_2 for 15 minutes. Monomeric and disulfide dimeric cyclin D3 (indicated by black arrows) were detected by immunoblotting under non-reducing conditions. Cyclin D3 and vinculin (loading control) expression was detected by immunoblotting under reducing conditions. As the data sample size with an n<6 cannot be reliably tested for normality, *P* values are calculated using a non-parametric Kruskal-Wallis test followed by Dunn's multiple comparisons to compare H_2O_2-induced disulfide formation with 0 µM H_2O_2 treatment within each cell type (n=5 independent experiments). **B)** The cyclin D-CDK4 disulfide dimer is absent in L-C135A CDK4 HAP1 cells. HAP1 cells were treated with H_2O_2 for 15 minutes. Quantification shows the CDK4 disulfide dimer, as detected by non-reducing immunoblotting. *P* values are calculated using a non-parametric Kruskal-Wallis test followed by Dunn's multiple comparisons to compare H_2O_2-induced disulfide formation with 0 µM H_2O_2 treatment within each cell type (n=5 independent experiments). **C)** The cyclin D1-CDK4 disulfide dimer is absent in CDK4 KO HAP1 cells and L-C135A mutant HAP1 cells. CDK4 KO HAP1 cells, L-WT or L-C135A HAP1 stable cell lines were overexpressed with WT cyclin D1-HA for 24 hours and treated with 500 µM H_2O_2 for 15 minutes. Non-reducing immunoblots were probed for monomeric and disulfide dimeric cyclin D1 and CDK4 (indicated by black arrows). *P* values are calculated using a non-parametric unpaired 2-tailed Mann-Whitney test to compare between 0 µM and 500 µM H_2O_2-treatment within each cell type (n=5 independent experiments).

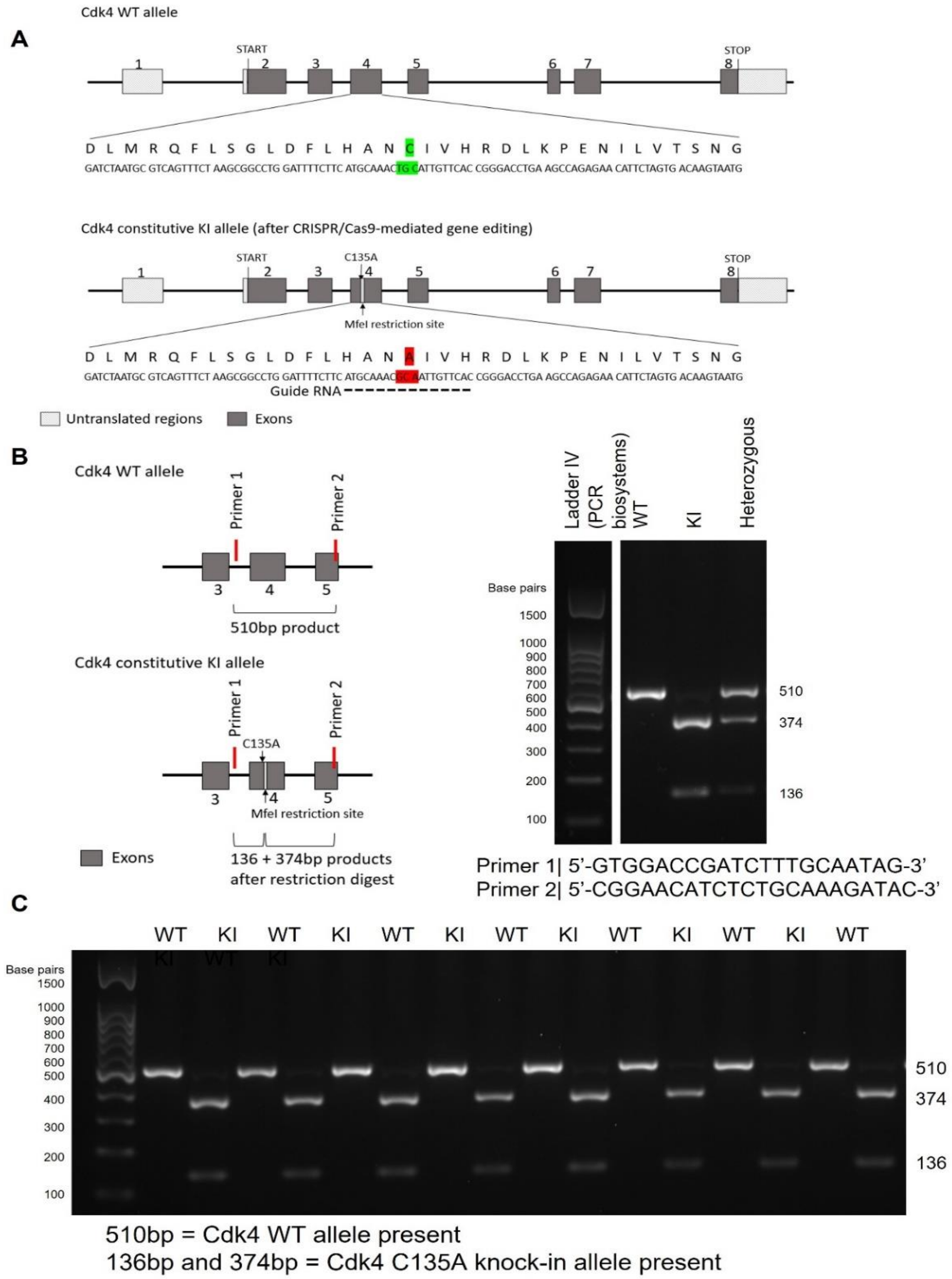

Figure S14. Generation and genotyping of C135A CDK4 knock-in mice.

A) Schematic diagram of the Cdk4 gene locus in wildtype (WT) mice and in knock-in (KI) mice constitutively expressing the Cdk4 C135A point mutation. KI mice were generated using CRISPR/Cas9-mediated genetic engineering. Exon 4 of the Cdk4 gene was targeted in embryos using a specific guide RNA and oligonucleotide for homology-directed repair to mutate the TGC codon encoding cysteine in position 135 to GCA, encoding alanine. **B)** Schematic diagram and example agarose gel image showing the products of PCR amplification and MfeI restriction digest. The Cdk4 C135A point mutation introduces a MfeI restriction site. Following MfeI digestion, a 510bp product indicates the presence of

the Cdk4 WT allele. A 136bp and 374bp product indicates the presence of the Cdk4 KI allele. **C**) Representative example agarose gel image showing the products of PCR amplification and MfeI restriction digest for some WT and KI mice constitutively expressing the Cdk4 C135A point mutation. A 510 bp product indicated the presence of Cdk4 WT allele, while both 136bp and 374bp product indicates the presence of the Cdk4 KI allele.

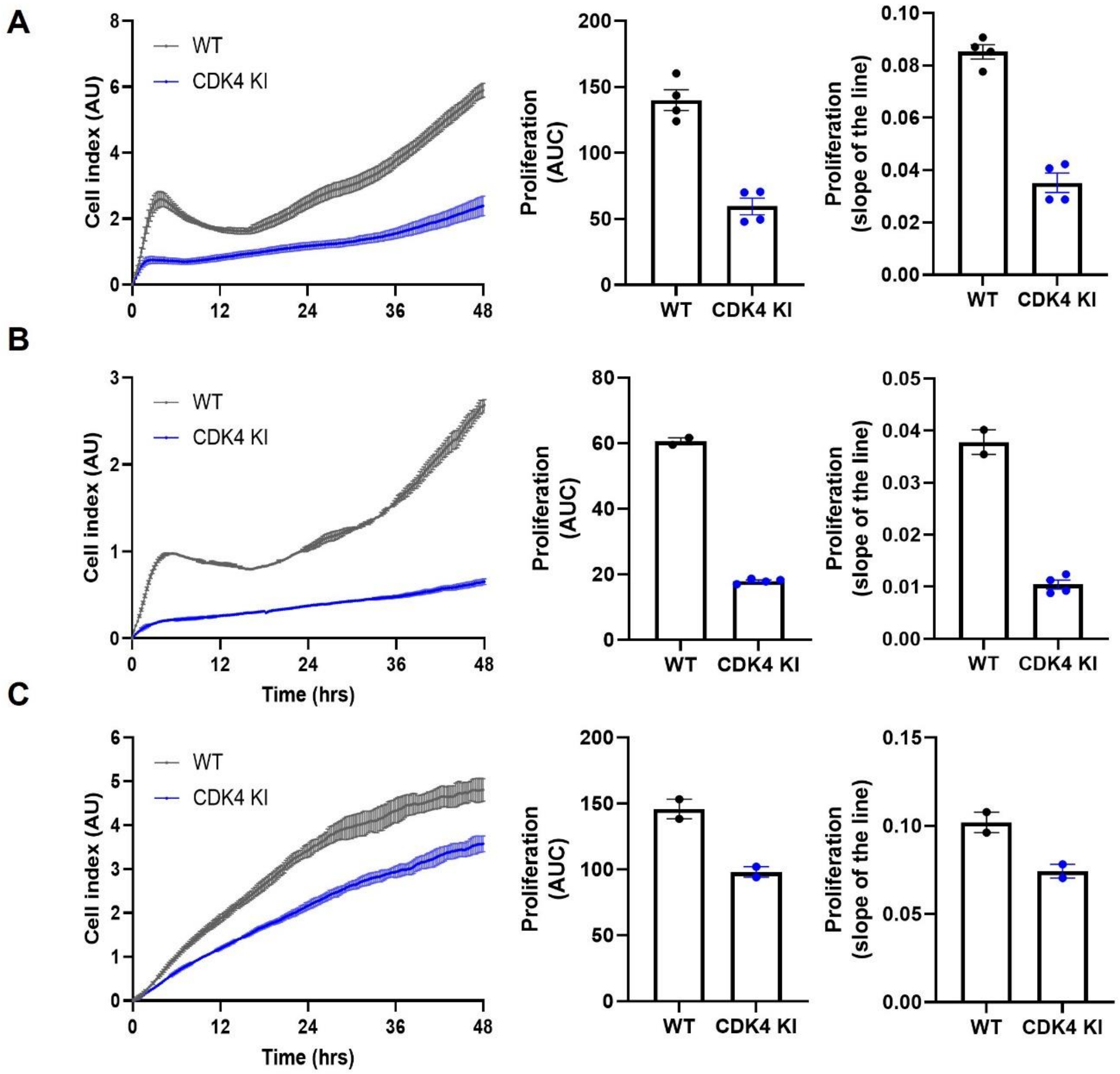

Figure S15. Pulmonary vascular C135A CDK4 KI cells have impaired proliferation.
A, B) Mouse pulmonary arterial smooth muscle cells (PASMCs) isolated from lungs of C135A CDK4 KI mice demonstrate a decreased proliferation rate compared to cells isolated from a WT littermate. Proliferation of PASMCs was measured by electrical impedance (cell index) for 48 hours. Cells were seeded in xCELLigence RTCA E-plates at (**A**) $8x10^3$ cells/well or (**B**) $3x10^3$ cells/well. Proliferation rate was analyzed by area under the curve of PASMCs, and the slope is calculated from the linear portion of the graph. One WT or two separate CDK4 KI mouse lungs were used for cell isolation, and the proliferation experiments were run in quadruplicate (A) or duplicate (B). **C)** Mouse lung endothelial cells (MLECs) isolated from C135A CDK4 KI mice demonstrate decreased proliferation rate compared to MLECs isolated from WT mice. Proliferation of MLECs was measured by electrical impedance (cell index) for 48 hours. Cells were seeded in xCELLigence RTCA gelatin and fibronectin-coated E-plates at $2.5x10^3$ cells/well. Proliferation rate was analyzed by area under the curve of MLECs, and the slope is calculated from the linear portion of the graph (first 14 hours). Two WT or CDK4 KI mouse lungs were pooled for one cell isolation, and the proliferation experiment was run in duplicate.

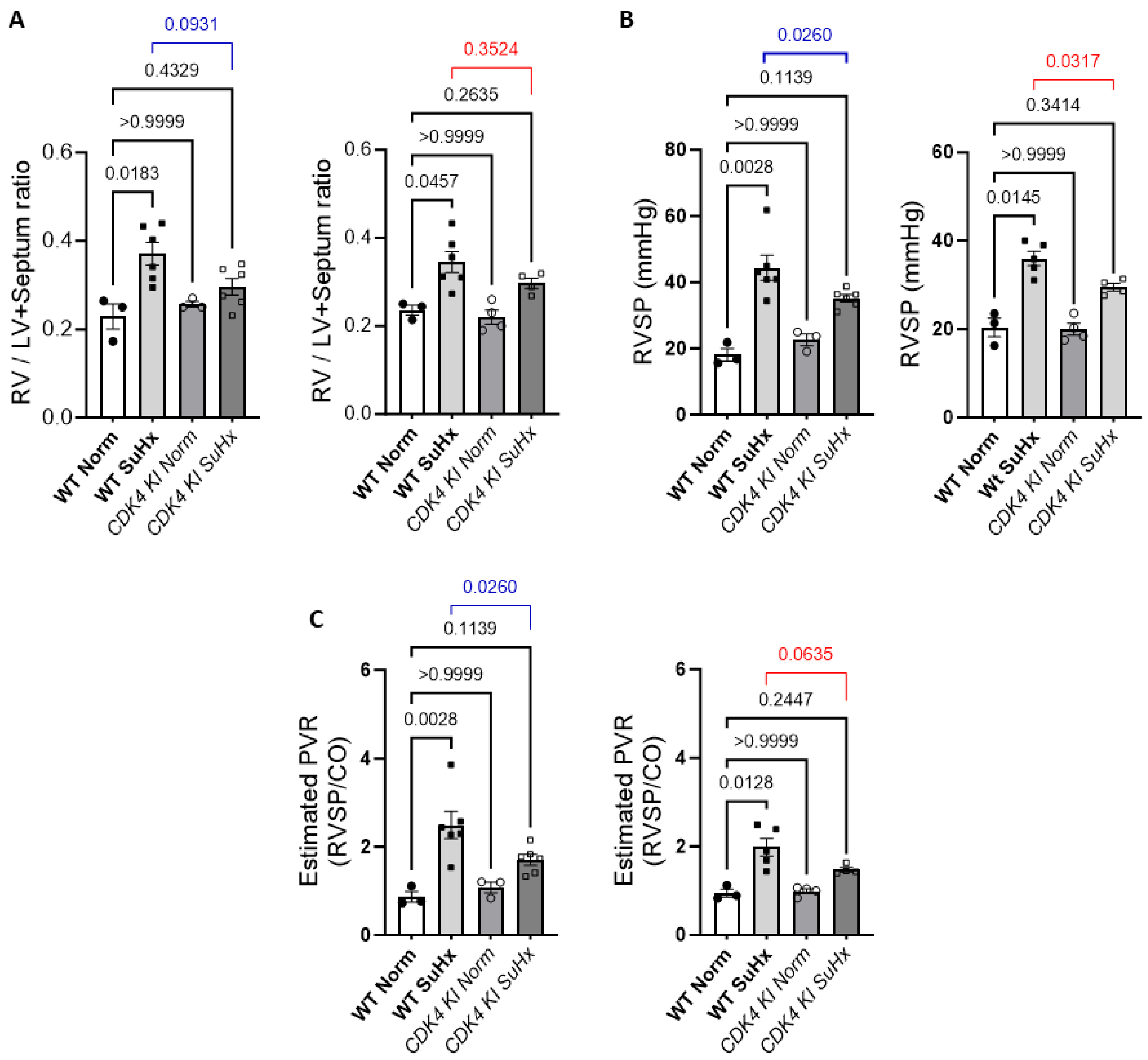

Figure S16. 'Redox-dead' C135A CDK4 KI mice develop a less severe disease phenotype in the Sugen/hypoxia experimental PH model.

A) RV hypertrophy (RV/LV+Septum ratio), **B)** RVSP (mmHg) and **C)** estimated pulmonary vascular resistance measured in males (left, blue *P* value between WT SuHx and CDK4 KI SuHx) or female (right, left, red *P* value between WT SuHx and CDK4 KI SuHx) sexes of WT or CDK4 KI mice to assess the severity of PH. As the data sample size with low n-number (n<6) cannot be reliably tested for normality, *P* values are calculated using a non-parametric Kruskal-Wallis test followed by Dunn's multiple comparisons to compare all groups with WT Norm; a non-parametric unpaired 2-tailed Mann-Whitney test was used to compare between WT SuHx and CDK4 KI SuHx (**Males:** WT Norm n=3, WT SuHx n=6, CDK4 KI Norm n=3, CDK4 KI SuHx n=6; **Females:** WT Norm n=3, WT SuHx n=6, CDK4 KI Norm n=4, CDK4 KI SuHx n=4), and the results are shown as means±SEM.

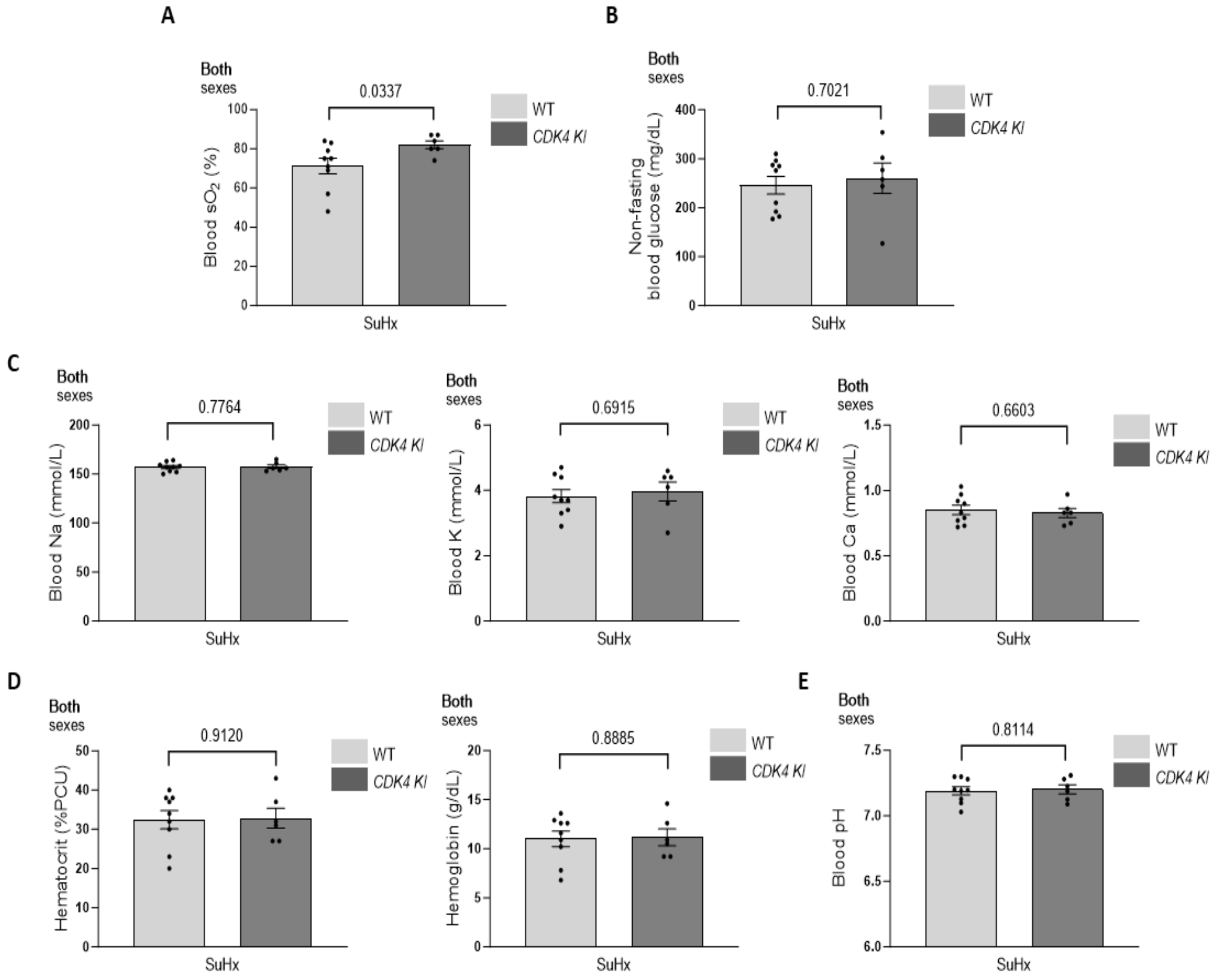

Figure S17. Hematological parameters were similar in C135A CDK4 KI or WT mice subjected to Sugen/hypoxia experimental PH model.

Comparison of blood sodium (Na), potassium (K), calcium (Ca) **(A)**, hematocrit and hemoglobin concentration **(B)**, blood pH **(C)**, oxygen saturation (SO_2) **(D)** and non-fasting blood glucose (**E**) examined using a blood gas analyser in WT or KI male and female mice subjected to Sugen/hypoxia experimental models of PH. Statistical analysis was performed using a one-way ANOVA followed by Tukey's multiple comparisons test to compare between all groups. An assessment of the data normality was performed by the Shapiro-Wilk test; all data parameters presented in the figure passed the normality test. As the data passed the test for normality, *P* values were calculated using the parametric unpaired and 2-tailed t-test with Welch's correction to compare between two groups (WT SuHx n=9, KI SuHx n=6), and the results are shown as means±SEM.

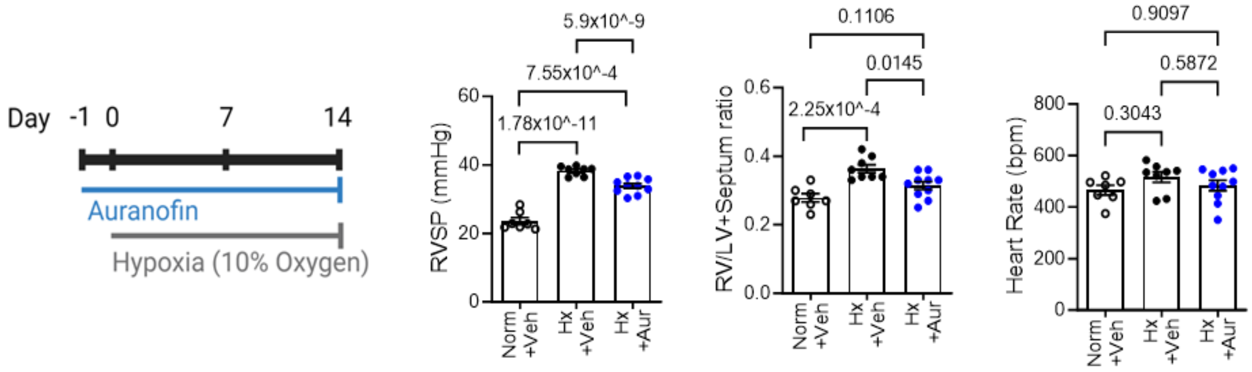

Figure S18. Auranofin attenuates disease severity in the hypoxia-induced mouse model of PH. The hypoxia-induced mouse model of PH was induced by exposure to hypoxia for two weeks (Hx, 10% O_2). Control mice were maintained in normoxia (Norm, 21% O_2). Mice were continuously treated with auranofin (Aur, 8.5 mg/kg/day) or vehicle (Veh, 60% DMSO/saline) via osmotic minipump for two weeks. RVSP (mmHg), RV hypertrophy (RV/LV+septum ratio), and heart rate (beats per minute) were measured to assess the severity of PH. An assessment of the data normality was performed by the Shapiro-Wilk test; all data parameters presented in the figure passed the normality test. As the data passed the test for normality, *P* values were calculated using the parametric test one-way ANOVA followed by Tukey's multiple comparisons to compare between all groups (Norm+Veh n=7, Hx +Veh n=8, Hx+Aur n=10), and the results are shown as means±SEM.

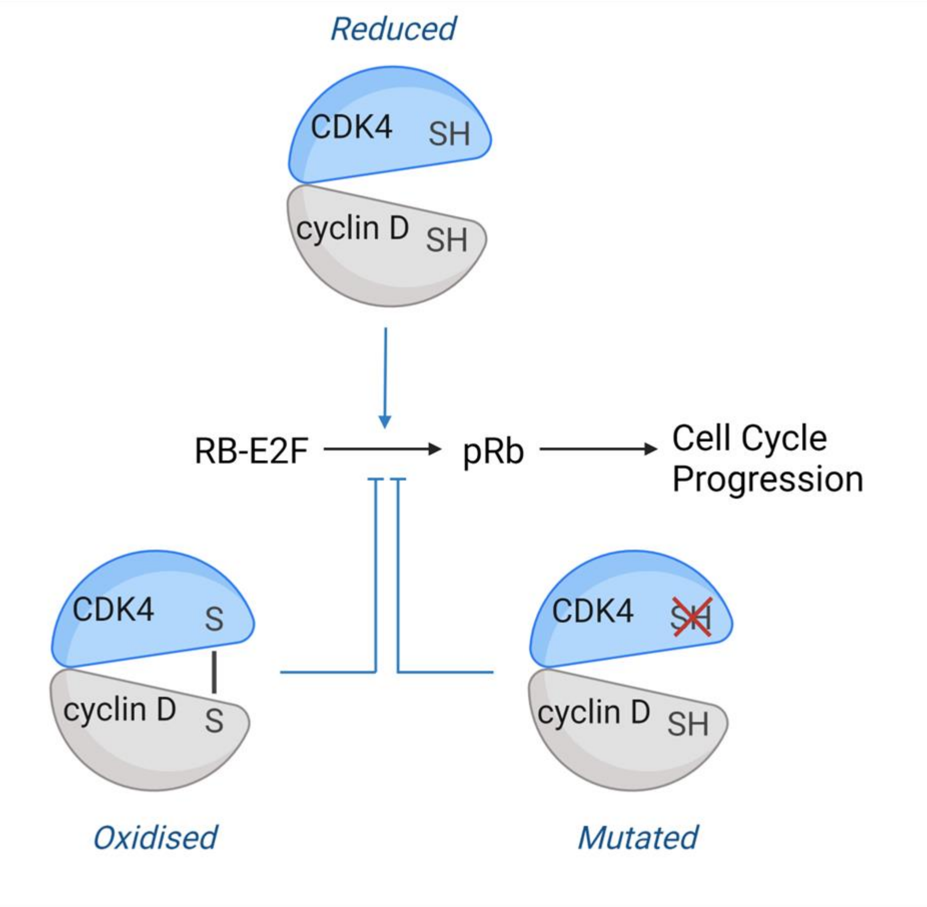

Figure S19. A scheme of cyclin D-CDK4 redox regulation at the critical cysteine residue C135
Under reducing conditions, cyclin D-CDK4 phosphorylates Rb, stimulating progression through the cell cycle. Upon oxidation, cyclin D-CDK4 forms an intermolecular heterodimeric disulfide bond between cyclin D1 C7/8 and CDK4 C135, which inhibits kinase activity. Similarly, mutation of CDK4 C135 mimics the disulfide bond by impairing the critical cysteine residue, resulting in decreased Rb phosphorylation. Structural analysis suggests this effect is due to rigidification of CDK4, which impairs structural rearrangement into the active conformation.
