# Supplementary material for: Cyclin D-CDK4 Disulfide Bond Attenuates Pulmonary Vascular Cell Proliferation
Source: Circ Res. 2023 Nov 13;133(12):966–88. doi: 10.1161/CIRCRESAHA.122.321836 (PMC10699508; doi:10.1161/CIRCRESAHA.122.321836)
Supplement: Supplementary file 3 [file res-133-0966-s003.pdf]

Major Resources Table

In order to allow validation and replication of experiments, all essential research materials listed in the Methods should be included in the Major Resources Table below. Authors are encouraged to use public repositories for protocols, data, code, and other materials and provide persistent identifiers and/or links to repositories when available. Authors may add or delete rows as needed.

Animals (in vivo studies)

Species	Vendor or Source	Background Strain	Sex	Persistent ID / URL
C57BL/6J mice	Charles River Laboratories	C57BL/6J Mice (JAX® Mice Strain)	M	https://www.criver.com/products-services/find-model/c57bl6j-mice-jax-strain?region=3671
Wistar Kyoto (WKY) Rat	Charles River Laboratories	WKY/NCrl	M	https://www.criver.com/products-services/find-model/wistar-kyoto-wky-rat?region=3671

Genetically Modified Animals

	Species	Vendor or Source	Background Strain	Other Information	Persistent ID / URL
**Parent - Male**	C57BL/6NTac-*Cdk4em8392(C135A)Tac*	Taconic Biosciences for Dr Olena Rudyk Lab	C57BL/6N	Novel constitutive C135A CDK4 knock-in mice were custom-generated by CRISPR/Cas9 mediated gene editing by Taconic Biosciences for Dr Olena Rudyk Lab	https://www.taconic.com/genetically-engineered-animal-models/knock-in-mice/#constitutive (generic website)
**Parent - Female**	C57BL/6NTac-*Cdk4em8392(C135A)Tac*	Taconic Biosciences for Dr Olena Rudyk Lab	C57BL/6N	Novel constitutive C135A CDK4 knock-in mice were custom-generated by CRISPR/Cas9 mediated gene editing by Taconic Biosciences for Dr Olena Rudyk Lab	https://www.taconic.com/genetically-engineered-animal-models/knock-in-mice/#constitutive (generic website)

Antibodies

Target antigen	Vendor or Source	Catalog #	Working concentration	Lot # (preferred but not required)	Persistent ID / URL
CDK4	Cell Signaling Technologies	12790	1:1000		https://www.cellsignal.com/products/primary-antibodies/cdk4-d9g3e-rabbit-mab/12790

DOI [to be added]

CDK4 (H-22)	Santa Cruz Biotechnology	sc-601	1:1000		https://www.scbt.com/p/cdk4-antibody-h-22
Cyclin D1	Cell Signaling Technologies	2922	1:1000		https://www.cellsignal.com/products/primary-antibodies/cyclin-d1-antibody/2922
Cyclin D3	Cell Signaling Technologies	2936	1:1000		https://www.cellsignal.com/products/primary-antibodies/cyclin-d3-dcs22-mouse-mab/2936
Rb Total	Cell Signaling Technologies	9309	1:1000		https://www.cellsignal.com/products/primary-antibodies/rb-4h1-mouse-mab/9309
pRb Ser780	Cell Signaling Technologies	8180	1:1000		https://www.cellsignal.com/products/primary-antibodies/phospho-rb-ser780-d59b7-rabbit-mab/8180
pRb Ser795	Cell Signaling Technologies	9301	1:1000		https://www.cellsignal.com/products/primary-antibodies/phospho-rb-ser795-antibody/9301
pRb Ser807/811	Cell Signaling Technologies	8516	1:1000		https://www.cellsignal.com/products/primary-antibodies/phospho-rb-ser807-811-d20b12-xp-rabbit-mab/8516
Vinculin	Cell Signaling Technologies	13901	1:1000		https://www.cellsignal.com/products/primary-antibodies/vinculin-e1e9v-xp-rabbit-mab/13901
GAPDH	Cell Signaling Technologies	2118	1:1000		https://www.cellsignal.com/products/primary-antibodies/gapdh-14c10-rabbit-mab/2118
FLAG-tag	Cell Signaling Technologies	2368	1:1000		https://www.cellsignal.com/products/primary-antibodies/dykddddk-tag-antibody-binds-to-same-epitope-as-sigma-aldrich-anti-flag-m2-antibody/2368
HA-tag	Cell Signaling Technologies	3724	1:1000		https://www.cellsignal.com/products/primary-antibodies/ha-tag-c29f4-rabbit-mab/3724
Anti-Mouse HRP-linked Secondary	Cell Signaling Technologies	7076	1:1000		https://www.cellsignal.com/products/secondary-antibodies/anti-mouse-igg-hrp-linked-antibody/7076

DOI [to be added]

Anti-Rabbit HRP-linked Secondary	Cell Signaling Technologies	7074	1:1000		https://www.cellsignal.com/products/secondary-antibodies/anti-rabbit-igg-hrp-linked-antibody/7074
α-SMA	DAKO (Agilent Technologies)	M0851	1:100		https://www.agilent.com/store/productDetail.jsp?catalogId=M085101-2
Negative control mouse IgG2a	DAKO (Agilent)	X0943	1:100		https://www.agilent.com/store/en_US/Prod-X094301-2/X094301-2
N-Histofine MOUSESTAIN kit	Nichirei Bio-sciences	414322F	N/A (as per instruction)		https://www.2bscientific.com/Products/Nichirei-Bioscience/414322F/N-Histofine-MOUSESTAIN-KIT<br><br>https://nichireibiosciences.com/wp-content/themes/nichirei/pdf/414321f%20MOUSESTAIN%20KIT.pdf

DNA/cDNA Clones

Clone Name	Sequence	Source / Repository	Persistent ID / URL
CDK4-FLAG (OHu21245)	pcDNA3.1-C-(k)DYK	GenScript Gene Synthesis	https://www.genscript.com/gene/homo-sapiens/1019/cdk4.html?page_no=1&position_no=1&sensors=search_information#nm_000075.4
Cyclin D1-HA	pcDNA3	Addgene (#11181)	https://www.addgene.org/11181/
Cyclin D3-HA (OHu21276C)	pcDNA3.1	GenScript Gene Synthesis	https://www.genscript.com/gene/homo-sapiens/896/ccnd3.html?page_no=1&position_no=1&sensors=googlesearch#nm_001760.5
CDK4-FLAG (OHu21245)	pLV-Puro-CMV-hCDK4	GenScript Gene Synthesis	https://www.genscript.com/gene/homo-sapiens/1019/cdk4.html?page_no=1&position_no=1&sensors=search_information#nm_000075.4
pLP1 packaging plasmid	pLP1-CMV 8889bp	Invitrogen (Life Technologies)	# K4950-00 https://tools.thermofisher.com/content/sfs/manuals/virapower_lentiviral_system_man.pdf
pLP2 packaging plasmid	pLP2-RSV 4180bp	Invitrogen (Life Technologies)	# K4950-00 https://tools.thermofisher.com/content/sfs/manuals/virapower_lentiviral_system_man.pdf

DOI [to be added]

pLP/VSVG envelope plasmid	pLP1-CMV 5821bp	Invitrogen (Life Technologies)	# K4950-00 https://tools.thermofisher.com/content/sfs/manuals/virapower_lentiviral_system_man.pdf

Cultured Cells

Name	Vendor or Source	Sex (F, M, or unknown)	Persistent ID / URL
HPASMCs	ScienCell Research Laboratories	Unknown	#3110 https://sciencellonline.com/human-pulmonary-artery-smooth-muscle-cells/
Primary IPAH HPASMCs	UGMLC Giessen Biobank	3 M, 3 F	N/A
Primary Donor HPASMCs	UGMLC Giessen Biobank	3 Males, 3 Females	N/A
HPAECs	PromoCell	Unknown	#C-12241 https://promocell.com/product/human-pulmonary-artery-endothelial-cells-hpaec/
CDK4 KO HAP1 cells	Horizon Discovery	Unknown	#HZGHC000044c011 https://horizondiscovery.com/en/engineered-cell-lines/products/human-hap1-knockout-cell-lines?nodeid=entrezgene-1019&catalognumber=HZGHC000044c011
Parental WT HAP1 cells	Horizon Discovery	Unknown	#C631 https://horizondiscovery.com/engineered-cell-lines/products/hap1-parental-cell-lines
MCF7 cells	ATCC (American Type Culture Collection)	F	https://www.atcc.org/products/htb-22
HeLa	ATCC (American Type Culture Collection)	F	https://www.atcc.org/products/ccl-2

Data & Code Availability

Description	Source / Repository	Persistent ID / URL
Affymetrix microarray raw data and processed excel file with the relative expression values for approximately top 1000 transcripts in all samples	Gene Expression Omnibus, NCBI (accession number GSE244830)	https://www.ncbi.nlm.nih.gov/geo/query/acc.cgi?acc=GSE244830

DOI [to be added]

Other

Description	Source / Repository	Persistent ID / URL
Dulbecco's Modified Eagle Medium (DMEM)	ThermoFisher Scientific	#11965092 https://www.thermofisher.com/order/catalog/product/11965092?SID=srch-srp-11965092
FBS	ScienCell Research Laboratories	#0500 https://sciencellonline.com/fetal-bovine-serum-1558/
Smooth Muscle Cell Growth Medium-2	PromoCell	#C-39262 https://promocell.com/product/smooth-muscle-cell-growth-medium-2/
Endothelial Cell Growth Medium-2	PromoCell	#C-22211 https://promocell.com/product/endothelial-cell-growth-medium-2/
Iscove's Modified Dulbecco's Medium	ThermoFisher Scientific	#12440053 https://www.thermofisher.com/order/catalog/product/12440053?SID=srch-srp-12440053
FBS	PAN Biotech	#P40-39500 https://www.pan-biotech.de/en/FBS-Good-EU-approved-regions-filtrated-bovine-serum-0.2-m-sterile-filtered/P40-37500
Auranofin	Enzo Life Sciences	#BML-EI206 https://www.enzolifesciences.com/BML-EI206/auranofin/
H_2O_2	Merck	#H1009 https://www.sigmaaldrich.com/GB/en/product/sigma/h1009
Lipofectamine 3000	Invitrogen	#L3000008 https://www.thermofisher.com/order/catalog/product/L3000008?SID=srch-srp-L3000008
Lipofectamine 2000	Invitrogen	#11668019 https://www.thermofisher.com/order/catalog/product/11668500?SID=srch-srp-11668500
Zeba Spin desalting column	ThermoFisher Scientific	#89882 https://www.thermofisher.com/order/catalog/product/89882?SID=srch-srp-89882
Q5 mutagenesis kit	New England Biolabs	#E0554S https://www.neb.com/en-gb/products/e0554-q5-site-directed-mutagenesis-kit#Product%20Information
Phusion mutagenesis kit	ThermoFisher Scientific	#F541 https://www.thermofisher.com/order/catalog/product/F541?SID=srch-srp-F541
Cyclin D1 Silencer Select siRNA	ThermoFisher Scientific	#4390824 Assay ID 229 https://www.thermofisher.com/order/genome-database/details/sirna/s229?CID=&ICID=&subtype=sirna_silencer_select
Scrambled Silencer Select siRNA (Negative control no.1)	ThermoFisher Scientific	#4390843 https://www.thermofisher.com/order/catalog/product/4390844?SID=srch-srp-4390844

DOI [to be added]

Pierce BCA Protein Assay Kit	ThermoFisher Scientific	#23225 https://www.thermofisher.com/order/catalog/product/23225?SID=srch-srp-23225
Recombinant cyclin D1-His	LSBio (Lifespan Biosciences)	#LS-G20572 https://www.lsbio.com/proteins/human-ccnd1-cyclin-d1-protein-recombinant-6his-n-terminus-full-length-ls-g20572/20572
Recombinant CDK4-His	Invitrogen	#RP-75530 https://www.thermofisher.com/proteins/product/Human-CDK4-Recombinant-Protein/RP-75530
Recombinant cyclin D1-CDK4 GST-tag	Invitrogen	#PV4400 https://www.thermofisher.com/proteins/product/Human-CDK4-Cyclin-D1-GST-Tag-Recombinant-Protein/PV4400
Recombinant Rb	Abcam	#Ab83205 https://www.abcam.com/products/proteins-peptides/recombinant-human-rb-protein-ab83205.html
Recombinant RB GST-tag protein fragment	Merck	#SRP0256 https://www.sigmaaldrich.com/GB/en/product/sigma/srp0256
Complete EDTA Protease inhibitor tablets	Roche	#11836170001 https://www.sigmaaldrich.com/GB/en/product/roche/11836170001
Anti-FLAG M2 Affinity Gel	Merck	#A2220 https://www.sigmaaldrich.com/GB/en/product/sigma/a2220
Palbociclib	LKT laboratories	#P0344 https://lktlabs.com/product/palbociclib-isethionate/
Propidium Iodide Flow cytometry kit	Abcam	#ab139418 https://www.abcam.com/products/assay-kits/propidium-iodide-flow-cytometry-kit-ab139418.html
Rapid Extract PCR kit	PCR biosystems	#PB10.24-08 https://pcrbio.com/products/dna-extraction/pcrbio-rapid-extract-pcr-kit
MunI (MfeI) enzyme	Thermo Fisher Scientific	#ER0751 https://www.thermofisher.com/order/catalog/product/ER0751
CDK4 forward and reverse primers	Integrated DNA technologies	5'- GTGGACCGATCTTTGCAATAG-3'<br>3'- CGGAACATCTCTGCAAAGATAC-3'<br>www.idtdna.com
Collagenase/Dispase	Roche	#10269638001 https://www.scientificlabs.co.uk/product/10269638001
70-µm nylon strainer	Corning	#CLS431751-50EA https://www.scientificlabs.co.uk/product/CLS431751-50EA
30-µm nylon strainer	Miltenyi Biotec	#130-098-458 https://www.miltenyibiotec.com/GB-en/products/macs-smartstrainers.html

DOI [to be added]

CD31 MicroBeads, mouse	Miltenyi Biotec	#130-097-418 https://www.miltenyibiotec.com/GB-en/products/cd31-microbeads-mouse.html
autoMACS Rinsing Solution	Miltenyi Biotec	#130-091-222 https://www.miltenyibiotec.com/GB-en/products/automacs-rinsing-solution.html
MACS BSA Stock Solution	Miltenyi Biotec	#130-091-376 https://www.miltenyibiotec.com/GB-en/products/macs-bsa-stock-solution.html
LS Columns	Miltenyi Biotec	#130-042-401 https://www.miltenyibiotec.com/GB-en/products/ls-columns.html
Midi MACS separator	Miltenyi Biotec	#130-042-302 https://www.miltenyibiotec.com/GB-en/products/midimacs-separator-and-starting-kits.html
MACS multistand	Miltenyi Biotec	#130-042-303 https://www.miltenyibiotec.com/GB-en/products/midimacs-separator-and-starting-kits.html
Gelatin solution	Merck (Sigma-Aldrich)	#G1393-20ML https://www.sigmaaldrich.com/GB/en/product/sigma/g1393
Endothelial Cell Growth Supplement without FBS (5ml-2 parts)	Merck (Sigma-Aldrich)	#211F-GS https://www.sigmaaldrich.com/GB/en/product/sigma/211fgs
Heparin sodium salt	Merck (Sigma-Aldrich)	#H3149-100KU https://www.sigmaaldrich.com/GB/en/product/sial/h3149
Penicillin/Streptomycin	Thermo Fisher Scientific	#15140122 https://www.thermofisher.com/order/catalog/product/15140122
E-Plate 16 PET	Agilent Technologies	#300600890 https://www.agilent.com/store/en_US/Prod-300600890/300600890
Phosphate buffered saline pH 7.4	Thermo Fisher Scientific	#10010023 https://www.thermofisher.com/order/catalog/product/10010023
Trypsin-EDTA solution	Merck (Sigma-Aldrich)	#T4049 https://www.sigmaaldrich.com/GB/en/product/sigma/t4049
Medium 199	Corning	#10-060-CV https://ecatalog.corning.com/life-sciences/b2b/UK/en/Media,-Sera,-and-Reagents/Classical-Media/Medium-199/Corning%C2%AE-Medium-199-(Modification)/p/10-060-CV
Amphotericin B	Gibco	#15290026 https://www.thermofisher.com/order/catalog/product/15290026?SID=srch-srp-15290026

DOI [to be added]

Iron (II,III) oxide	Merck (Sigma-Aldrich)	#310069 https://www.sigmaaldrich.com/GB/en/product/aldrich/310069
Collagenase	Merck (Sigma-Aldrich)	#C5138 https://www.sigmaaldrich.com/GB/en/product/sigma/c5138
VECTASTAIN Elite ABC kit	Vector Laboratories	#PK-6102 https://vectorlabs.com/products/vectastain-elite-abc-hrp-kit-mouse-igg/
Diaminobenzidine	Vector Laboratories	#SK-4100 https://vectorlabs.com/products/dab-hrp-substrate/
RNeasy Plus Universal kit	Qiagen	#73404 https://www.qiagen.com/us/products/discovery-and-translational-research/dna-rna-purification/rna-purification/total-rna/rneasy-universal-kits
Encore Biotin module (NuGEN)	N/A	https://bmlabosis.com/uploads/3668b6c733804f5a9145421ebd17dbff.pdf
Pentoject® 200mg/ml Solution	Animalcare Limited	https://www.animalcare.co.uk/anaesthetics-analgestics/pentoject/
QIAprep Spin miniprep kit	Qiagen	#27104 https://www.qiagen.com/us/products/discovery-and-translational-research/dna-rna-purification/dna-purification/plasmid-dna/qiaprep-spin-miniprep-kit
HISpeed Plasmid Maxi kit	Qiagen	#12662 https://www.qiagen.com/us/products/discovery-and-translational-research/dna-rna-purification/dna-purification/plasmid-dna/hispeed-plasmid-kits?catno=12662
SU5416	BioTechne / Tocris	#3037 https://www.tocris.com/products/su-5416_3037
SU 5416 (Semaxanib), VEGFR-2 inhibitor	Abcam	#ab145056 https://www.abcam.com/en-hr/products/biochemicals/su-5416-semaxanib-vegfr-2-inhibitor-ab145056
Alzet mini-pumps, model 2002	Alzet	#0000296 https://www.alzet.com/wp-content/uploads/2019/06/Specs-2002.pdf

DOI [to be added]
