# Supplementary material for: Cyclin D-CDK4 Disulfide Bond Attenuates Pulmonary Vascular Cell Proliferation
Source: Circ Res. 2023 Nov 13;133(12):966–88. doi: 10.1161/CIRCRESAHA.122.321836 (PMC10699508; doi:10.1161/CIRCRESAHA.122.321836)

Figure 1 - Full Unedited blots

Figure 1B

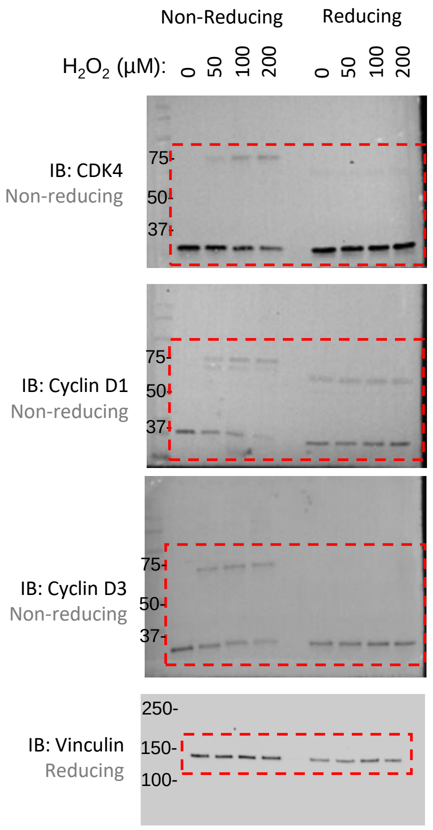

Figure 1D

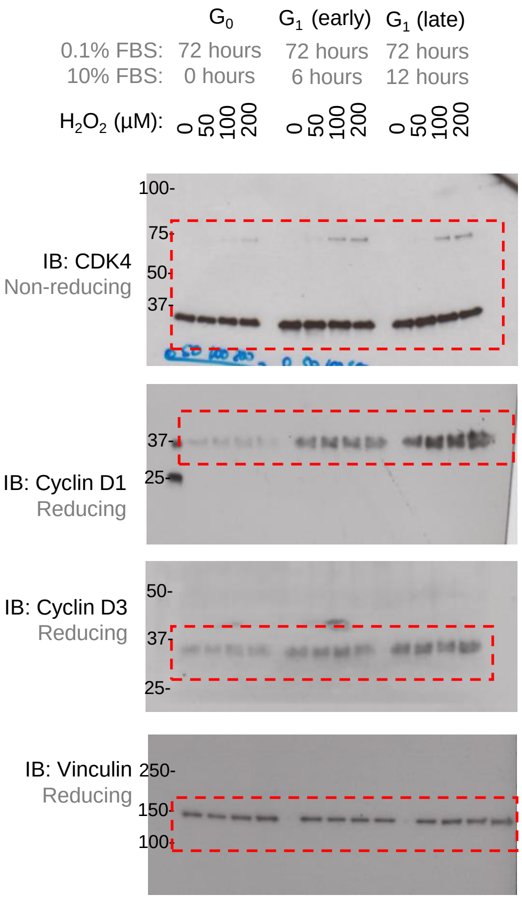

Figure 1F

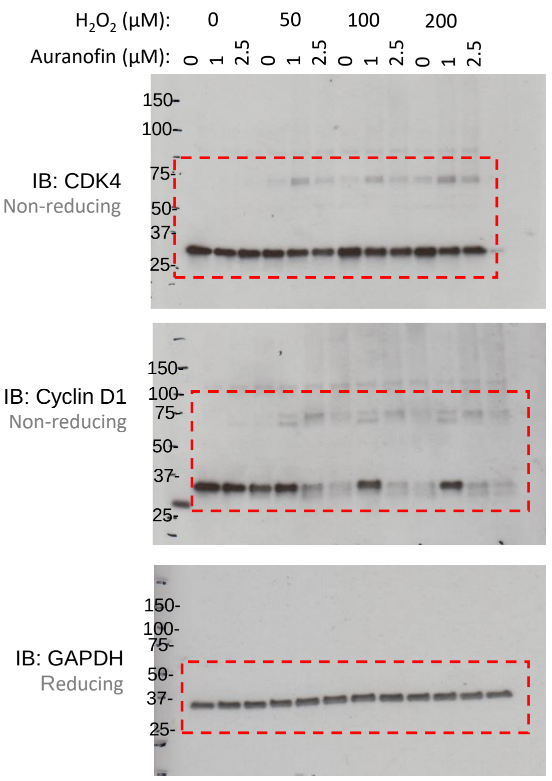

Figure 2 - Full Unedited blots

Figure 2B

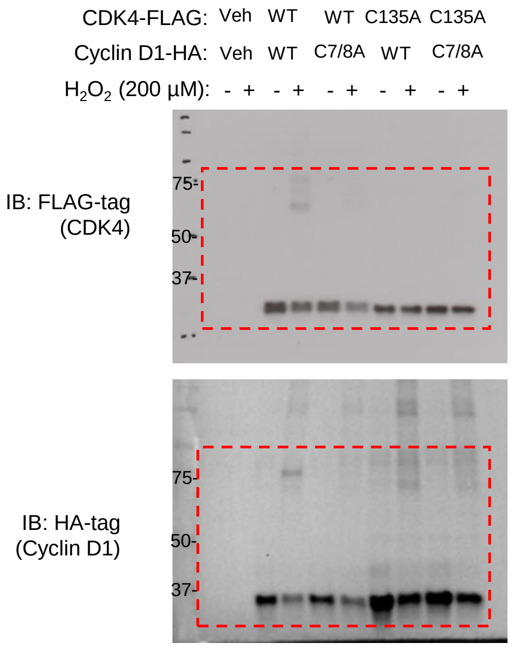

Figure 2F

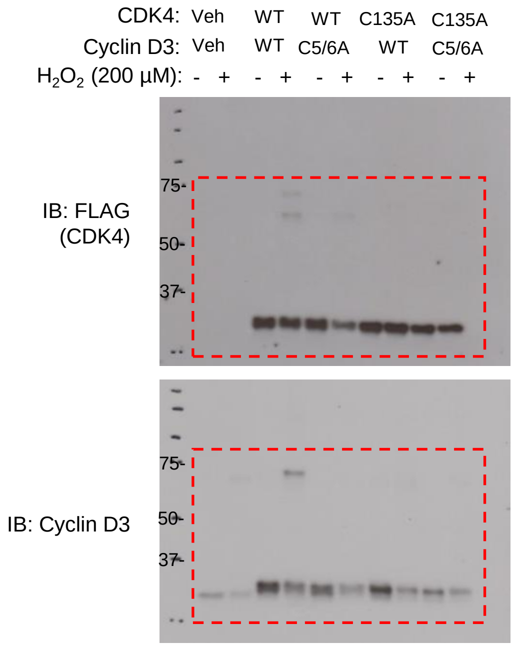

Figure 3 - Full Unedited blots

Figure 3A

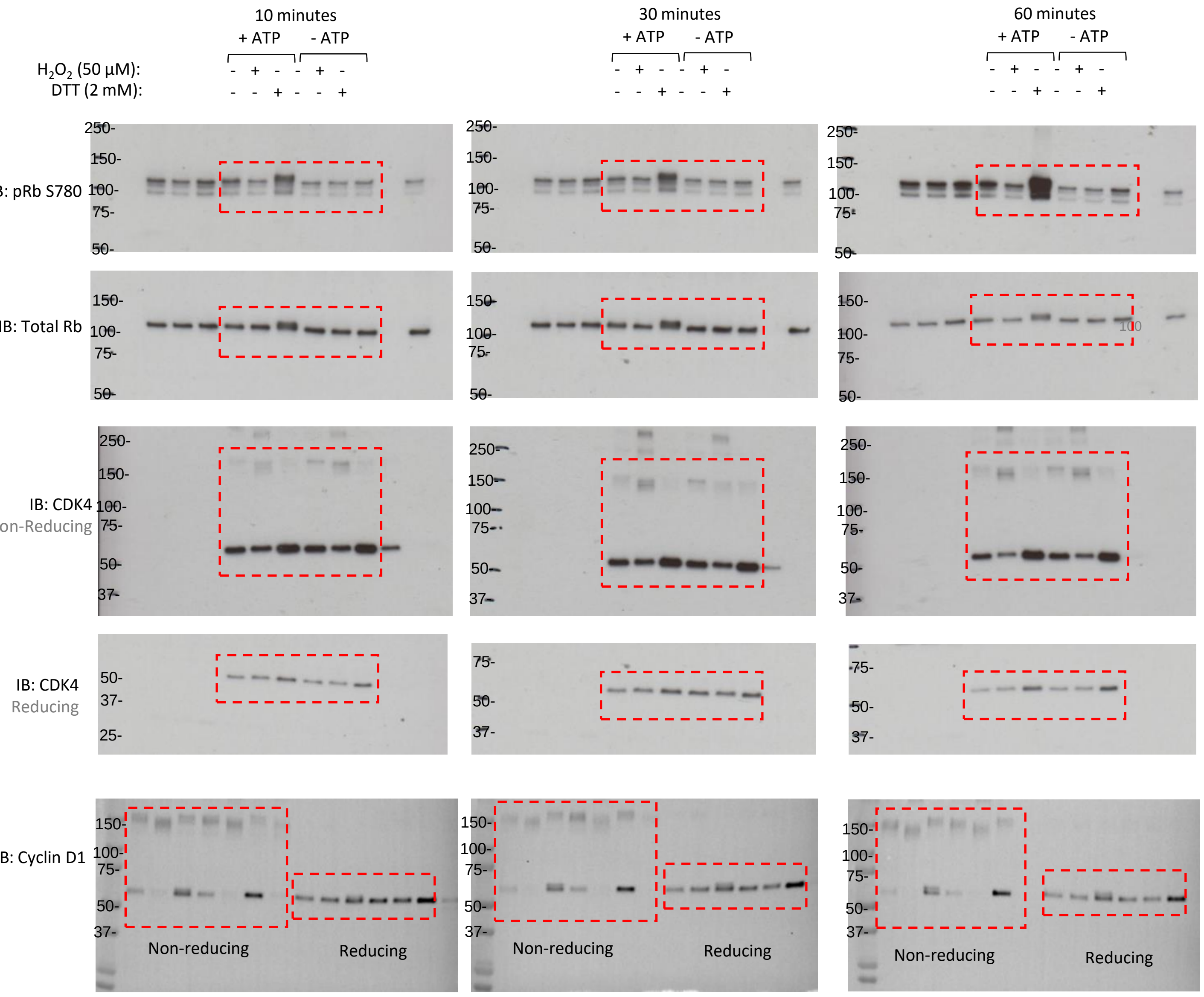

Figure 4 - Full Unedited blots

Figure 4A

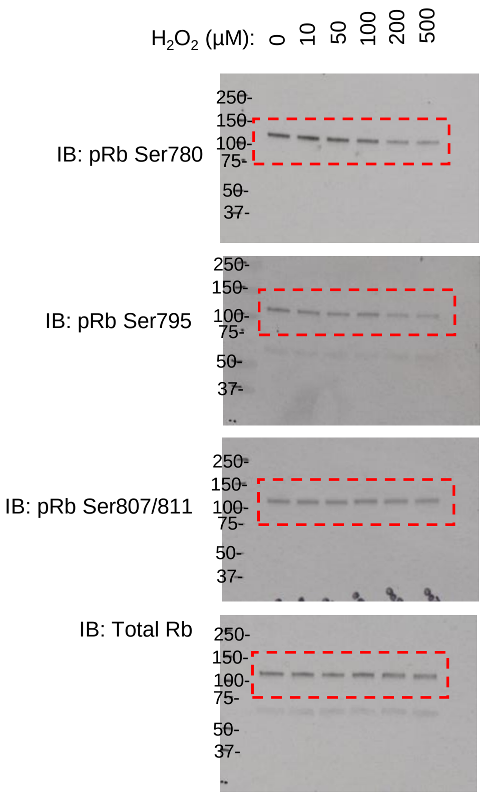

Figure 5 - Full Unedited blots

Figure 5A

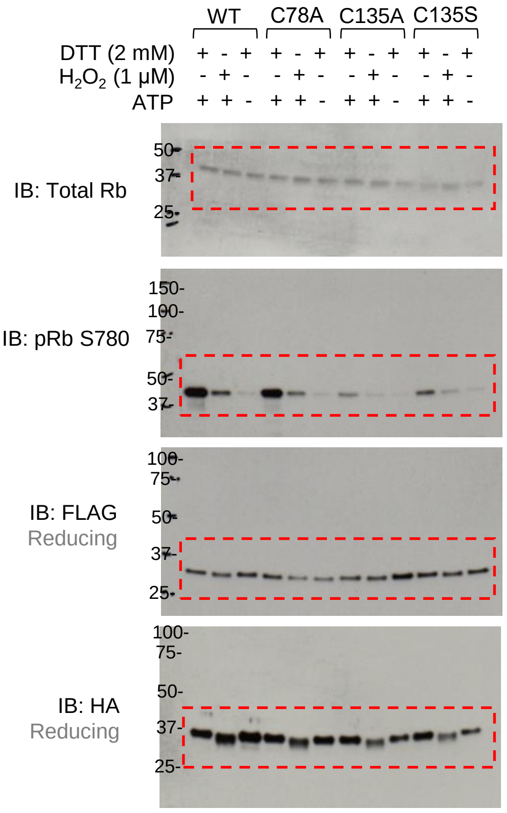

Figure 5C

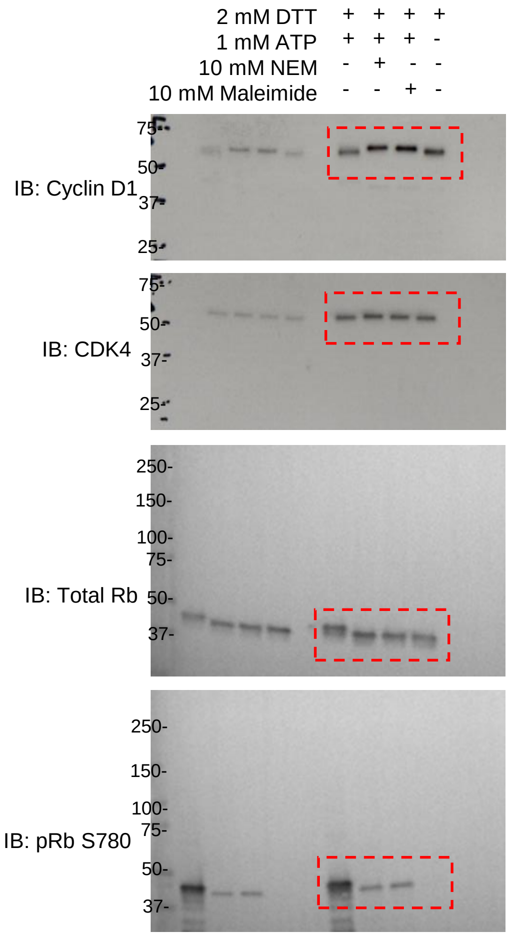

Figure 5E

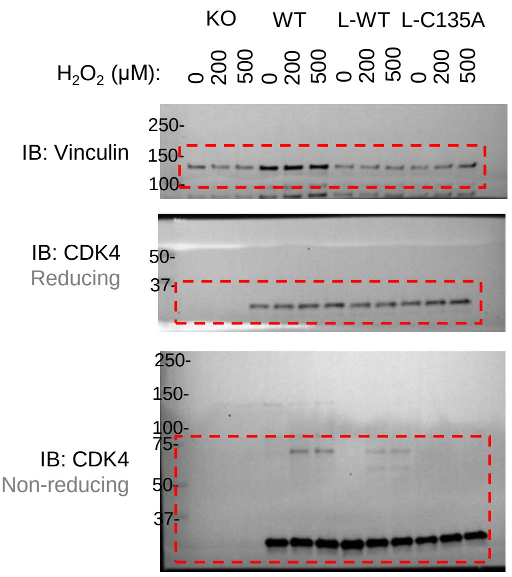

Figure 7 - Full Unedited blots

Figure 7A

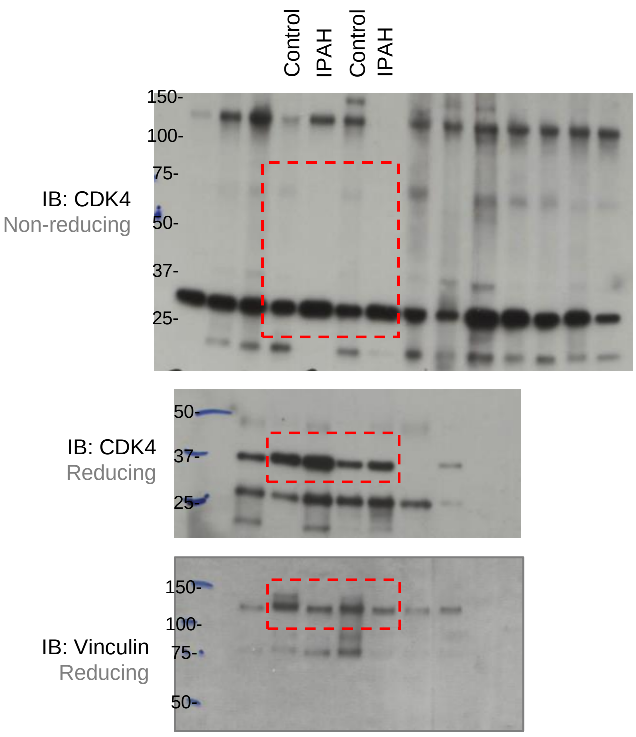

Figure 7B

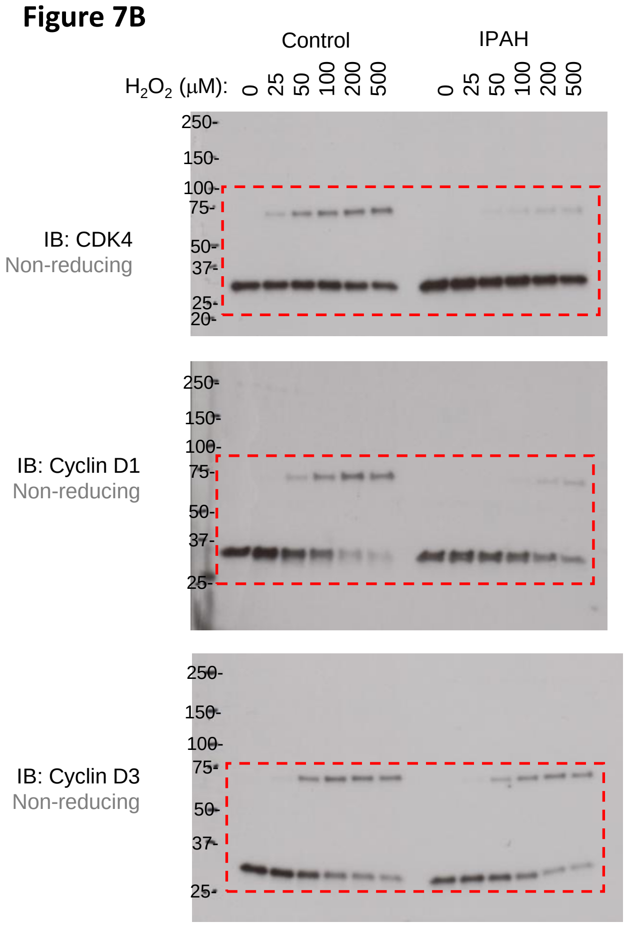

Figure 7C

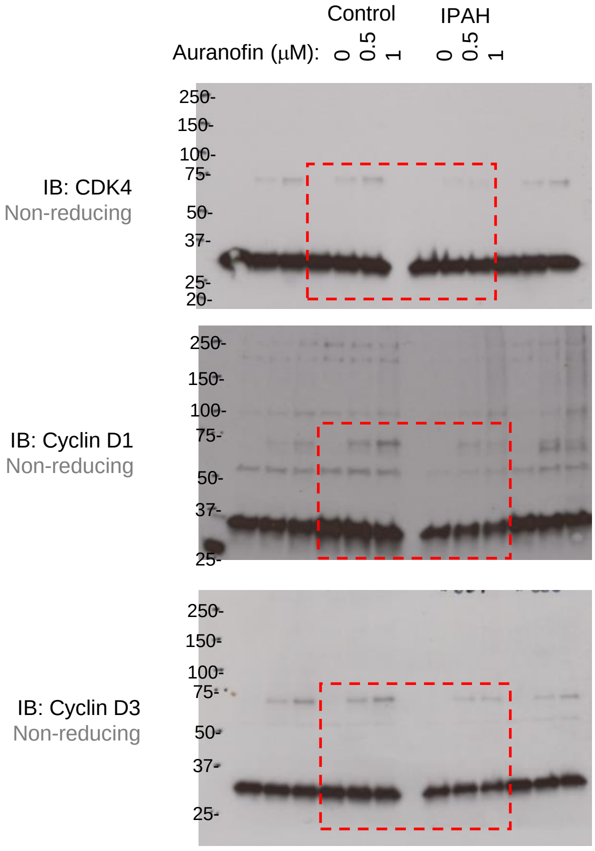

Figure 8 - Full Unedited blots

Figure 8A

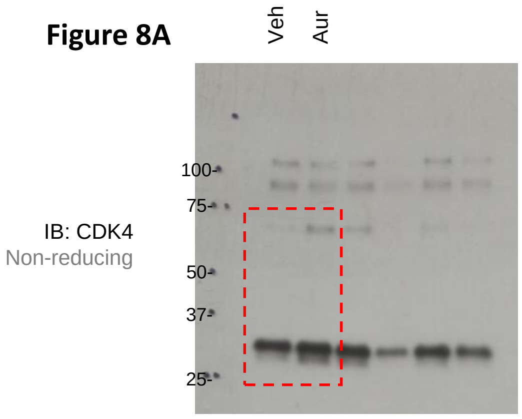

Supplementary Figure 1 - Full Unedited blots

Figure S1A

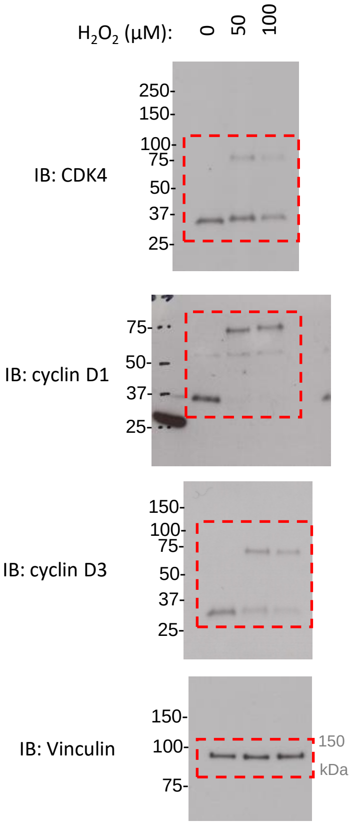

Figure S1B

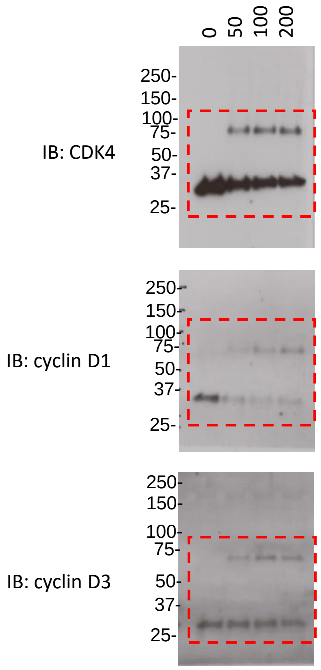

Figure S1C

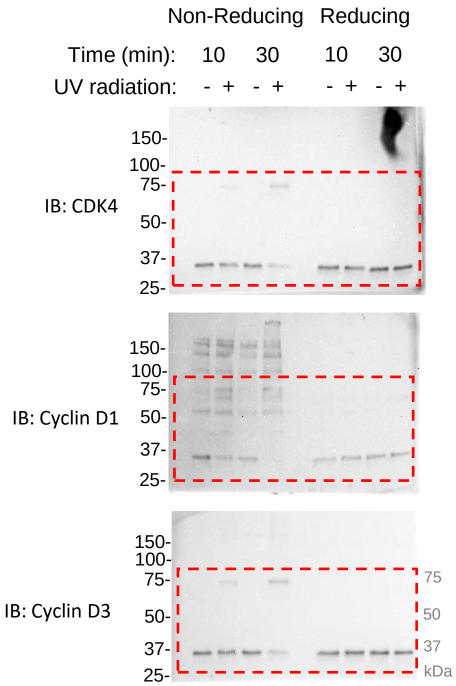

Figure S1D

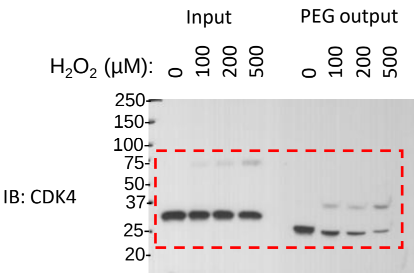

Supplementary Figure 2 - Full Unedited blots

Figure S2A

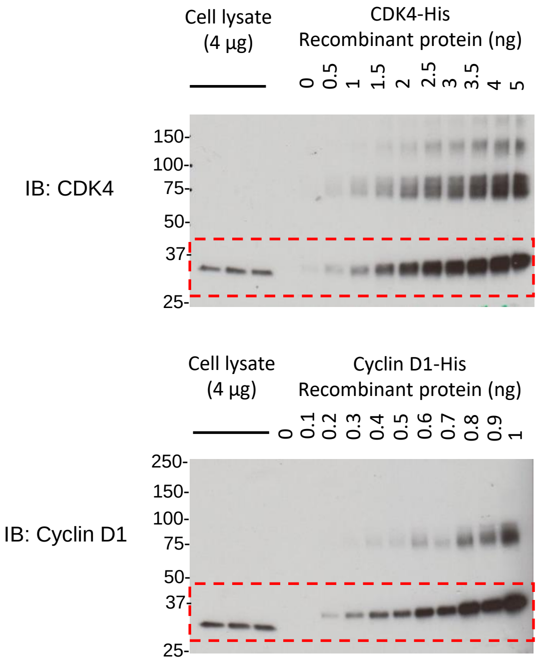

Figure S2C

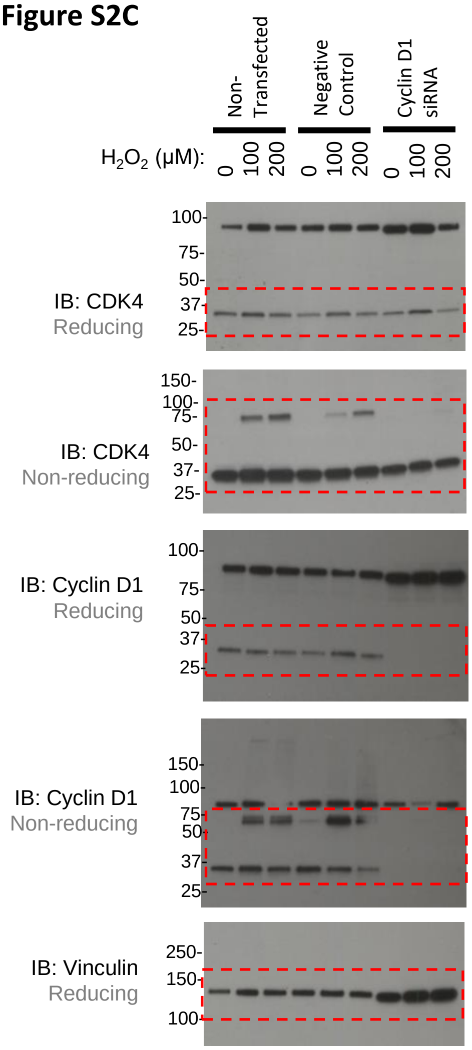

Figure S2B

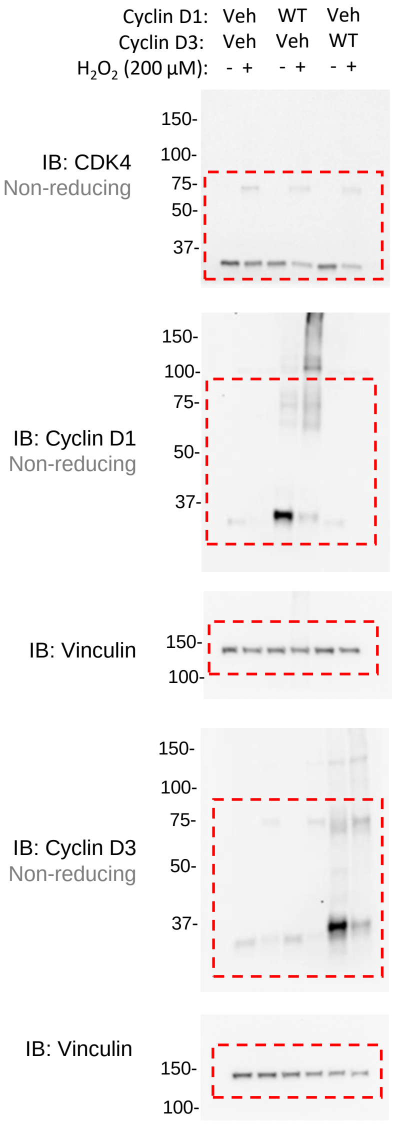

Supplementary Figure 3 - Full Unedited blots

Figure S3A

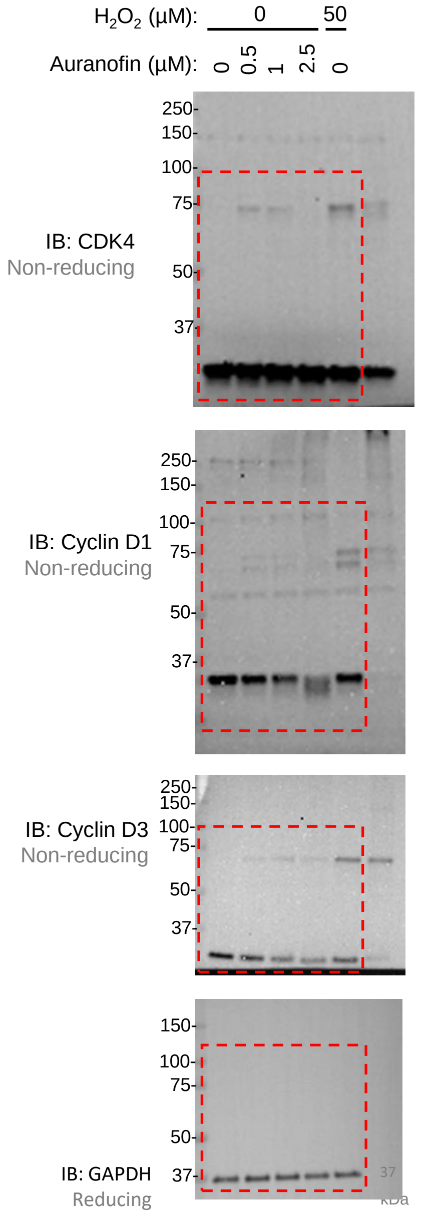

Figure S3B

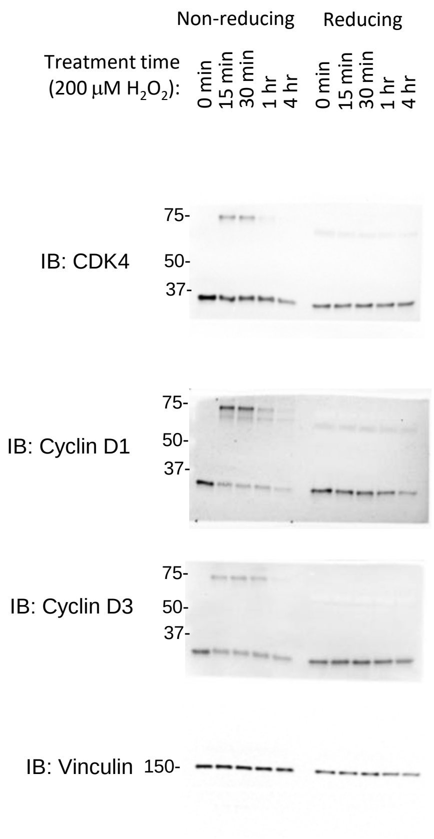

Supplementary Figure 4 - Full Unedited blots

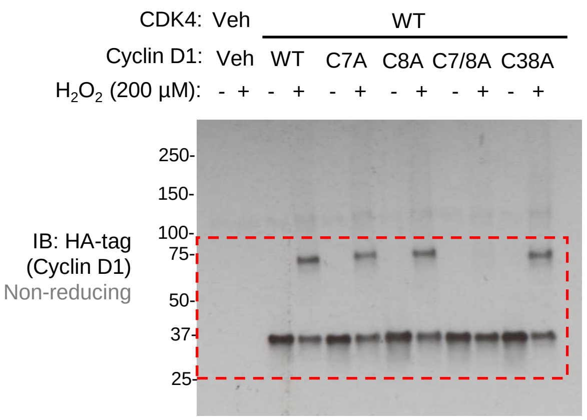

Supplementary Figure 8 - Full Unedited blots

Figure S8A

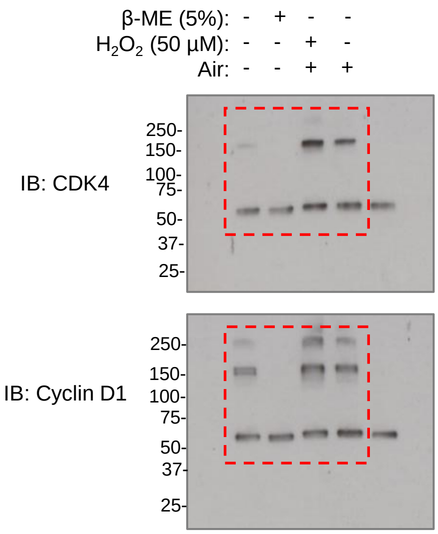

Supplementary Figure 12 - Full Unedited blots

Figure S12A

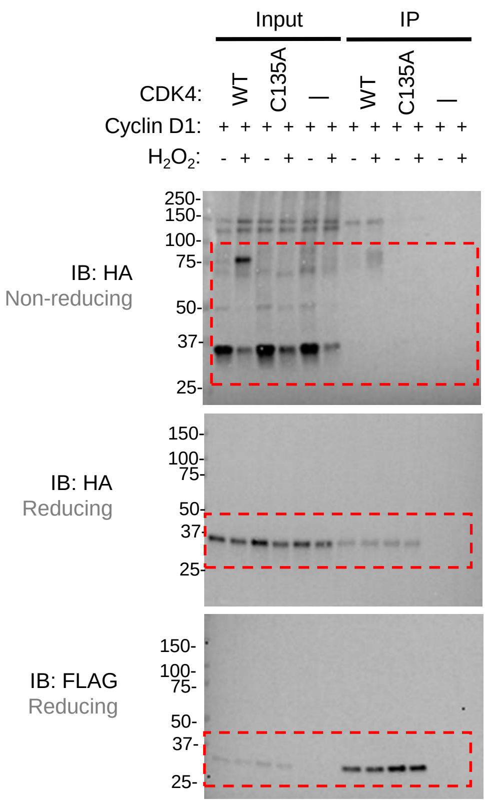

Figure S12B

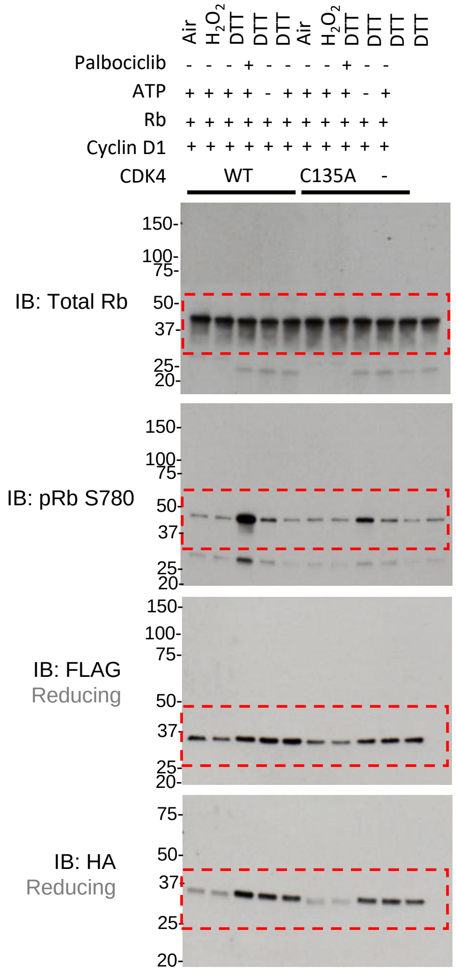

Supplementary Figure 12 - Full Unedited blots

Figure S12C

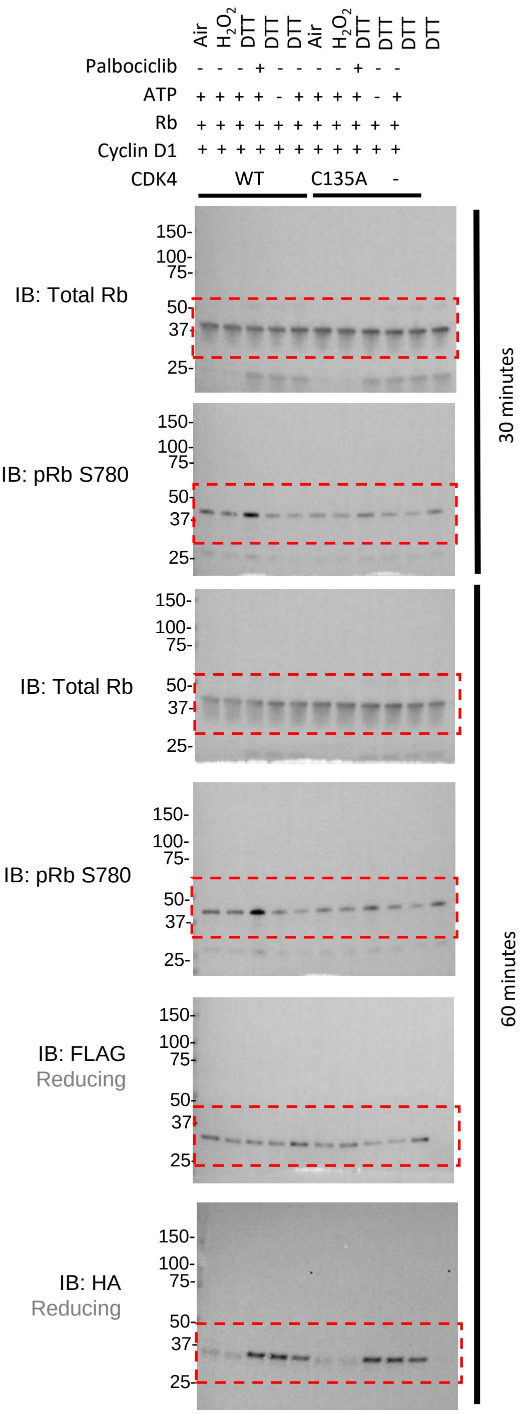

Figure S12D

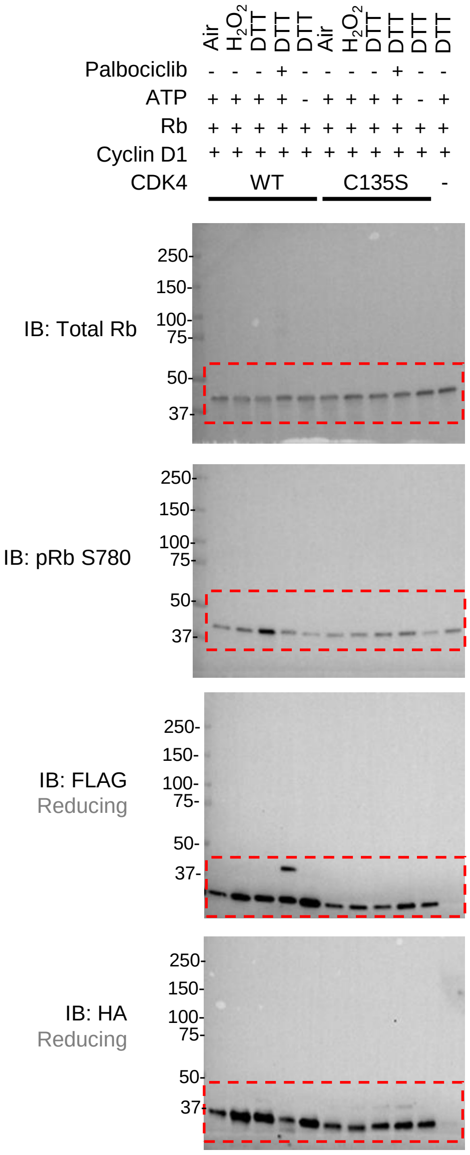

Supplementary Figure 13 - Full Unedited blots

Figure S13A

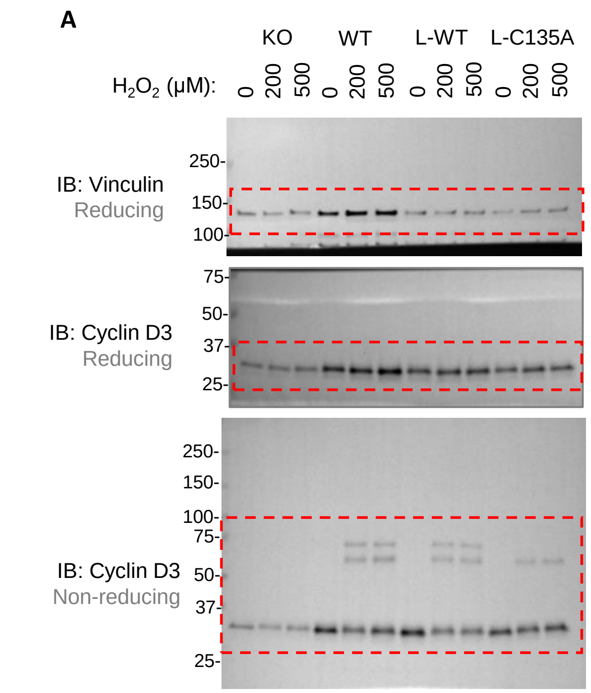

Figure S13C

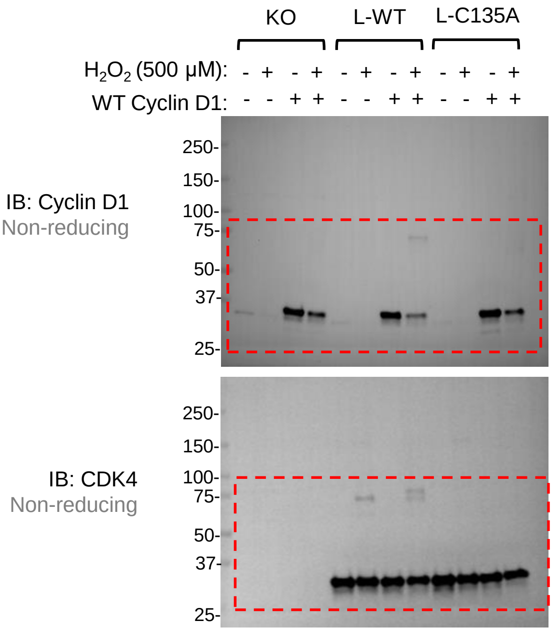

Supplement: Supplementary file 4 [file res-133-0966-s004.pdf]
